# Investigating the impact and mechanism of Licochalcone B derivative CTG12 on NLRP3 inflammasome

**DOI:** 10.1186/s12964-026-02741-2

**Published:** 2026-02-18

**Authors:** Shuyi Bian, Zhi-E Fang, Lichen Wang, Jiayi Wang, Ming Dong, Nan Yang, Jiabo Wang, Ling Li, Guang Xu

**Affiliations:** 1 School of Traditional Chinese Medicine, Capital Medical University, Beijing, 100069 China; 2 National Key Laboratory of Advanced Biotechnology, Academy of Military Medical Sciences, Beijing, 100071 China; 3 Department of Pharmacy, Chongqing Hospital of Traditional Chinese Medicine, Chongqing, 400021 China

**Keywords:** CTG12, NLRP3 inflammasome, ASC oligomerization, NLRP3-ASC interaction, Inflammatory diseases

## Abstract

**Background:**

Aberrant activation of NLRP3 inflammasome has been associated with a variety of human inflammatory diseases, but no small molecule inhibitors of NLRP3 were applied in clinical practice. Our research group has previously shown that licochalcone B is an effective NLRP3 inflammasome inhibitor, however, its IC50 value is relatively high compared to compounds such as MCC950. Modifying bioactive natural products to find NLRP3 inflammasome inhibitors with stronger potency and higher specificity is a direction worthy of investigation.

**Purpose:**

Our research aims to evaluate the impact of various licochalcone B derivatives on NLRP3 inflammasome, to screen for derivatives with better potency and elucidate the underlying mechanisms.

**Methods:**

We investigated the effects of licochalcone B and its derivatives on NLRP3 inflammasome by assessing the production of active caspase-1 and interleukin 1β (IL-1β). To elucidate the mechanism of CTG12, we employed co-immunoprecipitation. Furthermore, we evaluated CTG12 in LPS-induced acute systemic inflammation mouse models.

**Results:**

The findings demonstrated that licochalcone B and its derivatives effectively inhibit NLRP3 inflammasome. CTG12 exhibits the most potent inhibitory activity, showing approximately tenfold increased effects compared to licochalcone B. Mechanistic investigations reveal that while CTG12 does not affect potassium (K⁺) efflux, calcium (Ca^2^⁺) influx, or mitochondrial reactive oxygen species (mtROS) production, it suppressed NLRP3 assembly by interfering with the ASC-NLRP3 interaction and the NLRP3-dependent ASC oligomerization process. In vivo, CTG12 provides significant therapeutic benefits in LPS-induced acute systemic inflammation models.

**Conclusion:**

Our results indicated that structurally modified licochalcone B derivative CTG12 inhibits NLRP3 assembly by interfering with the ASC-NLRP3 interaction, thereby inhibiting the NLRP3-dependent ASC oligomerization process in NLRP3 inflammasome activation. These studies would show that CTG12 is a valuable small molecule inhibitor that holds promise as a high-value drug candidate for the treatment of NLRP3-mediated inflammatory diseases.

**Graphical Abstract:**

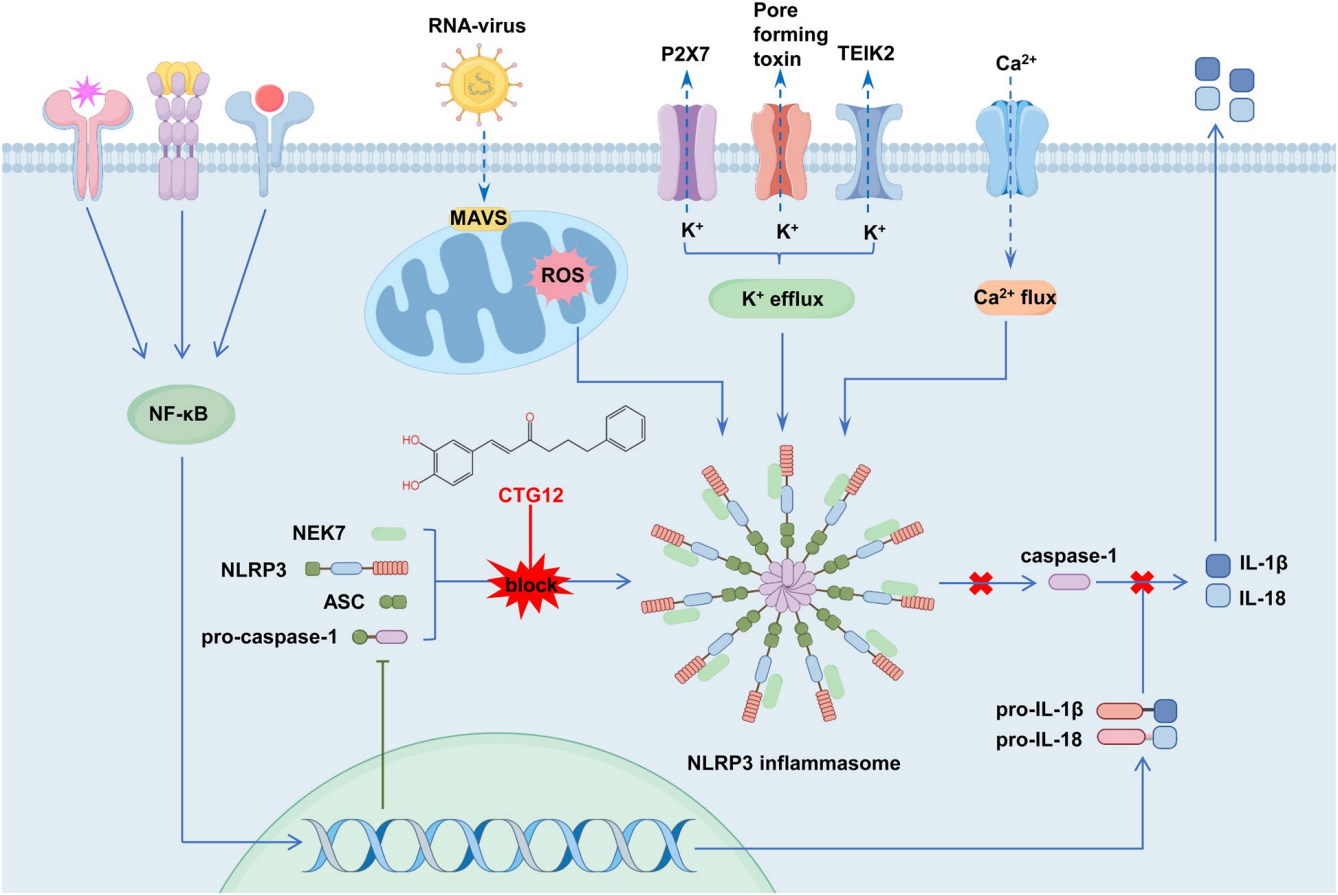

**Supplementary Information:**

The online version contains supplementary material available at 10.1186/s12964-026-02741-2.

## Introduction

Inflammation is an important and protective immune response that occurs in response to external stimuli [1] The occurrence of inflammation is closely related to the activation of immune system, especially the innate immune system [2]. This innate defense system serves as the organism initial protective barrier, playing a critical role in homeostatic regulation through rapid response to pathogenic challenges. Different pattern recognition receptors (PRRs) in the innate immune system recognize pathogen-associated molecular patterns (PAMPs) and danger-associated molecular patterns (DAMPs). Some of these pattern recognition receptors can recruit downstream adaptor proteins to form a complex called inflammasomes, which activate pro-caspase-1, leading to pyroptosis and generation of IL-1β and IL-18, causing inflammatory responses [3]. Moderate inflammatory response helps the body quickly and effectively remove various pathogenic microorganisms and potential "danger signals", which plays a vital part in maintaining the health. However, abnormal inflammasome activation is critically involved in the development of many diseases, including type 2 diabetes (T2D), gout, atherosclerosis, cryopyrin-associated autoinflammatory syndromes (CAPS), septic shock and neurodegenerative diseases [4].

The inflammatory response is triggered by the induction of a variety of inflammasomes. Up to now, various inflammasomes have been reported, such as NLRP3, NLRP1, AIM2 and NLRC4. NLRP3 inflammasome is the most widely researched among these inflammasomes. The NLRP3 inflammasome, a key component of the innate immune system, is a multiprotein complex consisting of the NLR family member NLRP3, the adaptor protein ASC and pro-caspase-1 [5]. The two-step process of NLRP3 inflammasome activation in macrophages consists of priming and activation. The priming step (signal 1) is triggered by inflammatory stimuli, leading to NF-κB activation and subsequent upregulation of NLRP3 and pro-IL-1β expression [6]. In the activation step (signal 2), ASC and pro-caspase-1 are recruited to form an active NLRP3 inflammasome. This activation leads to the cleavage of pro-caspase-1, generating active caspase-1. Active caspase-1 cleave Gasdermin D (GSDMD) to trigger pyroptosis. In addition, it processes pro-IL-1β and pro-IL-18 to form active IL-1β and IL-18 [7–10].

Recently, the link between the NLRP3 inflammasome and a wide range of diseases has sparked significant interest among researchers to develop potent NLRP3 inflammasome inhibitors and many NLRP3 inflammasome inhibitors have been developed [11]. However, most inhibitors are nonspecific, less effective and have not been applied in clinical practice because of various problems. For example, MCC950 is among the most specific and potent inhibitors of the NLRP3 inflammasome [12], but it was discontinued in Phase II clinical trials because of its hepatotoxicity. Therefore, it is necessary to find safe and effective NLRP3 inflammasome inhibitors.

Licorice has been widely used in traditional medicine for its expectorant and anti-inflammatory effects. It is one of the most significant herbs and a key focus of research in Chinese medicine [13]. The research group has previously studied licochalcone B and Echinatin in licorice, and found that licochalcone B and Echinatin are potential and effective NLRP3 inflammasome inhibitors [14, 15]. However, they are almost completely inhibited at a concentration of 40 μM, and the potency is not high. In addition, Echinatin does not specifically inhibit NLRP3 inflammasome activation. Scientific research indicates that altering the structure of natural bioactive molecules is a key step in developing new drugs [16]. Through such modifications, lead compounds are often optimized to exhibit greater potency, higher target affinity, and potentially improved safety profiles, thereby increasing their promise as clinical candidates. Therefore, we can structurally engineer natural products to find NLRP3 inflammasome inhibitors with better biological effects and higher specificity. In this research, the impact of licochalcone B derivatives on NLRP3 inflammasomes was studied and its potential mechanism was deeply explored.

## Materials and methods

### Materials

#### Animals

All animal experiments were undertaken following the granting of approval from the Animal Ethics Committee of Capital Medical University. The record number of the laboratory animal business license is SCXK (Beijing) 2023–0008. C57BL/6 J mice were obtained from SPF GemPharmatech. Ltd. Mice were maintained in a pathogen-free environment. All experiments used age- and sex-matched mice.

#### Reagents

Lico chalcone B (HY-N0373), MCC950 (HY-12815A), Nigericin (HY-100381), murine macrophage colony-stimulating factor (M-CSF) (HY-P7085) were purchased from MedChemExpress (USA). ATP, SiO_2_, poly (dA: dT), poly (I:C) and phorbol-12-myristate-13-acetate (PMA) were procured from Sigma-Aldrich (Germany). Dimethyl sulfoxide (DMSO) was purchased from Solarbio (China). Ultrapure lipopolysaccharide (LPS) and Pam3CSK4 were from InvivoGen (France). Salmonella and NLRP3-Flag plasmids were provided by Dr. Tao Li from the National Center for Biomedical Analysis (NCBA) (Beijing, China). CellTiter-Glo Luminescent Cell Viability Assay (G7572), Caspase-1 activity analysis kit (G9951) and LDH analysis kit (G1780) were procured from Promega (USA). Anti-human IL-1β (p17, 12242S), anti-Phospho-IRF3 (Ser396) (4947), caspase-1 antibody (4199S) and anti-ASC antibody (sc-22, 514-R) were obtained from Cell Signaling Technology (Germany). Active caspase-1 antibody (AG-20B-0042) and NLRP3 antibody (AG-20B-0014) were obtained from Adipogen (USA). Anti-NEK7 antibody (ab133514), and anti-mouse IL-1β antibody (AF-401-NA) were purchased from Abcam and R&D Systems. Lipofectamine 2000 (11,668–019, Invitrogen (California, USA), anti-IRF3 (11,312–1-AP), anti-STING (19,851–1-AP), anti-Lamin B antibody (66,095–1-Ig) and anti-strep tag (DYKDDDDK) antibody (20,543–1-AP) were gotten from Proteintech (USA). Phosphate buffer saline (CC008) was purchased from Macgene. Disuccinimidyl suberate (DSS) (21,655) was obtained from Thermo Scientific (USA). Mouse IL-18 Elisa kit (ML002294) was purchased from mlbio (China). Mouse IL-1β Elisa kit (MLB 00 C) was purchased from R&D systems. Mouse CXCL1/KC Elisa kit (EK296/2–96) was purchased from Liankebio (China). Mouse TNF-α Elisa kit (1,217,202), Mouse IL-6 Elisa kit (1,210,602), Human TNF-α Elisa kit (1,117,202) and Human IL-1β Elisa kit (1,110,122) were purchased from Dakewe.

### Methodology

#### Cell preparation and stimulation

BMDMs were extracted from the bone marrow of the femurs of 8-week-old male mice and maintained in Dulbecco’s modified Eagle’s medium (DMEM) containing 10% fetal bovine serum, 50 ng/ml recombinant M-CSF (416-ML-050, R&D Systems) and 1% penicillin–streptomycin for 5 days. THP1 cells were incubated overnight in RPMI-1640 medium containing 10% fetal bovine serum and 1% penicillin–streptomycin, along with 100 nM PMA, prior to stimulation. HEK-293 T was cultured in DMEM medium containing 10% fet al bovine serum and 1% penicillin–streptomycin. Cells were cultured at 37 °C in a humidified incubator (HeracellTM 150i, Thermo Scientific (Massachusetts, USA)) with 5% CO_2_.

In the experiment involving activation of the inflammasome pathway, BMDMs were plated in 12-well culture dishes at a density of 1.2 × 10⁶ cells/ml, then allowed to grow overnight (12–18 h), then, murine BMDMs were stimulated with 50 ng/ml LPS and 400 ng/ml Pam3CSK4 for 4 h. The cells were subsequently incubated with CTG12 for half an hour, followed by treatment with different stimulants, as follows: 10 μM nigericin for 25 min; 5 mM ATP for 45 min; 250 μg/ml SiO_2_, 200 ng/ml salmonella for 4 h; BMDMs were transfected for 4 h using Lipofectamine 2000 with either 2 μg/ml poly (I:C), 1 μg/ml ultra-LPS, or 2 μg/ml poly (dA: dT). THP1 cells were seeded in 12-well plates at a density of 1.3 × 10⁶ cells/ml, stimulated with 100 nM PMA to promote adherence, and cultured overnight. The cells were subsequently incubated with CTG12 for half an hour and then stimulated with 10 μM nigericin for 55 min.

In the experiment involving activation of the RIG-I-MAVS pathway, we seeded THP1 at a density of 1.2 × 10^6^ in 24-well plates overnight. The next day, we treated with varying concentrations of CTG12 (ranging from 0 to 10 μM) for an hour. Then, we used poly(I:C) to activate the RIG-I-MAVS pathway for 2 h.

#### LDH and caspase-1 activity assay

The Caspase-Glo 1 inflammasome assay and LDH reagents were prepared according to the instructions of the reagents, and the protocol for determining activity was determined according to the previously described method [17].

#### Western blot analysis

Proteins in the cell culture supernatants or tissue lysate was extracted for western blot analysis, as described previously [15].

#### Cell viability assays

Bone marrow-derived macrophages (BMDMs) and THP1 were plated in 96-well plates at one million cells per milliliter and cultivated overnight at 37 °C. The cells were then treated with varying concentrations of CTG12 (ranging from 0 to 80 μM) for six hours. Following this, ATP was used to stimulate the cells for 15 min, after which absorbance was measured at 450 nm.

#### ASC oligomerization assay

NLRP3 inflammasome activation procedure was the same as in Method 1.2.1. The cells were lysed using Triton buffer (150 mM NaCl, 50 mM Tris–HCl [pH 7.5], 0.5% Triton X-100, and EDTA-free protease inhibitor cocktail) for 15 min, harvested by scraping, and centrifuged at 6000 × g for 15 min at 4 °C. The resulting supernatant and pellet were designated as the Triton X-soluble and Triton X-insoluble fractions, respectively. The Triton X-insoluble fraction was washed, resuspended in 200 μL PBS followed by DSS crosslinking (2 mM, 30 min, 37 °C). After centrifugation at 6000 × g for 15 min at 4 °C, the pellet was collected and solubilized in 1 × Triton loading buffer for subsequent Western blot analysis.

#### ELISA

The levels of IL-1β and TNF-α in both mouse and human cells were quantified according to the manufacturer's protocol provided with the kit [18].

#### Measurement of mtROS

BMDMs were seeded in a medium culture dish at a cell count of 1 × 10⁶ cells per milliliter and cultivated overnight at 37 °C (12–18 h). Next, BMDMs were incubated with LPS for 4 h, collected, transferred to a test tube, treated with or without CTG12 for one hour and then stimulated with nigericin for 45 min. The samples were then subjected to centrifugation at 400 × g for 5 min, and the cell supernatant was discarded. The cells were then washed twice with Hank’s balanced salt solution (HBSS) and stained with 4 μM MitoSOX™ Red mitochondrial superoxide indicator (M36008, Invitrogen) at 37 °C for ten minutes. Subsequently, cells were rinsed twice with HBSS and re-suspending them in 200 uL HBSS. The obtained samples were then subjected to flow cytometric analysis using the FACSCanto™ II cell analyser.

#### Intracellular K^+^ measurement

To determine the intracellular K^+^ concentration, BMDMs were first seeded into 12-well cell culture plates overnight and then treated with 50 ng/mL LPS for four hours. The cells were first exposed to CTG12 for one hour, followed by ATP stimulation for 45 min. Following that, discarding the cell supernatant, followed by three cell washes with potassium-free buffer. This was followed by addition of ultrapure HNO_3_ to the cells for lysis and collection of the cell lysate. The samples were boiled at 105 °C for 30 min. The samples were then brought to room temperature by adding ddH₂O to a final volume of 5 ml, following which the levels of intracellular K^+^ in the samples were determined using inductively coupled plasma mass spectrometry (ICP-MS).

#### Intracellular Ca^2+^ assay

BMDMs were added to a 384-well plate at a concentration of 25,000 cells per milliliter, allowed to grow overnight, followed by LPS treatment for four hours before being stimulated with ATP for 45 min, with or without CTG12. ATP-triggered Ca^2^⁺ fluxes were recorded using the FLIPR Tetra® system (Molecular Devices, USA).

#### Cellular thermal shift assay (CETSA)

THP1 cells were incubated with CTG12 or DMSO for 1 h. Harvested cells were resuspended in PBS supplemented with protease inhibitors. Cell suspensions were subjected to three rapid freeze–thaw cycles using liquid nitrogen and a 37 °C water bath. The lysates were centrifuged at 12,000 × g for 10 min at 4 °C to remove cell debris. The supernatant (soluble protein fraction) was divided into 20 aliquots and heated individually at different temperatures (ranging from 37 °C to 67 °C) in a thermal cycler. After heating, samples were centrifuged again at 12,000 × g for 10 min at 4 °C. The supernatants were transferred to new tubes, mixed with SDS-PAGE loading buffer, denatured at 95 °C for 5 min, and analyzed by western blotting.

#### Immunoprecipitation and pull-down assays

The strep-Tag II-tagged plasmid (strep-Tag II-NLRP3), HA-tagged plasmid (HA-NEK7), and ASC plasmid were transfected into 293 T cells. After 24 h of expression, the cells were treated with CTG12 compound for 4 h. NP-40 lysis buffer (pH 7.4, 150 mM NaCl, 50 mM Tris, 2 mM EDTA, 0.5% Nonidet P-40) and protease inhibitor (Target Mol, C0001) were used to lyse cells, lysate was collected, centrifuged at 12,000 × g for 15 min at 4 °C, and the supernatant was incubated with affinity beads for 4 h. Samples were collected and resuspended in 1 × SDS-PAGE loading buffer, and the pulled-down target protein was examined using western blot.

#### LPS-induced acute systemic inflammation

Female C57BL/6 mice at 8 weeks of age were administered by intraperitoneal injection CTG12 (15 or 30 mg/kg), MCC950 (40 mg/kg), or CTG12 (15 mg/kg) in combination with MCC950 (40 mg/kg) (*n* = 6). One hour later, lipopolysaccharide (LPS) (20 mg/kg) was injected intraperitoneally. Five hours after LPS injection, the mice were sacrificed by cervical dislocation. Blood was collected via retro-orbital bleeding to isolate serum samples, and the peritoneal cavity was washed with 10 mL of ice-cold 1 × PBS to obtain peritoneal lavage fluid. The levels of IL-1β, TNF-α, IL-6, IL-18, and CXCL10 in the serum and peritoneal lavage fluid were measured by ELISA.

### Statistical analyses

The statistical analyses were performed as follows: comparisons between two groups were conducted using the unpaired Student's t-test, while comparisons among multiple groups were analyzed by one-way ANOVA followed by Dunnett’s or Sidak’s post hoc tests (GraphPad Prism, USA). Data are presented as mean ± SEM.

### Licochalcone B derivatives attenuate NLRP3 inflammasome activation in BMDMs.

**Fig. 1:**
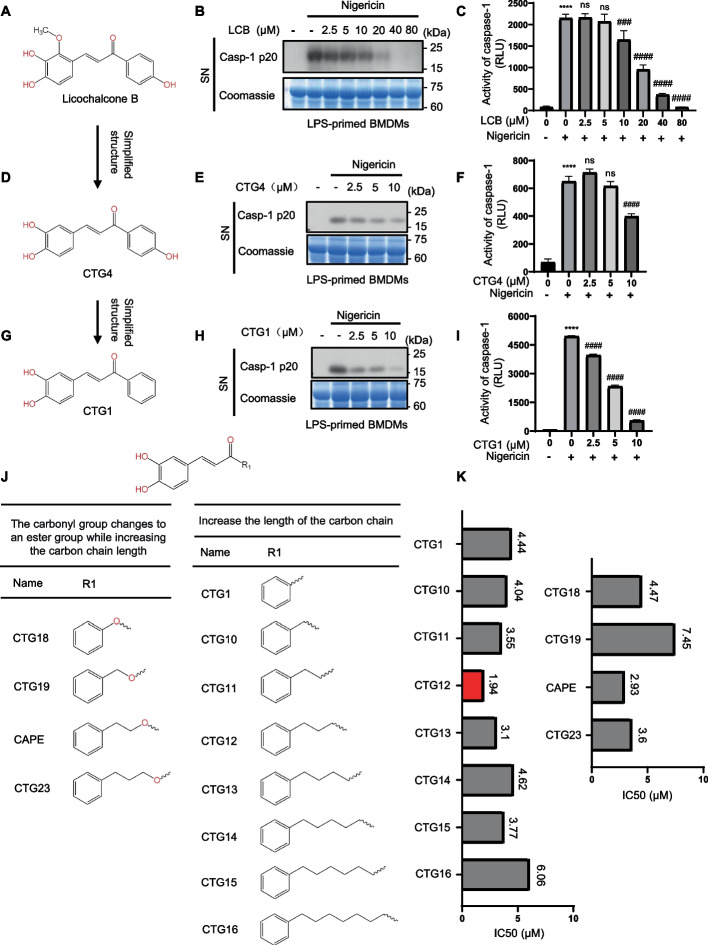
Licochalcone B derivatives inhibit NLRP3 inflammasome activation. **A** The structure of Licochalcone B. **B**-**C** BMDMs were primed with LPS for 4 h, treated with Licochalcone B for 30 min, and then stimulated with nigericin for 25 min. Western blot analysis of caspase-1 (p20) in culture supernatants (SN) (**B**) and supernatants were collected for the measurement of caspase-1(**C**). **D** The structure of CTG4. **E**–**F** BMDMs were primed with LPS for 4 h, treated with CTG4 for 30 min, and then stimulated with nigericin for 25 min. Western blot analysis of caspase-1 (p20) in culture supernatants (SN) (**E**), and supernatants were collected for the measurement of caspase-1 (**F**). **G** The structure of CTG1. **H**-**I** BMDMs were primed with LPS for 4 h, treated with CTG1 for 30 min, and then stimulated with nigericin for 25 min. Western blot analysis of caspase-1 (p20) in culture supernatants (SN) (H), and supernatants were collected for the measurement of caspase-1 (**I**). **J** The structure of formulas of Licochalcone B derivatives. (K) IC50 values of Licochalcone B derivatives. Coomassie blue–stained gels used as loading control. Data represent as mean ± SEM (*n* = 3). Compared to con, **** *p* < 0.0001; compared to a concentration of 0 μM, #### *p* < 0.0001 and ns: not significant (one-way ANOVA with Dunnett’s post-hoc test)

NLRP3 inflammasome activation plays a crucial role in the advancement of inflammatory diseases [19]. Following the activation of NLRP3, the NLRP3 inflammasome recruits ASC adaptor molecules to nucleate signalosome assembly and triggers the conversion of pro-caspase-1 to caspase-1 (caspase-1 p20) with catalytic activity, thereby facilitating the secretion of IL-1β [20, 21]. We evaluated the effect of licochalcone B (Fig. 1.A), on the NLRP3 inflammasome. Western blot and activity assay results revealed that licochalcone B could inhibit the activation of caspase-1 induced by nigericin, but complete inhibition required a concentration of 40 μM (Fig. 1.B-C), with a notably high IC50 value. First, we thought of simplifying the structure of licochalcone B. Next, by comparing licochalcone B with the previously studied Echinatin from our research group, we found that Echinatin (which lacks a hydroxyl group present in licochalcone B) was not increased in their effects (IC50 values) (Supplementary Fig. 1.A-B). Therefore, we proceeded to simplify licochalcone B by removing the methoxy group on its benzene ring, yielding the derivative CTG4 (Fig. 1.D). To assess the impact of CTG4 on the NLRP3 inflammasome, we stimulated the NLRP3 inflammasome in BMDMs with nigericin and observed through western blot and activity assays that CTG4 inhibited caspase-1 activation (Fig. 1.E–F). Its IC50 value was lower than that of licochalcone B (Supplementary Fig. 1.C). Subsequently, we further simplified the structure of CTG4 by removing the hydroxyl group, resulting in CTG1 (Fig. 1.G). The western blot and activity assay results demonstrated that CTG1 also inhibited caspase-1 activation (Fig. 1. H-I). Its IC50 value was lower than those of both licochalcone B and CTG4 (Supplementary Fig. 1.D), indicating the effectiveness of our structural modifications. To identify the licochalcone B derivative with the lowest IC50 value and the best biological activity, we introduced one, two, three, four, five, six, and seven carbon–carbon single to bond CTG1, generating seven compounds: CTG10, CTG11, CTG12, CTG13, CTG14, CTG15, and CTG16 (Fig. 1. J). Activity assays revealed that the seven compounds inhibited caspase-1 activation in BMDMs stimulated by nigericin (Supplementary Fig. 1.E-K). Calculation of their IC50 values showed that CTG12 exhibited the most significant biological effect (Fig. 1. K). The structure of CTG12 resembled us of caffeic acid phenethyl ester (CAPE), with the key difference being that CAPE replaces the carbon–carbon single bond with an ester bond. Previous studies have reported CAPE's anti-inflammatory effects. Therefore, we obtained CTG18, CTG19, CAPE and CTG23 by introducing ester bonds to further modify CTG10, CTG11, CTG12 and CTG13 to determine whether this would enhance biological activity (Fig. 1. J). Activity assays showed that the ester-modified compounds also inhibited caspase-1 activation in nigericin-stimulated BMDMs (Supplementary Fig. 1.L-O). However, their IC50 values were higher than those of the unmodified counterparts. In summary, through a series of structural modifications, we identified CTG12 as the compound with the most significant biological activity. Therefore, we conducted in-depth follow-up studies on CTG12.

### CTG12 dose-dependently inhibits NLRP3 inflammasome activation in BMDMs and THP1

**Fig. 2:**
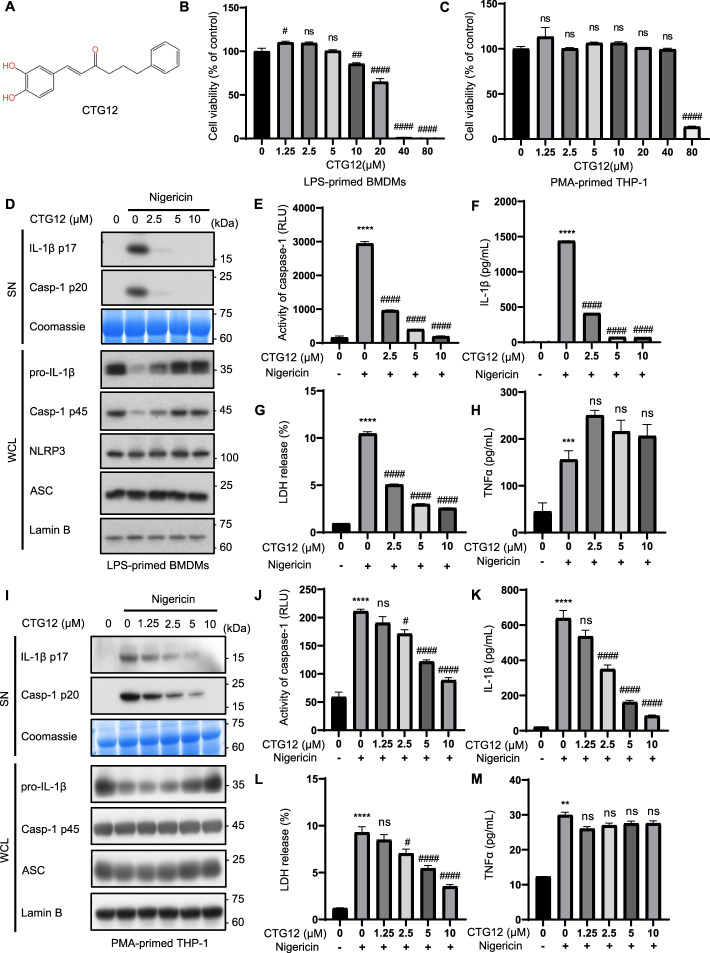
CTG12 inhibits NLRP3 inflammasome activation in mouse BMDMs and human THP1. **A** The structure of CTG12. **B**, **C** Cell viability of BMDMs (**B**) and THP1 cells (**C**) treated with CTG12. (D-H) Western blot analysis of IL-1β (p17), caspase-1 (p20) in culture supernatants (SN) and pro-IL-1β, caspase-1 (p45), NLRP3, ASC in whole cell lysates (WCL) of LPS-primed BMDMs treated with CTG12 and then stimulated with Nigericin (25 min) (**D**), supernatants were collected for the measurement of caspase-1(**E**), IL-1β (**F**), LDH (**G**) and TNF-α (**H**). (I-M) THP-1 cells primed with PMA for 12 h were treated with CTG12 for 1 h and then stimulated with nigericin for 55 min. Western blot analysis of IL-1β (p17), caspase-1 (p20) in culture supernatants (SN) and pro-IL-1β, caspase-1 (p45), ASC in whole cell lysates (WCL) of THP-1 (**I**), supernatants were collected for the measurement of caspase-1(**J**), IL-1β (**K**), LDH (**L**) and TNF-α (**M**). Coomassie blue-stained gels was used as loading control and Lamin B was used as a control for equal loading of the samples. Data represent as mean ± SEM (*n* = 3). Compared to con, ** *p* < 0.01, **** *p* < 0.0001; compared to a concentration of 0 μM, # *p* < 0.05, ## *p* < 0.01, #### *p* < 0.0001 and ns: not significant (one-way ANOVA with Dunnett’s post-hoc test)

We first performed cell viability assays to evaluate the cytotoxicity of CTG12 in mouse BMDMs and human THP1 cells. Based on the assessments of cell viability, CTG12 (Fig. 2. A) had no cytotoxic effects at concentrations below 10 μM in BMDMs and did not exhibit any cytotoxicity at doses below 40 μM in THP1 (Fig. 2. B-C). Subsequently, we evaluated the effect of CTG12 (0–10 μM) on nigericin-induced NLRP3 inflammasome activation. We further tested the activation of caspase-1, the secretion of IL-1β, and the release of LDH in CTG12 under Nigericin stimulation. Through western blot and activity measurement methods, the results indicated that CTG12 dose-dependently inhibited the activation of caspase-1, the secretion of IL-1β, and the release of LDH, but did not inhibit the inflammasome-independent cytokine TNF-α secretion induced by nigericin in BMDMs (Fig. 2. D-H). Notably, CTG12 inhibited the activation of NLRP3 inflammasome at a concentration of 2.5 μM, showing a tenfold increase in efficacy compared to our previous research on licochalcone B.

Considering that some compounds cannot be used in clinical practice due to species differences. Subsequent investigations delineated the modulatory effects of CTG12 compounds derivatives on NLRP3 inflammasome dynamics in phorbol-differentiated THP1 macrophages. Our findings demonstrated that CTG12 suppressed caspase-1 activation and IL-1β release triggered by nigericin in a dose-dependent manner in PMA-treated THP1 cells and they were completely inhibited at a dose of 10 μM (Fig. 2. I). We also measured caspase-1 activation, IL-1β secretion, and LDH release. The results demonstrated that CTG12 exhibited a dose-dependent inhibition of caspase-1 activation, IL-1β secretion and LDH release but did not inhibit the inflammasome-independent cytokine TNF-α secretion in PMA-treated THP1 cells and the inhibition effect had already appeared at 2.5 μM (Fig. 2. J-M). Taken together, the efficacy of CTG12 in NLRP3 inflammasome inhibition was manifested through caspase-1 proteolytic activity and LDH release in human THP1 cells. Therefore, CTG12 inhibited NLRP3 inflammasome activation in nigericin-stimulated BMDMs and THP1 cells and there was no species difference. In addition, we also evaluated the effects of CTG11 and CTG13, compounds similar to CTG12. Through western blot and activity measurement methods, the results indicated that CTG11 and CTG13 dose-dependently inhibited the activation of caspase-1, the secretion of IL-1β, and the release of LDH but did not inhibit the inflammasome-independent cytokine TNF-α secretion induced by nigericin in BMDMs (Supplementary Fig. 2). But their effectiveness is weaker than that of CTG12 consistent with the IC50 detection results.

### CTG12 specifically inhibits canonical and noncanonical NLRP3 inflammasomes activation

**Fig. 3:**
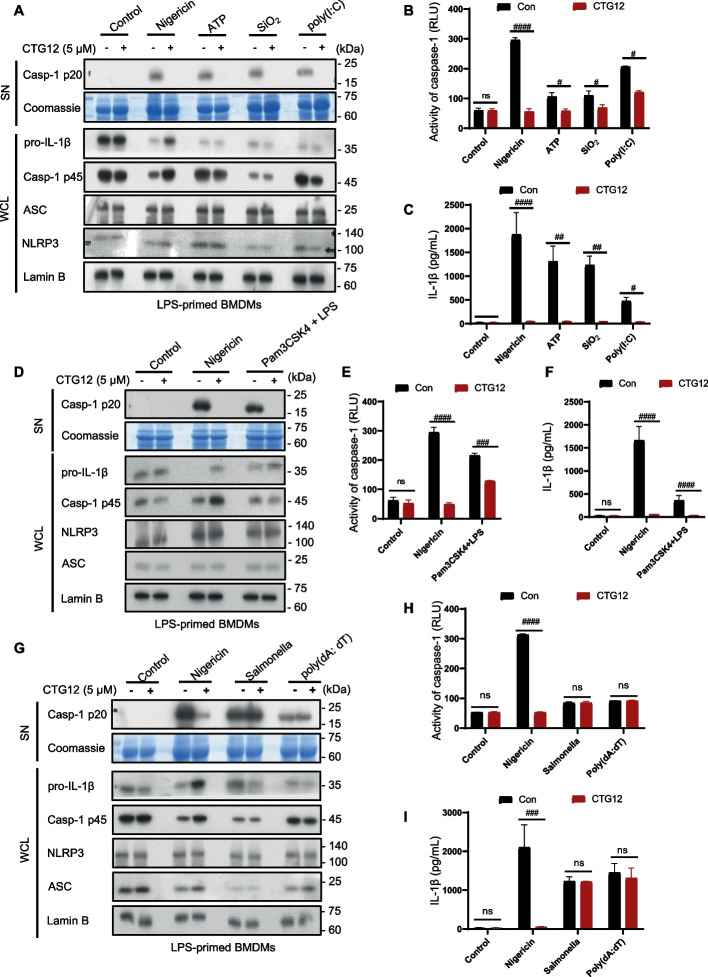
CTG12 specifically inhibits canonical and noncanonical NLRP3 inflammasome activation. **A**-**C** BMDMs were primed with LPS and then treated with CTG12 (5 μM), then stimulated with Nigericin, ATP, poly (I:C), or SiO₂. Western blot analysis of caspase-1 (p20) in culture supernatants (SN) and pro-IL-1β, pro-caspase-1 (p45), NLRP3 and ASC in whole-cell lysates (WCL); secretion of activated caspase-1 (p20) in the culture supernatant (SN) of BMDMs (A), supernatants were collected for the measurement of caspase-1 (**B**) and IL-1β (**C**). **D**-**F** BMDMs primed with Pam3CSK4 treated with CTG12 (5 μM), followed by cytosolic LPS. Western blot analysis of caspase-1 (p20) in culture supernatants (SN) and pro-IL-1β, pro-caspase-1 (p45), NLRP3 and ASC in whole-cell lysates (WCL); secretion of activated caspase-1 (p20) in the culture supernatant (SN) of BMDMs (**D**), supernatants were collected for the measurement of caspase-1 (**E**) and IL-1β (**F**). (**G**-**I**) BMDMs were primed with LPS treated with CTG12 (5 μM), then stimulated with Nigericin, Salmonella, poly (dA:dT). Western blot analysis of caspase-1 (p20) in culture supernatants (SN) and pro-IL-1β, pro-caspase-1 (p45), NLRP3 and ASC in whole-cell lysates (WCL); secretion of activated caspase-1 (p20) in the culture supernatant (SN) of BMDMs (**G**), supernatants were collected for the measurement of caspase-1 (**H**) and IL-1β (**I**). Coomassie blue-stained gels used as loading control and Lamin B used as a control for equal loading of the samples. Data represent as mean ± SEM (*n* = 3). # *p* < 0.05, ## *p* < 0.01, ###*p* < 0.001, #### *p* < 0.0001 and ns: not significant (one-way ANOVA with Dunnett’s post-hoc test)

NLRP3 inflammasome is a multiprotein complex [22], and the activation of NLRP3 is able to be induced by various stimuli [23], including classical and non-classical stimuli-induced activation of NLRP3 inflammasome. Next, we examined the influence of CTG12 on NLRP3 inflammasomes activation triggered by other stimuli. After treating LPS-primed BMDMs with CTG12, we used classical NLRP3 inflammasome stimuli such as ATP, SiO_2_, or poly(I:C) to treat them. Our results showed that CTG12 potently inhibited caspase-1 activation and IL-1β secretion triggered by classical NLRP3 stimulation (Fig. 3. A-C), and did not affect the inflammasome-independent cytokine TNF-α (Supplementary Fig. 3.A). Caspase-11 is activated by intracellular LPS or Gram-negative bacteria, leading to noncanonical NLRP3 inflammasome activation [24]. We then investigated how CTG12 affects the non-canonical pathway [25]. The findings demonstrated that CTG12 suppressed cytosolic LPS-induced caspase-1 activation and IL-1β release in BMDMs primed with Pam3CSK4 (Fig. 3. D-F) and did not affect the inflammasome-independent cytokine TNF-α (Supplementary Fig. 3.B). Our findings indicate that the CTG12 compound is a potent broad-spectrum inhibitor of NLRP3 inflammasome activation, effectively blocking both classical and non-classical pathways.

Besides NLRP3 inflammasome, other inflammasomes exist, including AIM2 inflammasome (induced by poly (dA:dT)) and NLRC4 inflammasome (triggered by Salmonella Typhimurium) [26, 27]. We then assessed whether CTG12 influences the process of activating AIM2 and NLRC4. The findings demonstrated that CTG12 had no inhibitory effect on caspase-1 induction and IL-1β secretion triggered by NLRC4 and AIM2 activation in BMDMs (Fig. 3. G-I), and did not affect the inflammasome-independent cytokine TNF-α (Supplementary Fig. 3. C). The data demonstrated that CTG12 acted specifically on the NLRP3 and did not impact AIM2 or NLRC4.

Additionally, poly(I:C) not only activates the NLRP3 inflammasome but also serves as a classic agonist of the RIG-I-MAVS pathway [28]. We further investigated the effect of CTG12 on the RIG-I-MAVS signaling pathway. Our results demonstrated that CTG12 treatment did not alter poly(I:C)-induced IRF3 phosphorylation (Supplementary Fig. 3. D). Consistently, CTG12 showed no significant inhibitory effect on the mRNA expression levels of interferon-related genes or inflammatory genes triggered by poly(I:C) (Supplementary Fig. 3. E–G). Collectively, these findings indicated that CTG12 specifically suppresses the NLRP3 inflammasome signaling pathway without affecting the RIG-I-MAVS signaling pathway.

### CTG12 impedes NLRP3-dependent ASC oligomerization in NLRP3 inflammasome activation

**Fig. 4:**
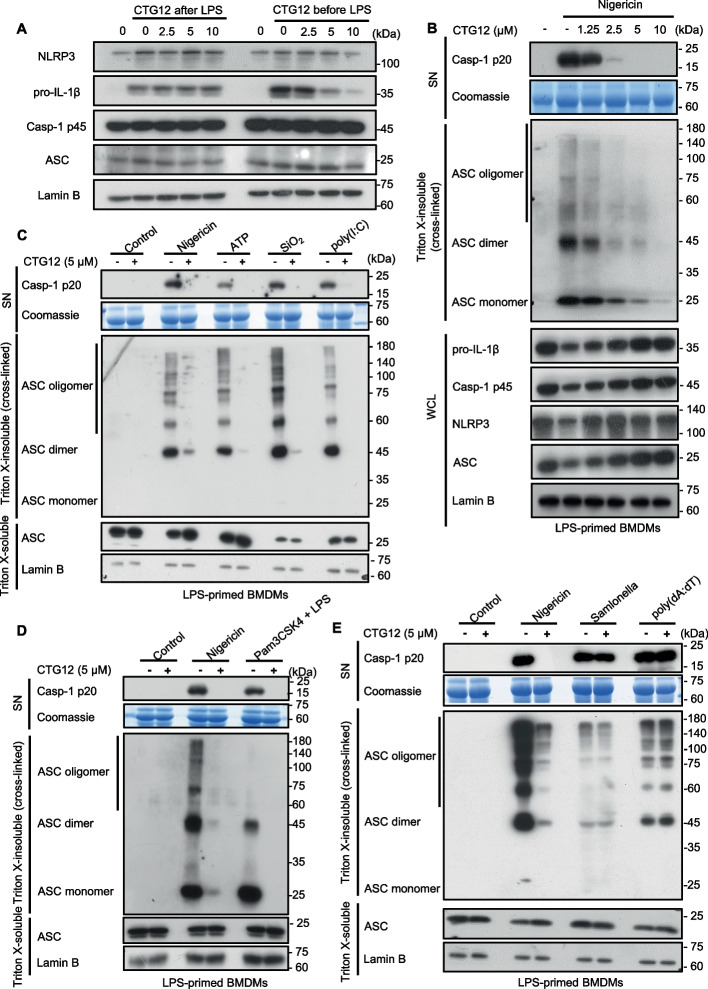
CTG12 inhibits LPS-induced priming step of inflammasome activation and specifically inhibits canonical and noncanonical NLRP3 inflammasome activation by blocking NLRP3-dependent ASC oligomerization. **A** BMDMs were cultured with LPS for 4 h, then incubated with CTG12 for 1 h, or BMDMs were first treated with CTG12 for 1 h, followed by stimulation with LPS for 4 h, western blot analysis of protein levels of NLRP3, ASC, pro-IL-1β, Cas-1 p45, Lamin B. **B** BMDMs were primed with LPS treated with CTG12 (5 μM), then stimulated with Nigericin, western blot analysis of Triton X-insoluble microparticle cross-linking ASC. **C** BMDMs were primed with LPS treated with CTG12 (5 μM), then stimulated with Nigericin, ATP, poly(I:C), SiO_2_, western blot analysis of in Triton X insoluble microparticle cross-linked ASC. **D** BMDMs were primed with Pam3CSK4 treated with CTG12 (5 μM), followed by stimulation with ultra-LPS, western blot analysis of Triton X-insoluble microparticle cross-linking ASC. **E** BMDMs were primed with LPS treated with CTG12 (5 μM), then stimulated with Nigericin, salmonella or poly (dA:dT), western blot analysis of in Triton X insoluble microparticle cross-linked ASC. Coomassie blue-stained gels used as loading control and Lamin B used as a control for equal loading of the samples

Previous studies have shown that lipopolysaccharide initiates canonical NF-κB signaling in macrophages, establishing the transcriptional priming phase which is essential for transcriptional upregulation of NLRP3 and IL-1β [29–31]. To examine whether CTG12 could abrogate NLRP3 activation through suppressing the key inflammasome components in the priming step, we measured the expression of NLRP3, pro-IL-1β, pro-caspase-1, ASC proteins and the production of IL-6, TNF-α. When BMDMs were administered with CTG12 before LPS stimulation, we observed that pretreatment with CTG12 restrained the expression of NLRP3, pro-IL-1β and the production of IL-6 and TNF-α, indicating that CTG12 inhibited NF-κB activation (Signal 1) following LPS priming. In contrast, administering CTG12 after LPS made no difference to the expression of NLRP3, pro-IL-1β and the production of IL-6 and TNF-α (Fig. 4. A, Supplementary Fig. 3. H-I). These results suggest that, in our experimental context where LPS treatment precedes drug administration, CTG12 inhibits the assembly and activation of the NLRP3 inflammasome not by reducing proteins already expressed induced by NF-κB pathway activation.

Next, we investigated the mechanisms by which CTG12 inhibits NLRP3 activation. Upon stimulation by an activator, oligomerization occurs in ASC, which is a critical step in NLRP3 inflammasome activation circuitry for NLRP3 activation and caspase-1 cleavage [32]. Thus, we investigated the impact of CTG12 on ASC oligomerization stimulated by multiple NLRP3 activators. We found that CTG12 dose-dependently inhibited nigericin-induced ASC oligomerization (Fig. 4. B). We also found that CTG12 uniquely blocks ASC oligomerization induced by nigericin, ATP, SiO₂, poly(I:C), and cytosolic LPS (Fig. 4. C-D). However, CTG12 had no impact on ASC aggregation during AIM2 or NLRC4 activation (Fig. 4. E). In conclusion, our results showed that CTG12 may specifically inhibit NLRP3 activation by suppressing upstream events that hinder the oligomerization of ASC.

### CTG12 does not influence K^+^ efflux, Ca^2+^ flux, and mtROS generation, but blocks NLRP3 activation by disrupting the NLRP3-ASC linkage not by directly binding NLRP3 or ASC

**Fig. 5:**
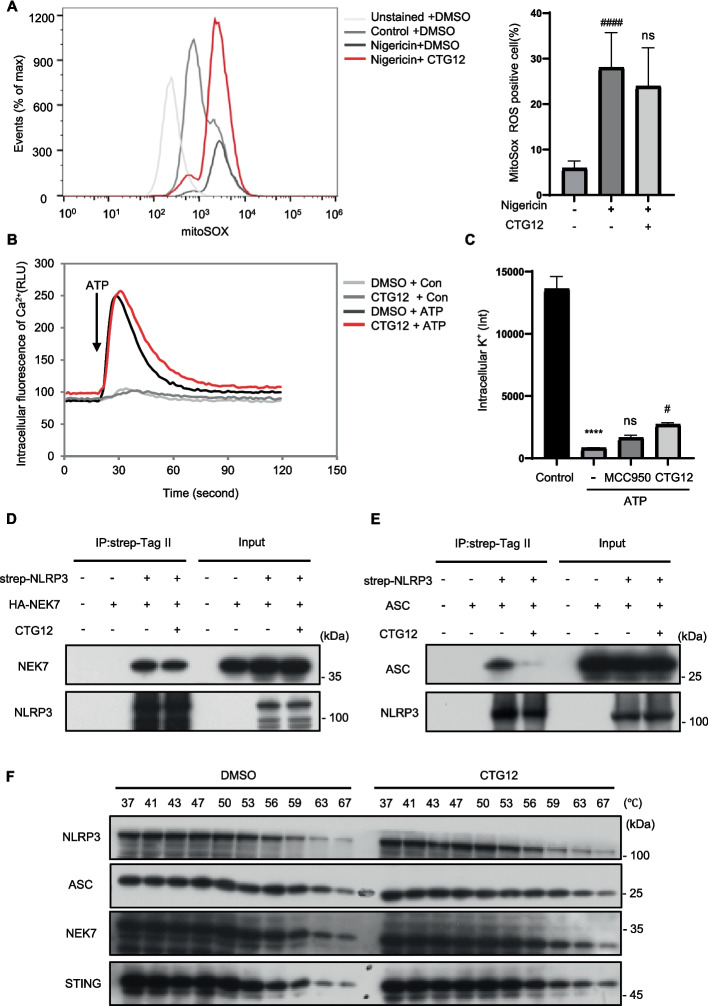
CTG12 has no effect on K^+^ efflux, Ca^2+^ signaling, mitochondrial reactive oxygen species (mtROS) production and not directly binds NLRP3 or ASC, but suppresses NLRP3 activation by disrupting the NLRP3-ASC interaction. **A** Percentage of mtROS-producing in LPS-primed BMDMs treated with CTG12 for one hour and then used nigericin to stimulate for 45 min, followed by staining with MitoSOX™. **B** The FLIPR Tetra® system to detect intracellular Ca^2+^ fluorescence intensity in LPS-primed BMDMs. **C** Inductively coupled plasma atomic emission light (ICP-AES) to detect the intensity of K.^+^ in BMDM cells. **D** The 293 T cells were transfected with strep-Tag II-NLRP3, HA-NEK7 and then treated with CTG12. Co-immunoprecipitation was performed with anti-strep agarose beads. **E** The 293 T cells were transfected with strep-Tag II-NLRP3, ASC and then treated with CTG12. Co-immunoprecipitation was performed with anti- strep agarose beads. **F** THP1 cells were harvested and subjected to at least three freeze–thaw cycles. CTG12 was then added to the resulting supernatant and the mixture was incubated at room temperature for 30 min. Following incubation, samples were heated for 3 min at different temperatures, centrifuged, and the supernatants were collected for western blot analysis. Data represent as mean ± SEM (*n* = 3). Compared to con, **** *p* < 0.0001; compared to a concentration of 0 μM, # *p* < 0.05, #### *p* < 0.0001 and ns: not significant (one-way ANOVA with Dunnett’s post-hoc test)

Since mtROS production is known to be crucial upstream regulator of ASC oligomerization [33], we performed experiments to examine whether CTG12 influences mtROS production. Our results indicated that nigericin treatment markedly increased mtROS production. However, when CTG12 was added, no significant alteration in mtROS levels was detected (Fig. 5. A). Thus, we concluded that CTG12 does not influence mtROS production when NLRP3 inflammasome is activated. We further studied the effect of CTG12 on Ca^2+^ flux during NLRP3 activation. We employed ATP to stimulate LPS-induced BMDMs, there was a significant increase in the Ca^2+^ flux, but CTG12 treatment did not inhibit it (Fig. 5. B). To further clarify the mechanism underlying the impact of CTG12 on the NLRP3 activation, we further profiled CTG12's unique ability on K^+^ efflux to intercept NLRP3 signaling nodes upstream [34, 35]. When BMDMs were stimulated with ATP, intracellular potassium levels dropped sharply, compared to MCC950 positive control, there was a slight increase in CTG12 treatmen (Fig. 5. C), but the modest inhibitory effect of CTG12 on K⁺ efflux was not significant enough to completely inhibit the NLRP3 inflammasome. Collectively, these findings indicate that CTG12 suppressed NLRP3 activation without the involvement of K⁺ efflux, Ca^2^⁺ flux, and mtROS generation.

The assembly of NLRP3 inflammasome is also crucial for its activation. Thus, we investigated whether CTG12 affects the assembly process of NLRP3 inflammasome, with a focus on its impact on the protein–protein interactions involved in the assembly. Firstly, we examined the effect of CTG12 on the NLRP3-NEK7 and NLRP3-ASC interactions [36]. We conducted exogenous Co-IP assays by transfecting 293 T cells with Strep-Tag II-NLRP3, HA-NEK7, and ASC plasmids. Our findings demonstrated that NLRP3-NEK7 interaction remained unaffected with CTG12 treatment (Fig. 5. D). Subsequently, we examined the binding between ASC and NLRP3, a critical step for NLRP3 oligomerization in NLRP3 activation. We discovered that CTG12 significantly inhibited the association between ASC and NLRP3 (Fig. 5. E).

Next, to further determine the mechanism, we verified whether CTG12 can directly bind to the NLRP3 and ASC proteins. CETSA (Cellular Thermal Shift Assay) evaluates changes in protein thermal stability to detect protein-small molecule interactions. Typically, drug binding stabilizes the target protein, resulting in less degradation at higher temperatures compared to the unbound protein. Therefore, we further assessed the affinity among NLRP3, ASC proteins, and CTG12 using CETSA. The results demonstrated that CTG12 did not affect the thermal stability of NLRP3 or ASC (Fig. 5. F). The CETSA assay confirmed that CTG12 does not directly target NLRP3 or ASC, thereby reducing the activation of the NLRP3 inflammasome. These results profiling revealed CTG12 disrupts the NLRP3-ASC interaction but not affecting NLRP3 interaction with NEK7. These findings indicate that CTG12 disrupts NLRP3 assembly by interfering with the ASC-NLRP3 interaction.

### CTG12 alleviates LPS-induced acute systemic inflammation in mice

**Fig. 6:**
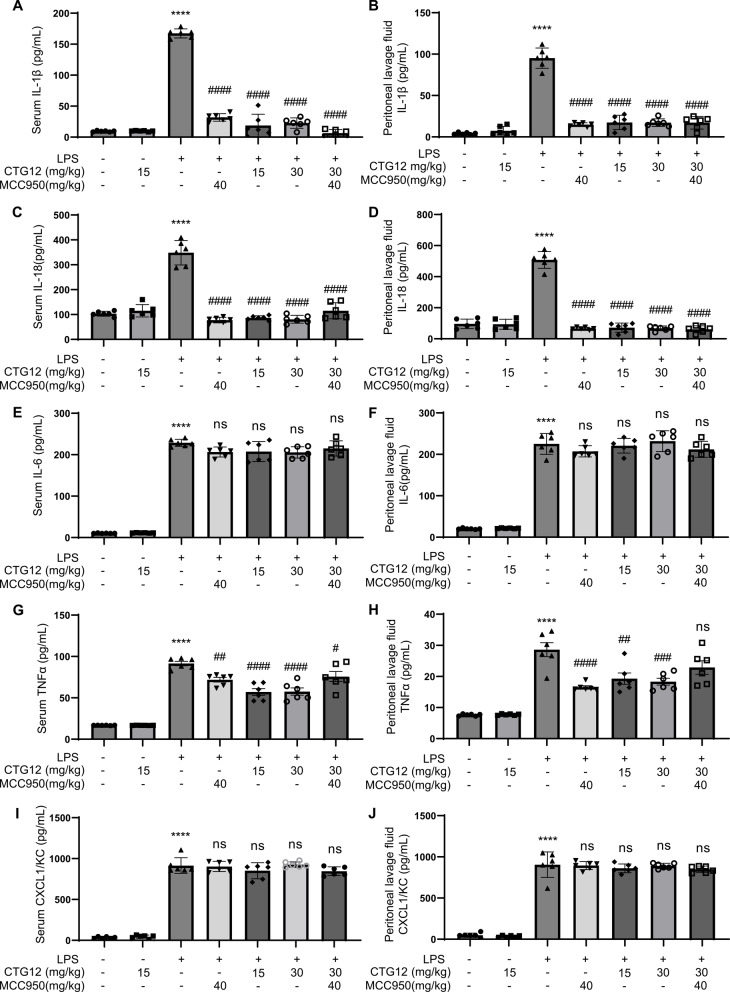
CTG12 alleviates LPS-induced acute systemic inflammation in mice. **A**-**J** Female mice (8 weeks) were injected intraperitoneally with CTG12 (0 mg/kg, 15 mg/kg, 30 mg/kg), MCC950 (40 mg/kg) or CTG12 (30 mg/kg) + MCC950 (40 mg/kg) for 1 h, followed by an injection of 20 mg/kg LPS for 5 h (*n* = 6). The levels of IL-1β (A, B), IL-18 (**C**, **D**), IL-6 (**E**, **F**), TNF-α (**G**, **H**) and CXCL1/KC (**I**, **J**) in peritoneal lavage fluid and serum were analyzed by ELISA (*n* = 6). Data represent as mean ± SD. Compared to con, **** *p* < 0.0001; compared to a concentration of 0 μM, # *p* < 0.05, ## *p* < 0.01, ###*p* < 0.001, #### *p* < 0.0001 and ns: not significant (one-way ANOVA with Dunnett’s post-hoc test)

Intraperitoneal injection in an LPS-induced acute systemic inflammation mouse model is a widely used approach for studying the mitigation of inflammatory diseases. Using LPS-activated NLRP3 inflammasome model, we explored the therapeutic efficacy of CTG12 in treating LPS-induced acute systemic inflammation. The results demonstrated that in control group mice, the NLRP3 inflammasome mediated the production of IL-1β and IL-18 after 5 h of LPS treatment. However, pharmacological intervention with CTG12 significantly decreased the formation of IL-1β and IL-18 in serum and peritoneal lavage fluid. Notably, CTG12 showed the same effect or even outperformed at 15 mg/kg as the positive control drug MCC950(40 mg/kg) (Fig. 6. A-D). In contrast, the inhibition of IL-1β and IL-18 release by CTG12 was significantly higher than its inhibition of the inflammasome-independent cytokine TNF-α, while IL-6 and CXCL1/KC remained unaffected by CTG12 treatment (Fig. 6. E-J). These findings indicate that CTG12 effectively suppresses NLRP3 inflammasome activation in vivo and mitigates LPS-induced inflammatory responses in mice.

## Discussion

NLRP3 inflammasome has been a key research object in the field of immune inflammatory response [37], and plays a crucial role on human health, mediating the pathogenesis of various diseases [38]. However, overactivation of NLRP3 is linked to the onset and advancement of several disorders, including acute systemic inflammation, gout, and neurodegenerative diseases [39]. Numerous NLRP3 inflammasome inhibitors have been discovered, and these molecules exhibit potent inhibitory effects on NLRP3 inflammasomes [40]. However, no small-molecule NLRP3 inhibitors have been authorized for clinical use because of their limited efficacy or significant toxicity. For example, MCC950 is a highly specific, potent small-molecule inhibitor that was identified as an NLRP3 inhibitor, but pfizer announces discontinuation of phase 2 clinical trial evaluating MCC950 due to its hepatotoxicity [41]. Therefore, it is necessary to discover secure and efficient inhibitors of NLRP3.

Licorice has been used for centuries in traditional medicine because of its ability to relieve coughs and reduce inflammation. It is one of the most significant herbs and a central area of study in traditional Chinese medicine [42]. Our group has previously studied licochalcone B and Echinatin in licorice are potential and effective NLRP3 inflammasome inhibitors [14, 15]. However, they exerted a complete inhibitory effect at a concentration of 40 μM, and exhibited relatively low potency (high IC50). In addition, Echinatin did not specifically inhibit NLRP3 activation. Research has indicated that it is essential to optimize the structure of natural bioactive ingredients during the drug development phase [43]. After structural modification in these compounds, due to their increased effectiveness, better binding affinity, and reduced side effects, they may be used by society as important therapeutic agents. Therefore, we structurally engineered licochalcone B to discover NLRP3 inflammasome inhibitors with lower IC50 values and higher specificity.

First, we evaluated the effects of licochalcone B and Echinatin on the NLRP3 inflammasome and found that the removal of the hydroxyl group on the benzene ring of Echinatin does not increased their effects (IC50 values). Therefore, we considered simplifying licochalcone B by removing the methoxy group on its benzene ring, resulting in licochalcone B derivative CTG4. We observed that the IC50 value of CTG4 was lower than that of licochalcone B, suggesting that our structural simplification of licochalcone B was effective. Consequently, we further simplified the structure of CTG4 by removing the hydroxyl group to create CTG1. The results showed that the IC50 value of CTG1 was lower than that of licochalcone B and CTG4, possibly because the phenolic hydroxyl group becomes a benzene ring, enhancing the structural stability of the compound. This outcome supported the strategy of our structural modifications. Next, to obtain the licochalcone B derivative with the lowest IC50 value and the best biological effect on inhibiting the activation of the NLRP3 inflammasome, we introduced one, two, three, four, five, six, and seven carbon–carbon single bonds to CTG1, yielding seven compounds: CTG10, CTG11, CTG12, CTG13, CTG14, CTG15, and CTG16. We then evaluated the effects of these licochalcone B derivatives on the NLRP3 inflammasome and found that all of them could inhibit the activation of caspase-1 in the supernatant of BMDMs stimulated by nigericin. Among them, CTG12 exhibited the most significant biological effect. The IC50 results suggested that in structural modifications, either too short or too long carbon chains linking the benzene rings would reduce the compound's efficacy, possibly due to structural instability caused by extreme chain lengths. The structure of CTG12 reminded us of caffeic acid phenethyl ester (CAPE), with the key difference being that CAPE replaces the carbon–carbon single bond with an ester bond. Previous studies have shown that CAPE possesses anti-inflammatory properties. Therefore, we further modified CTG10, CTG11, CTG12, and CTG13 by introducing ester bonds to investigate whether the modifications would improve their biological effects. The activity assay results revealed that the ester-modified compounds could still inhibit caspase-1 activation in BMDMs stimulated by nigericin. However, the calculated IC50 values were higher than those of the pre-modified compounds. This suggests that replacing the carbonyl group with an ester bond reduced the compounds' efficacy, possibly due to the instability of the ester bond, which is prone to hydrolysis. In conclusion, after a series of modifications, we found that CTG12 compound exhibited the most significant biological effect. Next, we assess the impact of CTG12 on NLRP3 inflammasomes from multiple angles. CTG12 strongly suppresses caspase-1 activation, IL-1β secretion, and LDH release in mouse BMDMs and human THP1 cells following NLRP3 activation. In addition, we examined the blocking effect of CTG12 on NLRP3 inflammasomes using different stimuli. CTG12 exhibits significant inhibition of multiple trigger-induced NLRP3 activation. Moreover, we found that CTG12 specifically inhibited classical and non-classical activation without affecting NLRC4 or AIM2 activation and the RIG-I-MAVS signaling pathway. These results suggest that CTG12 is a broad-spectrum NLRP3 inhibitor, and demonstrates a degree of specificity. However, our current study cannot entirely rule out potential impacts of CTG12 on other innate immune pathways or inflammasome-independent mechanisms. These results suggest that CTG12 is a relatively specific NLRP3 inflammasome inhibitor.

Next, we further explored the mechanism of CTG12 in inhibiting NLRP3 inflammasome activation. Our investigation revealed that CTG12 suppressed NLRP3-mediated ASC oligomerization, without influencing K^+^ efflux, Ca^2+^ influx, and mtROS production. In addition, we observed that CTG12 inhibited the interaction of ASC-NLRP3, while according to the CETSA, CTG12 did not target the NLRP3 or ASC directly. Therefore, we speculated that CTG12 functionally disrupts the NLRP3-ASC interaction, likely through indirect modulation of conformational dynamics or transient binding. However, the precise mechanism that CTG12 blocks the ASC-NLRP3 interaction remains uncovered and the specific underlying mechanisms require further exploration. To summarize, our findings illustrate that CTG12 is specific in inhibiting the assembly and activation stages of NLRP3, and it does not inhibit the entire process of NLRP3 activation, but blocks the NLRP3-ASC interaction to inhibit NLRP3 activation. In conclusion, it was discovered that CTG12 weakened ASC-NLRP3 interaction and effectively inhibited the formation of NLRP3 complexes and their downstream signal activation, such as the formation of IL-1β and IL-18.

LPS, which comes from gram-negative bacteria, is among the most powerful natural immunostimulants [44]. Moreover, it plays a vital role in the innate immune response during acute systemic inflammation. In our study, we used C57BL/6 J mice as a model, intraperitoneal injection of LPS resulted in NLRP3 inflammasome-dependent IL-1β and IL-18 generation and inflammation in mice, while intraperitoneal injection of CTG12 significantly inhibited LPS-mediated production of serum and intraperitoneal IL-1β and IL-18, and had a slight inhibitory effect on the inflammasome-independent cytokine TNF-α. In the animal experiments, injection of LPS simultaneously activated both LPS-induced the NF-κB signaling pathway and inflammasome pathway [45]. The TNF-α expression level measured as an endpoint is actually the result of the combined effects of LPS-induced the NF-κB signaling pathway and inflammasome pathways. Therefore, the modest inhibition of TNF-α in vivo may be a part of explained by CTG12 inhibition of NF-κB signaling pathway. However, the inhibitory activity of CTG12 on the inflammatory cytokines IL-1β and IL-18 is significantly stronger than its effect on the inflammasome-independent cytokine TNF-α. Collectively, our findings suggest that CTG12 can inhibit the NLRP3 activation in vivo and effectively treat NLRP3-related inflammation.

## Conclusion

In summary, we found that licochalcone B derivative CTG12 inhibits the activation of NLRP3 inflammasome in a dose-dependent manner, and its inhibitory effect is broad-spectrum. The mechanism is that CTG12 influences NLRP3 assembly by interfering with the ASC-NLRP3 interaction, thereby inhibiting NLRP3-dependent ASC oligomerization process in NLRP3 inflammasome activation. Meanwhile, in vivo, CTG12 exhibits anti-inflammatory activity in NLRP3-related inflammatory conditions, such as LPS-induced acute systemic inflammation. These findings demonstrate that CTG12 is a specific NLRP3 inflammasome inhibitor. Thus, CTG12 may be a potential therapeutic agent for diseases caused by overactivation of NLRP3.

## Supplementary Information

Supplementary Material 1. Supplementary Figure 1. Licochalcone B derivatives inhibit NLRP3 inflammasome activation.BMDMs were primed with LPS for 4 hours, treated with Echinatin, Licorice Chalcone B, CTG4, CTG1, CTG10, CTG11, CTG12, CTG13, CTG14, CTG15, CTG16, CTG18, CTG19, CAPE, CTG23for 30 minutes, and then stimulated with nigericin for 25 minutes. Supernatants were collected for the measurement of caspase-1. Data represent as mean ± SEM. Compared to con, **** *p* < 0.0001; compared to a concentration of 0 μM, ###*p* < 0.001, #### *p* < 0.0001 and ns:not significant. Supplementary Figure 2. CTG11 and CTG13 inhibit NLRP3 inflammasome activation in mouse BMDMs.The structure of CTG11.Western blot analysis of IL-1β, caspase-1in culture supernatantsand pro-IL-1β, caspase-1, NLRP3, ASC in whole cell lysatesof LPS-primed BMDMs treated with CTG11 and then stimulated with Nigericin, supernatants were collected for the measurement of caspase-1, IL-1β, LDHand TNF-α.The structure of CTG13.Western blot analysis of IL-1β, caspase-1in culture supernatantsand pro-IL-1β, caspase-1, NLRP3, ASC in whole cell lysatesof LPS-primed BMDMs treated with CTG13 and then stimulated with Nigericin, supernatants were collected for the measurement of caspase-1, IL-1β, LDHand TNF-α. Coomassie blue–stained gels used as loading control and Lamin B used as a control for equal loading of the samples. Data represent as mean ± SEM. Compared to con, ** *p* < 0.01, ***p < 0.001, **** *p* < 0.0001; compared to a concentration of 0 μM, ###*p* < 0.001, ####*p* < 0.0001 and ns: not significant. Supplementary Figure 3. CTG12 impedes the priming process of NLRP3 inflammasome activation and specifically inhibits canonical and noncanonical NLRP3 inflammasome activation.BMDMs were primed with LPS treated with CTG12, then stimulated with Nigericin ATP, poly, or SiO₂. Supernatants were collected for the measurement of TNF-α, BMDMs primed with Pam3CSK4 treated with CTG12, followed by cytosolic LPS. Supernatants were collected for the measurement of TNF-α, BMDMs were primed with LPS treated with CTG12, then stimulated with Nigericin, salmonella, poly. Supernatants were collected for the measurement of TNF-α.THP1 were pretreated for 1 h with various concentrations of CTG12 and then transfected with poly. P-IRF3, STING, IRF3, and Lamin B were analyzed by western blotting 2 h after polytransfection. The expression of IFN-β, TNF-α, CXCL10mRNA was detected by qPCR assay 4 h after polytransfection.BMDMs were cultured with LPS for 4 h, then incubated with CTG12 for 1 h, or BMDMs were first treated with CTG12 for 1 h, followed by stimulating with LPS for 4 h, quantitative PCR was used to detect IL-6and TNF-αmRNA. Data represent as mean ± SEM. Compared to con, *** *p*< 0.001, **** *p* < 0.0001; compared to a concentration of 0 μM, # *p*< 0.05, ###*p* < 0.001, #### *p* < 0.0001 and ns: not significant

## Data Availability

Data is provided within this published article and its supplementary information files.

## Abbreviations

AIM2: Absent In Melanoma 2
ASC: Apoptosis-associated speck-like protein containing a CARD
ATP: Adenosine Triphosphate
BMDMs: Bone marrow-derived macrophages
CoIP: Co-Immunoprecipitation
DAMP: Damage-Associated Molecular Patterns;
DMEM: Dulbecco's Modified Eagle Medium
DSS: Disuccinimidyl suberate
EDTA: Ethylenediaminetetraacetic acid
ESI: Electrospray Ionization
GSDMD: Gasdermin D
HBSS: Hanks' Balanced Salt Solution
IL-1β: Interleukin-1 beta
IL-6: Interleukin-6
LDH: Lactate dehydrogenase
LPS: Lipopolysaccharide
MCSF: Murine macrophage colony-stimulating factor
NACHT: NAIP, CIITA, HET-E, and TP1 domains
NF-κB: Nuclear Factor-κB
NLRC4: NLR Family, CARD Domain Containing 4
NLRP3: NLR family, Pyrin domain containing protein 3
PAMP: Pathogen-Associated Molecular Patterns
qPCR: Quantitative Polymerase Chain Reaction
ROS: Reactive oxygen species
SDS-PAGE: Sodium Dodecyl Sulfate–Polyacrylamide Gel Electrophoresis
SN: Supernatant
TCA: Trichloroacetic acid
TLRs: Toll-like Receptors
TNF-α: Tumor necrosis factor-alpha
WCL: Whole cell lysate
