# Supplementary figures and images for: Investigating the impact and mechanism of Licochalcone B derivative CTG12 on NLRP3 inflammasome
Source: Cell Commun Signal. 2026 Feb 18;24:196. doi: 10.1186/s12964-026-02741-2 (PMC13020283; doi:10.1186/s12964-026-02741-2)

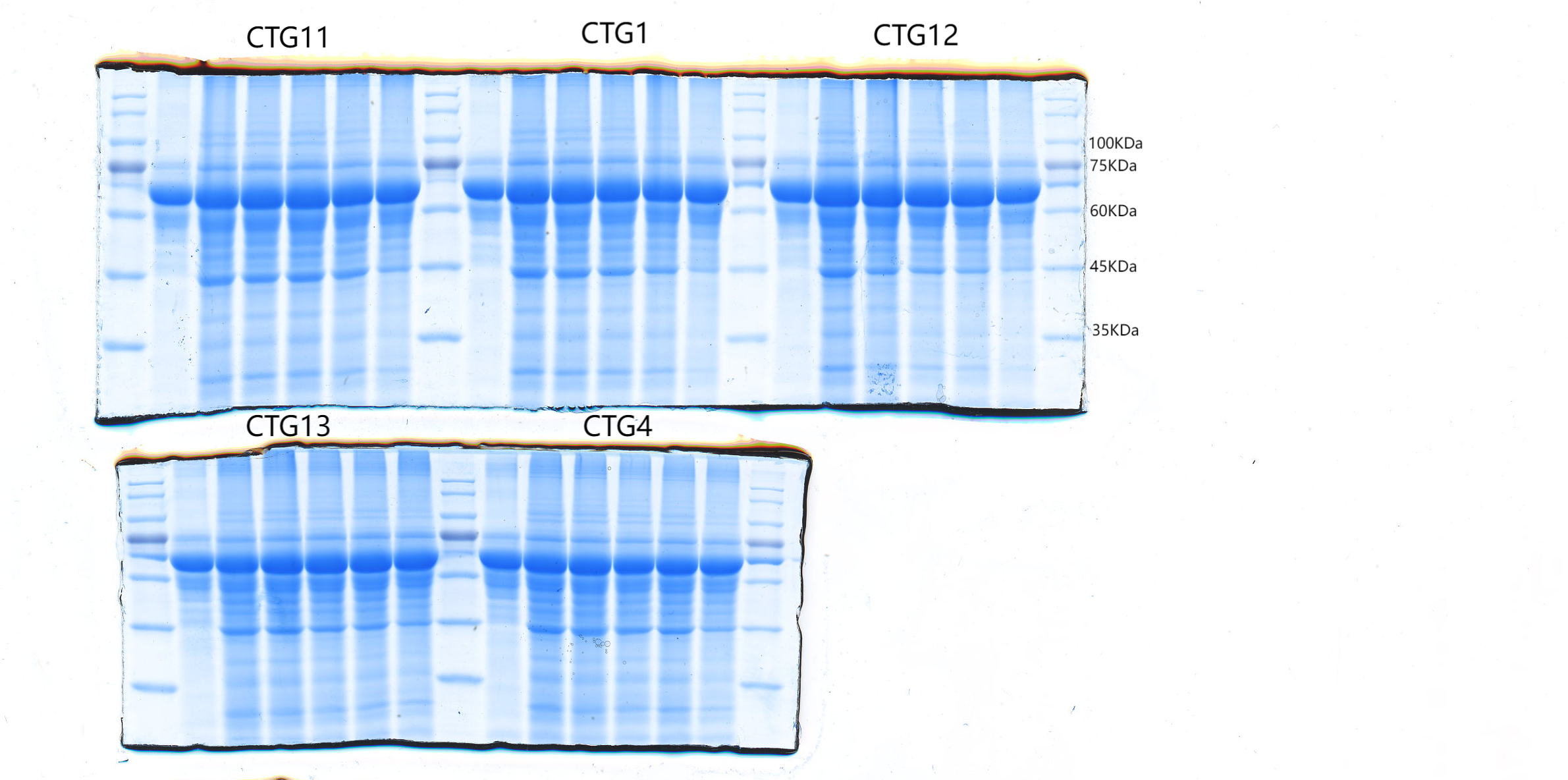

Supplement: Supplementary file 1 — Supplementary Material 1. Supplementary Figure 1. Licochalcone B derivatives inhibit NLRP3 inflammasome activation.BMDMs were primed with LPS for 4 hours, treated with Echinatin, Licorice Chalcone B, CTG4, CTG1, CTG10, CTG11, CTG12, CTG13, CTG14, CTG15, CTG16, CTG18, CTG19, CAPE, CTG23for 30 minutes, and then stimulated with nigericin for 25 minutes. Supernatants were collected for the measurement of caspase-1. Data represent as mean ± SEM. Compared to con, **** p < 0.0001; compared to a concentration of 0 μM, ###p < 0.001, #### p < 0.0001 and ns:not significant. Supplementary Figure 2. CTG11 and CTG13 inhibit NLRP3 inflammasome activation in mouse BMDMs.The structure of CTG11.Western blot analysis of IL-1β, caspase-1in culture supernatantsand pro-IL-1β, caspase-1, NLRP3, ASC in whole cell lysatesof LPS-primed BMDMs treated with CTG11 and then stimulated with Nigericin, supernatants were collected for the measurement of caspase-1, IL-1β, LDHand TNF-α.The structure of CTG13.Western blot analysis of IL-1β, caspase-1in culture supernatantsand pro-IL-1β, caspase-1, NLRP3, ASC in whole cell lysatesof LPS-primed BMDMs treated with CTG13 and then stimulated with Nigericin, supernatants were collected for the measurement of caspase-1, IL-1β, LDHand TNF-α. Coomassie blue–stained gels used as loading control and Lamin B used as a control for equal loading of the samples. Data represent as mean ± SEM. Compared to con, ** p < 0.01, ***p < 0.001, **** p < 0.0001; compared to a concentration of 0 μM, ###p < 0.001, ####p < 0.0001 and ns: not significant. Supplementary Figure 3. CTG12 impedes the priming process of NLRP3 inflammasome activation and specifically inhibits canonical and noncanonical NLRP3 inflammasome activation.BMDMs were primed with LPS treated with CTG12, then stimulated with Nigericin ATP, poly, or SiO₂. Supernatants were collected for the measurement of TNF-α, BMDMs primed with Pam3CSK4 treated with CTG12, followed by cytosolic LPS. Supernatants were collected fo [file 12964_2026_2741_MOESM1_ESM.zip › supplementary file/Figure1-Figure2-Supplementary Figure1原膜/Figure1-CTG4-CTG1-Figure2-CTG12-Supplementary Figure1 BMDM细胞-CTG11-CTG13-Coomassie条带.png]

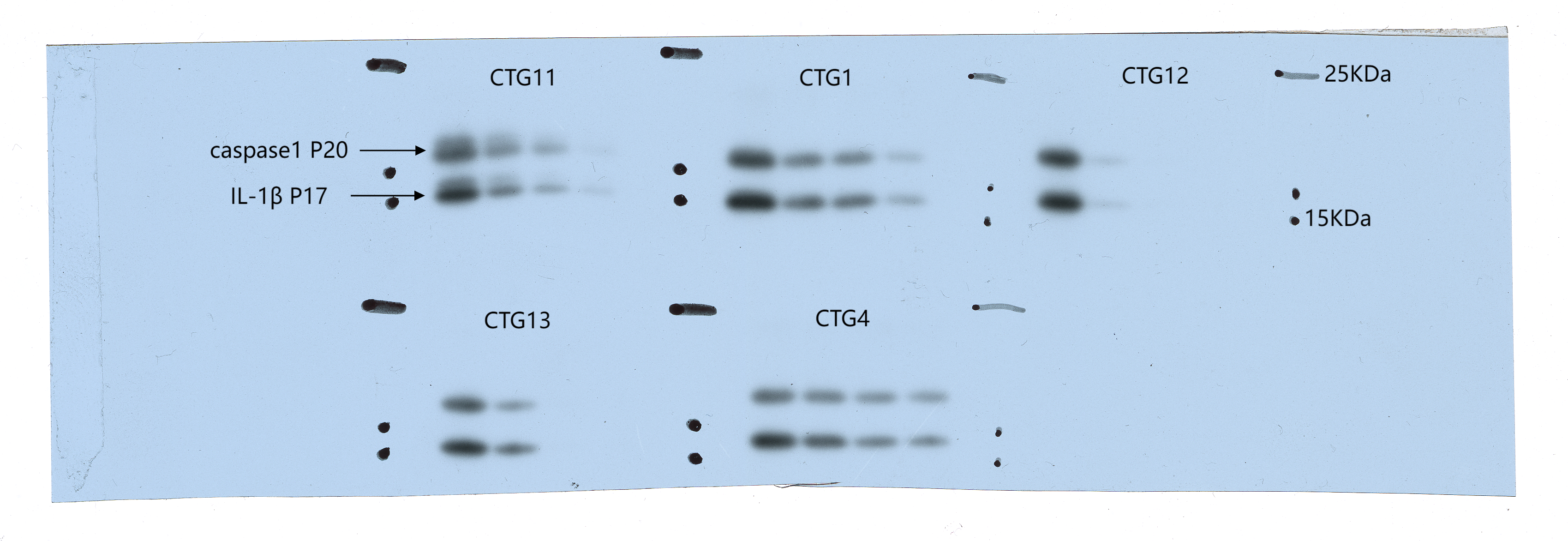

Supplement: Supplementary file 1 — Supplementary Material 1. Supplementary Figure 1. Licochalcone B derivatives inhibit NLRP3 inflammasome activation.BMDMs were primed with LPS for 4 hours, treated with Echinatin, Licorice Chalcone B, CTG4, CTG1, CTG10, CTG11, CTG12, CTG13, CTG14, CTG15, CTG16, CTG18, CTG19, CAPE, CTG23for 30 minutes, and then stimulated with nigericin for 25 minutes. Supernatants were collected for the measurement of caspase-1. Data represent as mean ± SEM. Compared to con, **** p < 0.0001; compared to a concentration of 0 μM, ###p < 0.001, #### p < 0.0001 and ns:not significant. Supplementary Figure 2. CTG11 and CTG13 inhibit NLRP3 inflammasome activation in mouse BMDMs.The structure of CTG11.Western blot analysis of IL-1β, caspase-1in culture supernatantsand pro-IL-1β, caspase-1, NLRP3, ASC in whole cell lysatesof LPS-primed BMDMs treated with CTG11 and then stimulated with Nigericin, supernatants were collected for the measurement of caspase-1, IL-1β, LDHand TNF-α.The structure of CTG13.Western blot analysis of IL-1β, caspase-1in culture supernatantsand pro-IL-1β, caspase-1, NLRP3, ASC in whole cell lysatesof LPS-primed BMDMs treated with CTG13 and then stimulated with Nigericin, supernatants were collected for the measurement of caspase-1, IL-1β, LDHand TNF-α. Coomassie blue–stained gels used as loading control and Lamin B used as a control for equal loading of the samples. Data represent as mean ± SEM. Compared to con, ** p < 0.01, ***p < 0.001, **** p < 0.0001; compared to a concentration of 0 μM, ###p < 0.001, ####p < 0.0001 and ns: not significant. Supplementary Figure 3. CTG12 impedes the priming process of NLRP3 inflammasome activation and specifically inhibits canonical and noncanonical NLRP3 inflammasome activation.BMDMs were primed with LPS treated with CTG12, then stimulated with Nigericin ATP, poly, or SiO₂. Supernatants were collected for the measurement of TNF-α, BMDMs primed with Pam3CSK4 treated with CTG12, followed by cytosolic LPS. Supernatants were collected fo [file 12964_2026_2741_MOESM1_ESM.zip › supplementary file/Figure1-Figure2-Supplementary Figure1原膜/Figure1-CTG4-CTG1-Figure2-CTG12-Supplementary Figure1 CTG11-CTG13-BMDM细胞-Caspase1 P20-IL1b P17条带.png]

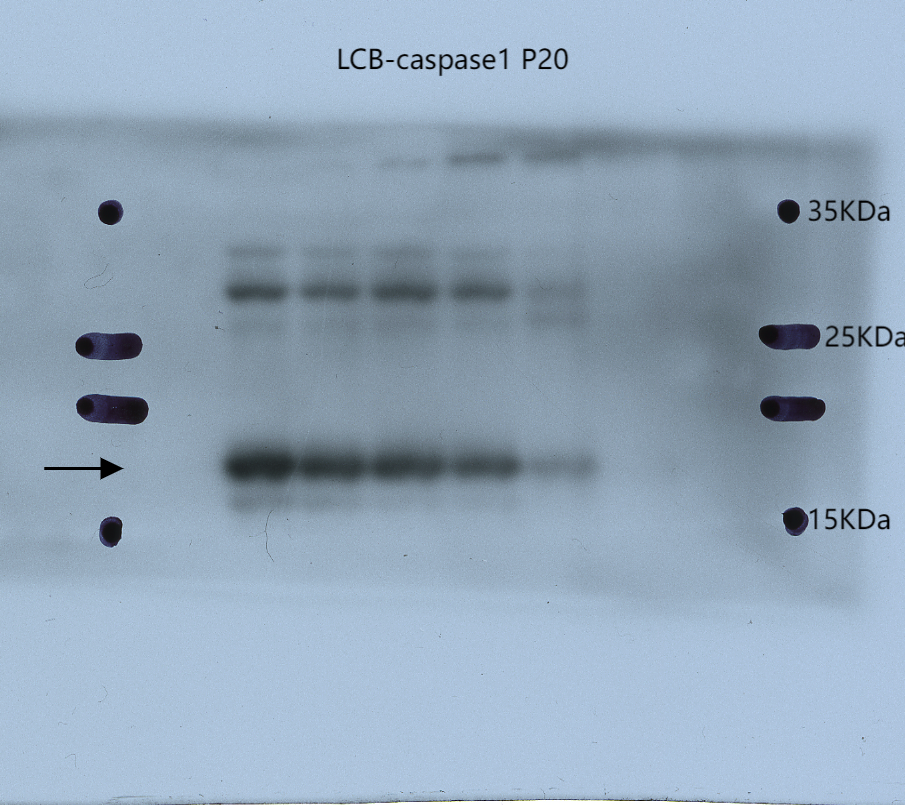

Supplement: Supplementary file 1 — Supplementary Material 1. Supplementary Figure 1. Licochalcone B derivatives inhibit NLRP3 inflammasome activation.BMDMs were primed with LPS for 4 hours, treated with Echinatin, Licorice Chalcone B, CTG4, CTG1, CTG10, CTG11, CTG12, CTG13, CTG14, CTG15, CTG16, CTG18, CTG19, CAPE, CTG23for 30 minutes, and then stimulated with nigericin for 25 minutes. Supernatants were collected for the measurement of caspase-1. Data represent as mean ± SEM. Compared to con, **** p < 0.0001; compared to a concentration of 0 μM, ###p < 0.001, #### p < 0.0001 and ns:not significant. Supplementary Figure 2. CTG11 and CTG13 inhibit NLRP3 inflammasome activation in mouse BMDMs.The structure of CTG11.Western blot analysis of IL-1β, caspase-1in culture supernatantsand pro-IL-1β, caspase-1, NLRP3, ASC in whole cell lysatesof LPS-primed BMDMs treated with CTG11 and then stimulated with Nigericin, supernatants were collected for the measurement of caspase-1, IL-1β, LDHand TNF-α.The structure of CTG13.Western blot analysis of IL-1β, caspase-1in culture supernatantsand pro-IL-1β, caspase-1, NLRP3, ASC in whole cell lysatesof LPS-primed BMDMs treated with CTG13 and then stimulated with Nigericin, supernatants were collected for the measurement of caspase-1, IL-1β, LDHand TNF-α. Coomassie blue–stained gels used as loading control and Lamin B used as a control for equal loading of the samples. Data represent as mean ± SEM. Compared to con, ** p < 0.01, ***p < 0.001, **** p < 0.0001; compared to a concentration of 0 μM, ###p < 0.001, ####p < 0.0001 and ns: not significant. Supplementary Figure 3. CTG12 impedes the priming process of NLRP3 inflammasome activation and specifically inhibits canonical and noncanonical NLRP3 inflammasome activation.BMDMs were primed with LPS treated with CTG12, then stimulated with Nigericin ATP, poly, or SiO₂. Supernatants were collected for the measurement of TNF-α, BMDMs primed with Pam3CSK4 treated with CTG12, followed by cytosolic LPS. Supernatants were collected fo [file 12964_2026_2741_MOESM1_ESM.zip › supplementary file/Figure1-Figure2-Supplementary Figure1原膜/Figure1-LCB-Caspase1 P20条带.png]

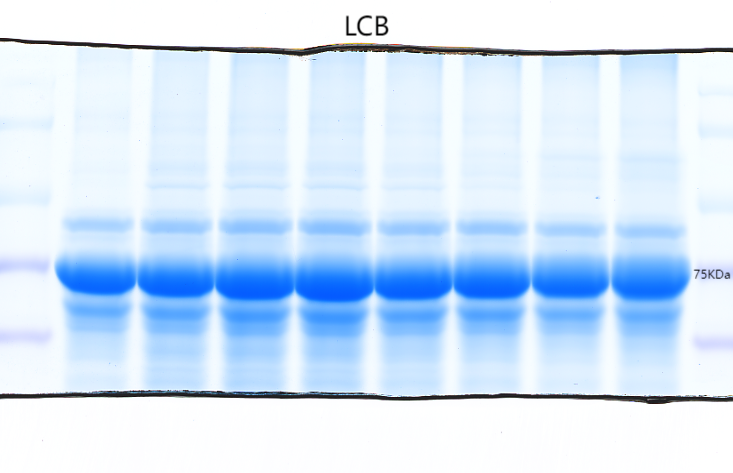

Supplement: Supplementary file 1 — Supplementary Material 1. Supplementary Figure 1. Licochalcone B derivatives inhibit NLRP3 inflammasome activation.BMDMs were primed with LPS for 4 hours, treated with Echinatin, Licorice Chalcone B, CTG4, CTG1, CTG10, CTG11, CTG12, CTG13, CTG14, CTG15, CTG16, CTG18, CTG19, CAPE, CTG23for 30 minutes, and then stimulated with nigericin for 25 minutes. Supernatants were collected for the measurement of caspase-1. Data represent as mean ± SEM. Compared to con, **** p < 0.0001; compared to a concentration of 0 μM, ###p < 0.001, #### p < 0.0001 and ns:not significant. Supplementary Figure 2. CTG11 and CTG13 inhibit NLRP3 inflammasome activation in mouse BMDMs.The structure of CTG11.Western blot analysis of IL-1β, caspase-1in culture supernatantsand pro-IL-1β, caspase-1, NLRP3, ASC in whole cell lysatesof LPS-primed BMDMs treated with CTG11 and then stimulated with Nigericin, supernatants were collected for the measurement of caspase-1, IL-1β, LDHand TNF-α.The structure of CTG13.Western blot analysis of IL-1β, caspase-1in culture supernatantsand pro-IL-1β, caspase-1, NLRP3, ASC in whole cell lysatesof LPS-primed BMDMs treated with CTG13 and then stimulated with Nigericin, supernatants were collected for the measurement of caspase-1, IL-1β, LDHand TNF-α. Coomassie blue–stained gels used as loading control and Lamin B used as a control for equal loading of the samples. Data represent as mean ± SEM. Compared to con, ** p < 0.01, ***p < 0.001, **** p < 0.0001; compared to a concentration of 0 μM, ###p < 0.001, ####p < 0.0001 and ns: not significant. Supplementary Figure 3. CTG12 impedes the priming process of NLRP3 inflammasome activation and specifically inhibits canonical and noncanonical NLRP3 inflammasome activation.BMDMs were primed with LPS treated with CTG12, then stimulated with Nigericin ATP, poly, or SiO₂. Supernatants were collected for the measurement of TNF-α, BMDMs primed with Pam3CSK4 treated with CTG12, followed by cytosolic LPS. Supernatants were collected fo [file 12964_2026_2741_MOESM1_ESM.zip › supplementary file/Figure1-Figure2-Supplementary Figure1原膜/Figure1-LCB-Coomassie条带.png]

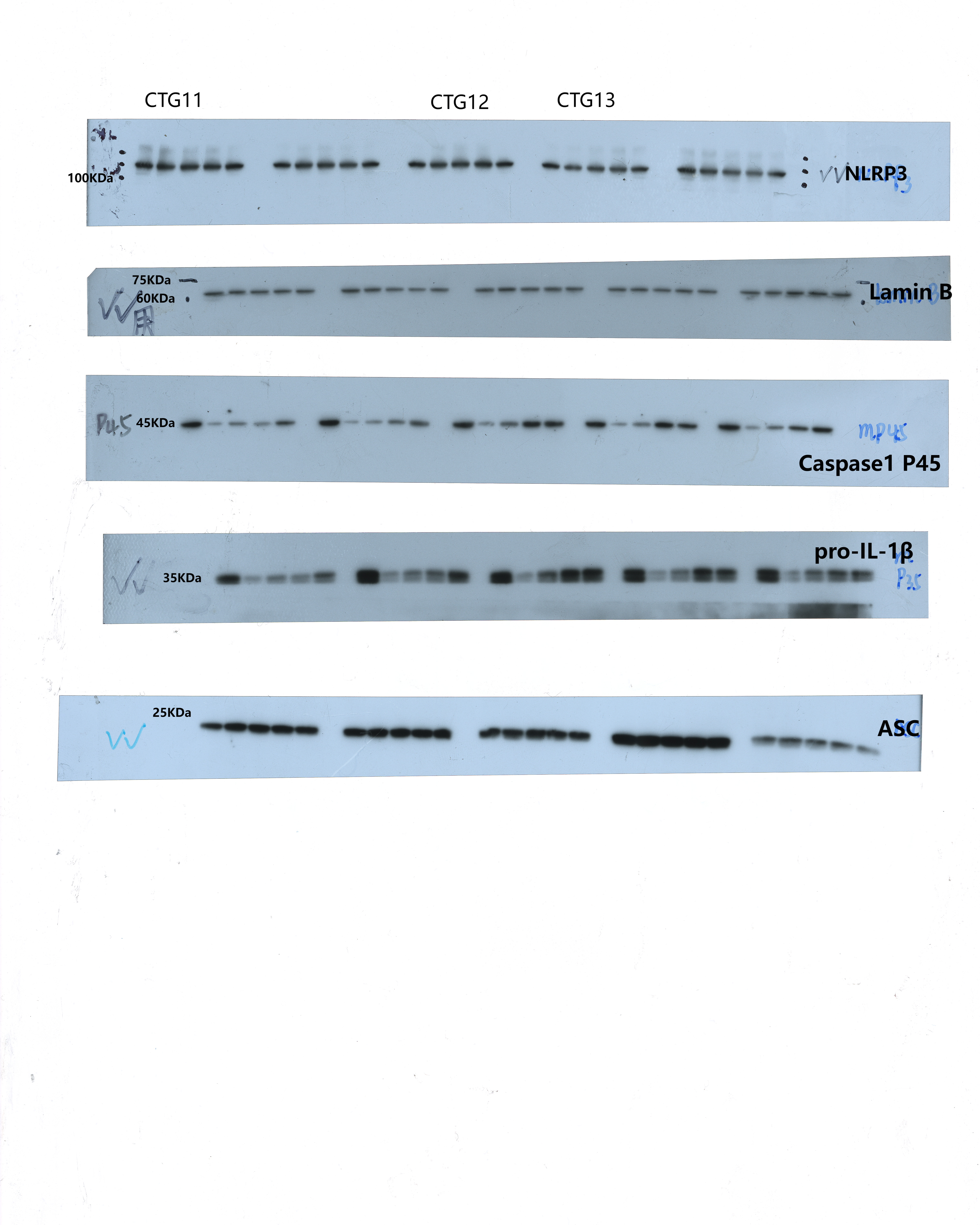

Supplement: Supplementary file 1 — Supplementary Material 1. Supplementary Figure 1. Licochalcone B derivatives inhibit NLRP3 inflammasome activation.BMDMs were primed with LPS for 4 hours, treated with Echinatin, Licorice Chalcone B, CTG4, CTG1, CTG10, CTG11, CTG12, CTG13, CTG14, CTG15, CTG16, CTG18, CTG19, CAPE, CTG23for 30 minutes, and then stimulated with nigericin for 25 minutes. Supernatants were collected for the measurement of caspase-1. Data represent as mean ± SEM. Compared to con, **** p < 0.0001; compared to a concentration of 0 μM, ###p < 0.001, #### p < 0.0001 and ns:not significant. Supplementary Figure 2. CTG11 and CTG13 inhibit NLRP3 inflammasome activation in mouse BMDMs.The structure of CTG11.Western blot analysis of IL-1β, caspase-1in culture supernatantsand pro-IL-1β, caspase-1, NLRP3, ASC in whole cell lysatesof LPS-primed BMDMs treated with CTG11 and then stimulated with Nigericin, supernatants were collected for the measurement of caspase-1, IL-1β, LDHand TNF-α.The structure of CTG13.Western blot analysis of IL-1β, caspase-1in culture supernatantsand pro-IL-1β, caspase-1, NLRP3, ASC in whole cell lysatesof LPS-primed BMDMs treated with CTG13 and then stimulated with Nigericin, supernatants were collected for the measurement of caspase-1, IL-1β, LDHand TNF-α. Coomassie blue–stained gels used as loading control and Lamin B used as a control for equal loading of the samples. Data represent as mean ± SEM. Compared to con, ** p < 0.01, ***p < 0.001, **** p < 0.0001; compared to a concentration of 0 μM, ###p < 0.001, ####p < 0.0001 and ns: not significant. Supplementary Figure 3. CTG12 impedes the priming process of NLRP3 inflammasome activation and specifically inhibits canonical and noncanonical NLRP3 inflammasome activation.BMDMs were primed with LPS treated with CTG12, then stimulated with Nigericin ATP, poly, or SiO₂. Supernatants were collected for the measurement of TNF-α, BMDMs primed with Pam3CSK4 treated with CTG12, followed by cytosolic LPS. Supernatants were collected fo [file 12964_2026_2741_MOESM1_ESM.zip › supplementary file/Figure1-Figure2-Supplementary Figure1原膜/Figure2-CTG12-Supplementary Figure1 CTG11-CTG13-BMDM细胞-lamin b-ASC-capase1-P45-pro-il-1β-NLRP3条带.png]

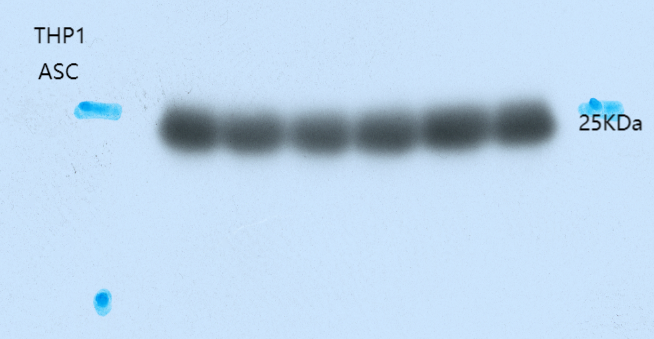

Supplement: Supplementary file 1 — Supplementary Material 1. Supplementary Figure 1. Licochalcone B derivatives inhibit NLRP3 inflammasome activation.BMDMs were primed with LPS for 4 hours, treated with Echinatin, Licorice Chalcone B, CTG4, CTG1, CTG10, CTG11, CTG12, CTG13, CTG14, CTG15, CTG16, CTG18, CTG19, CAPE, CTG23for 30 minutes, and then stimulated with nigericin for 25 minutes. Supernatants were collected for the measurement of caspase-1. Data represent as mean ± SEM. Compared to con, **** p < 0.0001; compared to a concentration of 0 μM, ###p < 0.001, #### p < 0.0001 and ns:not significant. Supplementary Figure 2. CTG11 and CTG13 inhibit NLRP3 inflammasome activation in mouse BMDMs.The structure of CTG11.Western blot analysis of IL-1β, caspase-1in culture supernatantsand pro-IL-1β, caspase-1, NLRP3, ASC in whole cell lysatesof LPS-primed BMDMs treated with CTG11 and then stimulated with Nigericin, supernatants were collected for the measurement of caspase-1, IL-1β, LDHand TNF-α.The structure of CTG13.Western blot analysis of IL-1β, caspase-1in culture supernatantsand pro-IL-1β, caspase-1, NLRP3, ASC in whole cell lysatesof LPS-primed BMDMs treated with CTG13 and then stimulated with Nigericin, supernatants were collected for the measurement of caspase-1, IL-1β, LDHand TNF-α. Coomassie blue–stained gels used as loading control and Lamin B used as a control for equal loading of the samples. Data represent as mean ± SEM. Compared to con, ** p < 0.01, ***p < 0.001, **** p < 0.0001; compared to a concentration of 0 μM, ###p < 0.001, ####p < 0.0001 and ns: not significant. Supplementary Figure 3. CTG12 impedes the priming process of NLRP3 inflammasome activation and specifically inhibits canonical and noncanonical NLRP3 inflammasome activation.BMDMs were primed with LPS treated with CTG12, then stimulated with Nigericin ATP, poly, or SiO₂. Supernatants were collected for the measurement of TNF-α, BMDMs primed with Pam3CSK4 treated with CTG12, followed by cytosolic LPS. Supernatants were collected fo [file 12964_2026_2741_MOESM1_ESM.zip › supplementary file/Figure1-Figure2-Supplementary Figure1原膜/Figure2-CTG12-THP1细胞-ASC.png]

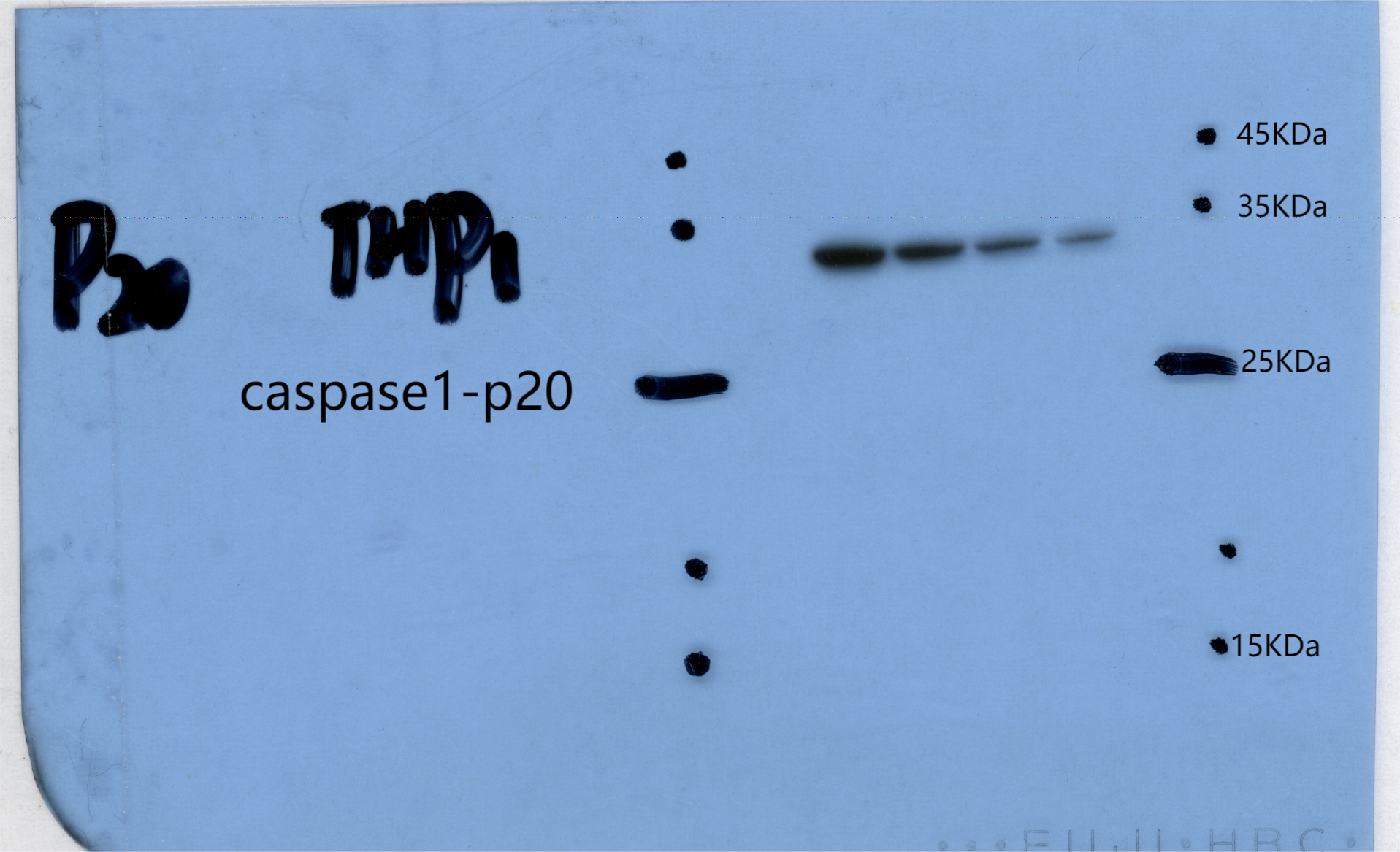

Supplement: Supplementary file 1 — Supplementary Material 1. Supplementary Figure 1. Licochalcone B derivatives inhibit NLRP3 inflammasome activation.BMDMs were primed with LPS for 4 hours, treated with Echinatin, Licorice Chalcone B, CTG4, CTG1, CTG10, CTG11, CTG12, CTG13, CTG14, CTG15, CTG16, CTG18, CTG19, CAPE, CTG23for 30 minutes, and then stimulated with nigericin for 25 minutes. Supernatants were collected for the measurement of caspase-1. Data represent as mean ± SEM. Compared to con, **** p < 0.0001; compared to a concentration of 0 μM, ###p < 0.001, #### p < 0.0001 and ns:not significant. Supplementary Figure 2. CTG11 and CTG13 inhibit NLRP3 inflammasome activation in mouse BMDMs.The structure of CTG11.Western blot analysis of IL-1β, caspase-1in culture supernatantsand pro-IL-1β, caspase-1, NLRP3, ASC in whole cell lysatesof LPS-primed BMDMs treated with CTG11 and then stimulated with Nigericin, supernatants were collected for the measurement of caspase-1, IL-1β, LDHand TNF-α.The structure of CTG13.Western blot analysis of IL-1β, caspase-1in culture supernatantsand pro-IL-1β, caspase-1, NLRP3, ASC in whole cell lysatesof LPS-primed BMDMs treated with CTG13 and then stimulated with Nigericin, supernatants were collected for the measurement of caspase-1, IL-1β, LDHand TNF-α. Coomassie blue–stained gels used as loading control and Lamin B used as a control for equal loading of the samples. Data represent as mean ± SEM. Compared to con, ** p < 0.01, ***p < 0.001, **** p < 0.0001; compared to a concentration of 0 μM, ###p < 0.001, ####p < 0.0001 and ns: not significant. Supplementary Figure 3. CTG12 impedes the priming process of NLRP3 inflammasome activation and specifically inhibits canonical and noncanonical NLRP3 inflammasome activation.BMDMs were primed with LPS treated with CTG12, then stimulated with Nigericin ATP, poly, or SiO₂. Supernatants were collected for the measurement of TNF-α, BMDMs primed with Pam3CSK4 treated with CTG12, followed by cytosolic LPS. Supernatants were collected fo [file 12964_2026_2741_MOESM1_ESM.zip › supplementary file/Figure1-Figure2-Supplementary Figure1原膜/Figure2-CTG12-THP1细胞-caspase1 p20条带.png]

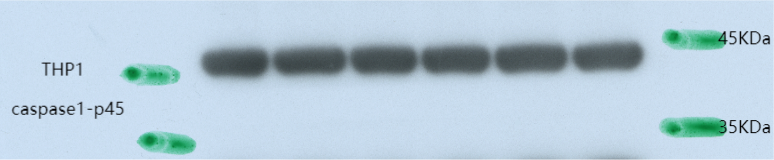

Supplement: Supplementary file 1 — Supplementary Material 1. Supplementary Figure 1. Licochalcone B derivatives inhibit NLRP3 inflammasome activation.BMDMs were primed with LPS for 4 hours, treated with Echinatin, Licorice Chalcone B, CTG4, CTG1, CTG10, CTG11, CTG12, CTG13, CTG14, CTG15, CTG16, CTG18, CTG19, CAPE, CTG23for 30 minutes, and then stimulated with nigericin for 25 minutes. Supernatants were collected for the measurement of caspase-1. Data represent as mean ± SEM. Compared to con, **** p < 0.0001; compared to a concentration of 0 μM, ###p < 0.001, #### p < 0.0001 and ns:not significant. Supplementary Figure 2. CTG11 and CTG13 inhibit NLRP3 inflammasome activation in mouse BMDMs.The structure of CTG11.Western blot analysis of IL-1β, caspase-1in culture supernatantsand pro-IL-1β, caspase-1, NLRP3, ASC in whole cell lysatesof LPS-primed BMDMs treated with CTG11 and then stimulated with Nigericin, supernatants were collected for the measurement of caspase-1, IL-1β, LDHand TNF-α.The structure of CTG13.Western blot analysis of IL-1β, caspase-1in culture supernatantsand pro-IL-1β, caspase-1, NLRP3, ASC in whole cell lysatesof LPS-primed BMDMs treated with CTG13 and then stimulated with Nigericin, supernatants were collected for the measurement of caspase-1, IL-1β, LDHand TNF-α. Coomassie blue–stained gels used as loading control and Lamin B used as a control for equal loading of the samples. Data represent as mean ± SEM. Compared to con, ** p < 0.01, ***p < 0.001, **** p < 0.0001; compared to a concentration of 0 μM, ###p < 0.001, ####p < 0.0001 and ns: not significant. Supplementary Figure 3. CTG12 impedes the priming process of NLRP3 inflammasome activation and specifically inhibits canonical and noncanonical NLRP3 inflammasome activation.BMDMs were primed with LPS treated with CTG12, then stimulated with Nigericin ATP, poly, or SiO₂. Supernatants were collected for the measurement of TNF-α, BMDMs primed with Pam3CSK4 treated with CTG12, followed by cytosolic LPS. Supernatants were collected fo [file 12964_2026_2741_MOESM1_ESM.zip › supplementary file/Figure1-Figure2-Supplementary Figure1原膜/Figure2-CTG12-THP1细胞-caspase1-p45.png]

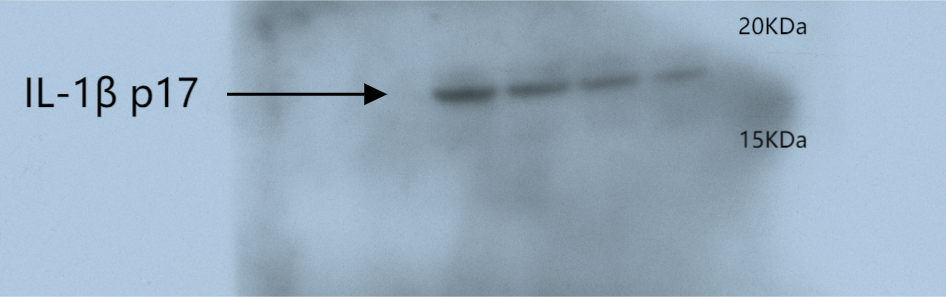

Supplement: Supplementary file 1 — Supplementary Material 1. Supplementary Figure 1. Licochalcone B derivatives inhibit NLRP3 inflammasome activation.BMDMs were primed with LPS for 4 hours, treated with Echinatin, Licorice Chalcone B, CTG4, CTG1, CTG10, CTG11, CTG12, CTG13, CTG14, CTG15, CTG16, CTG18, CTG19, CAPE, CTG23for 30 minutes, and then stimulated with nigericin for 25 minutes. Supernatants were collected for the measurement of caspase-1. Data represent as mean ± SEM. Compared to con, **** p < 0.0001; compared to a concentration of 0 μM, ###p < 0.001, #### p < 0.0001 and ns:not significant. Supplementary Figure 2. CTG11 and CTG13 inhibit NLRP3 inflammasome activation in mouse BMDMs.The structure of CTG11.Western blot analysis of IL-1β, caspase-1in culture supernatantsand pro-IL-1β, caspase-1, NLRP3, ASC in whole cell lysatesof LPS-primed BMDMs treated with CTG11 and then stimulated with Nigericin, supernatants were collected for the measurement of caspase-1, IL-1β, LDHand TNF-α.The structure of CTG13.Western blot analysis of IL-1β, caspase-1in culture supernatantsand pro-IL-1β, caspase-1, NLRP3, ASC in whole cell lysatesof LPS-primed BMDMs treated with CTG13 and then stimulated with Nigericin, supernatants were collected for the measurement of caspase-1, IL-1β, LDHand TNF-α. Coomassie blue–stained gels used as loading control and Lamin B used as a control for equal loading of the samples. Data represent as mean ± SEM. Compared to con, ** p < 0.01, ***p < 0.001, **** p < 0.0001; compared to a concentration of 0 μM, ###p < 0.001, ####p < 0.0001 and ns: not significant. Supplementary Figure 3. CTG12 impedes the priming process of NLRP3 inflammasome activation and specifically inhibits canonical and noncanonical NLRP3 inflammasome activation.BMDMs were primed with LPS treated with CTG12, then stimulated with Nigericin ATP, poly, or SiO₂. Supernatants were collected for the measurement of TNF-α, BMDMs primed with Pam3CSK4 treated with CTG12, followed by cytosolic LPS. Supernatants were collected fo [file 12964_2026_2741_MOESM1_ESM.zip › supplementary file/Figure1-Figure2-Supplementary Figure1原膜/Figure2-CTG12-THP1细胞-IL-1β P17条带.png]

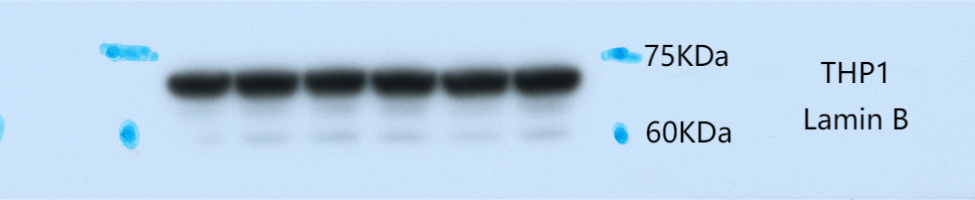

Supplement: Supplementary file 1 — Supplementary Material 1. Supplementary Figure 1. Licochalcone B derivatives inhibit NLRP3 inflammasome activation.BMDMs were primed with LPS for 4 hours, treated with Echinatin, Licorice Chalcone B, CTG4, CTG1, CTG10, CTG11, CTG12, CTG13, CTG14, CTG15, CTG16, CTG18, CTG19, CAPE, CTG23for 30 minutes, and then stimulated with nigericin for 25 minutes. Supernatants were collected for the measurement of caspase-1. Data represent as mean ± SEM. Compared to con, **** p < 0.0001; compared to a concentration of 0 μM, ###p < 0.001, #### p < 0.0001 and ns:not significant. Supplementary Figure 2. CTG11 and CTG13 inhibit NLRP3 inflammasome activation in mouse BMDMs.The structure of CTG11.Western blot analysis of IL-1β, caspase-1in culture supernatantsand pro-IL-1β, caspase-1, NLRP3, ASC in whole cell lysatesof LPS-primed BMDMs treated with CTG11 and then stimulated with Nigericin, supernatants were collected for the measurement of caspase-1, IL-1β, LDHand TNF-α.The structure of CTG13.Western blot analysis of IL-1β, caspase-1in culture supernatantsand pro-IL-1β, caspase-1, NLRP3, ASC in whole cell lysatesof LPS-primed BMDMs treated with CTG13 and then stimulated with Nigericin, supernatants were collected for the measurement of caspase-1, IL-1β, LDHand TNF-α. Coomassie blue–stained gels used as loading control and Lamin B used as a control for equal loading of the samples. Data represent as mean ± SEM. Compared to con, ** p < 0.01, ***p < 0.001, **** p < 0.0001; compared to a concentration of 0 μM, ###p < 0.001, ####p < 0.0001 and ns: not significant. Supplementary Figure 3. CTG12 impedes the priming process of NLRP3 inflammasome activation and specifically inhibits canonical and noncanonical NLRP3 inflammasome activation.BMDMs were primed with LPS treated with CTG12, then stimulated with Nigericin ATP, poly, or SiO₂. Supernatants were collected for the measurement of TNF-α, BMDMs primed with Pam3CSK4 treated with CTG12, followed by cytosolic LPS. Supernatants were collected fo [file 12964_2026_2741_MOESM1_ESM.zip › supplementary file/Figure1-Figure2-Supplementary Figure1原膜/Figure2-CTG12-THP1细胞-lamin B.png]

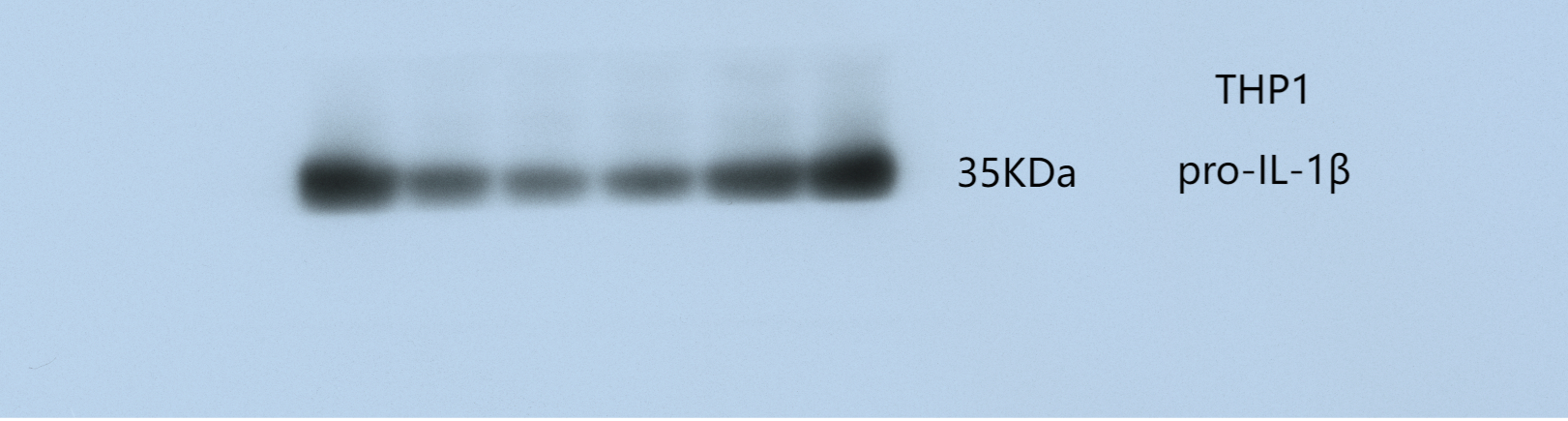

Supplement: Supplementary file 1 — Supplementary Material 1. Supplementary Figure 1. Licochalcone B derivatives inhibit NLRP3 inflammasome activation.BMDMs were primed with LPS for 4 hours, treated with Echinatin, Licorice Chalcone B, CTG4, CTG1, CTG10, CTG11, CTG12, CTG13, CTG14, CTG15, CTG16, CTG18, CTG19, CAPE, CTG23for 30 minutes, and then stimulated with nigericin for 25 minutes. Supernatants were collected for the measurement of caspase-1. Data represent as mean ± SEM. Compared to con, **** p < 0.0001; compared to a concentration of 0 μM, ###p < 0.001, #### p < 0.0001 and ns:not significant. Supplementary Figure 2. CTG11 and CTG13 inhibit NLRP3 inflammasome activation in mouse BMDMs.The structure of CTG11.Western blot analysis of IL-1β, caspase-1in culture supernatantsand pro-IL-1β, caspase-1, NLRP3, ASC in whole cell lysatesof LPS-primed BMDMs treated with CTG11 and then stimulated with Nigericin, supernatants were collected for the measurement of caspase-1, IL-1β, LDHand TNF-α.The structure of CTG13.Western blot analysis of IL-1β, caspase-1in culture supernatantsand pro-IL-1β, caspase-1, NLRP3, ASC in whole cell lysatesof LPS-primed BMDMs treated with CTG13 and then stimulated with Nigericin, supernatants were collected for the measurement of caspase-1, IL-1β, LDHand TNF-α. Coomassie blue–stained gels used as loading control and Lamin B used as a control for equal loading of the samples. Data represent as mean ± SEM. Compared to con, ** p < 0.01, ***p < 0.001, **** p < 0.0001; compared to a concentration of 0 μM, ###p < 0.001, ####p < 0.0001 and ns: not significant. Supplementary Figure 3. CTG12 impedes the priming process of NLRP3 inflammasome activation and specifically inhibits canonical and noncanonical NLRP3 inflammasome activation.BMDMs were primed with LPS treated with CTG12, then stimulated with Nigericin ATP, poly, or SiO₂. Supernatants were collected for the measurement of TNF-α, BMDMs primed with Pam3CSK4 treated with CTG12, followed by cytosolic LPS. Supernatants were collected fo [file 12964_2026_2741_MOESM1_ESM.zip › supplementary file/Figure1-Figure2-Supplementary Figure1原膜/Figure2-CTG12-THP1细胞-pro-IL-1β.png]

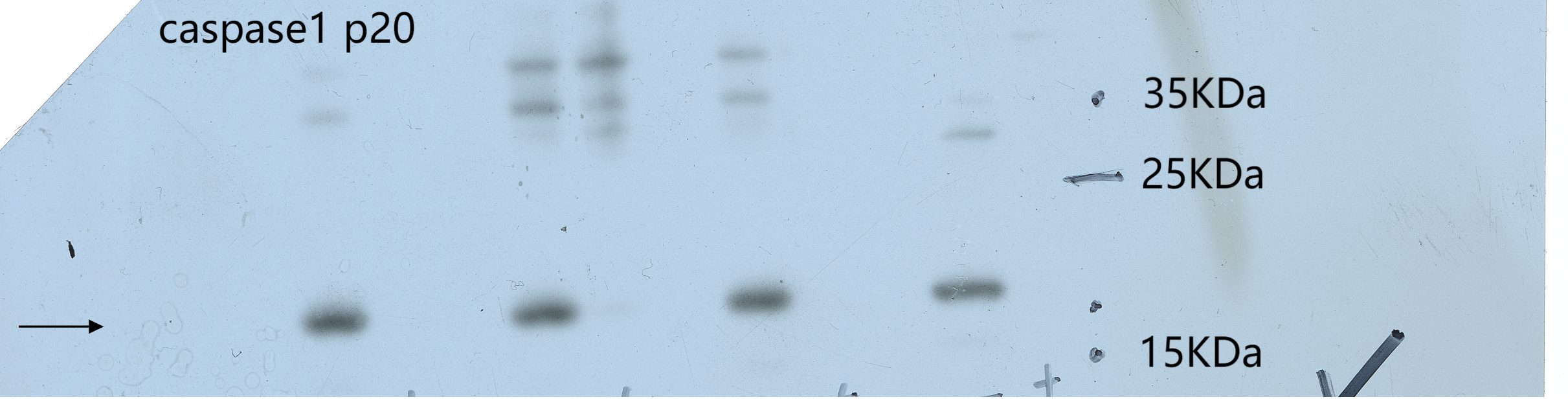

Supplement: Supplementary file 1 — Supplementary Material 1. Supplementary Figure 1. Licochalcone B derivatives inhibit NLRP3 inflammasome activation.BMDMs were primed with LPS for 4 hours, treated with Echinatin, Licorice Chalcone B, CTG4, CTG1, CTG10, CTG11, CTG12, CTG13, CTG14, CTG15, CTG16, CTG18, CTG19, CAPE, CTG23for 30 minutes, and then stimulated with nigericin for 25 minutes. Supernatants were collected for the measurement of caspase-1. Data represent as mean ± SEM. Compared to con, **** p < 0.0001; compared to a concentration of 0 μM, ###p < 0.001, #### p < 0.0001 and ns:not significant. Supplementary Figure 2. CTG11 and CTG13 inhibit NLRP3 inflammasome activation in mouse BMDMs.The structure of CTG11.Western blot analysis of IL-1β, caspase-1in culture supernatantsand pro-IL-1β, caspase-1, NLRP3, ASC in whole cell lysatesof LPS-primed BMDMs treated with CTG11 and then stimulated with Nigericin, supernatants were collected for the measurement of caspase-1, IL-1β, LDHand TNF-α.The structure of CTG13.Western blot analysis of IL-1β, caspase-1in culture supernatantsand pro-IL-1β, caspase-1, NLRP3, ASC in whole cell lysatesof LPS-primed BMDMs treated with CTG13 and then stimulated with Nigericin, supernatants were collected for the measurement of caspase-1, IL-1β, LDHand TNF-α. Coomassie blue–stained gels used as loading control and Lamin B used as a control for equal loading of the samples. Data represent as mean ± SEM. Compared to con, ** p < 0.01, ***p < 0.001, **** p < 0.0001; compared to a concentration of 0 μM, ###p < 0.001, ####p < 0.0001 and ns: not significant. Supplementary Figure 3. CTG12 impedes the priming process of NLRP3 inflammasome activation and specifically inhibits canonical and noncanonical NLRP3 inflammasome activation.BMDMs were primed with LPS treated with CTG12, then stimulated with Nigericin ATP, poly, or SiO₂. Supernatants were collected for the measurement of TNF-α, BMDMs primed with Pam3CSK4 treated with CTG12, followed by cytosolic LPS. Supernatants were collected fo [file 12964_2026_2741_MOESM1_ESM.zip › supplementary file/Figure3-Supplementary Figure3原膜/Figure3-A-caspase1 p20.png]

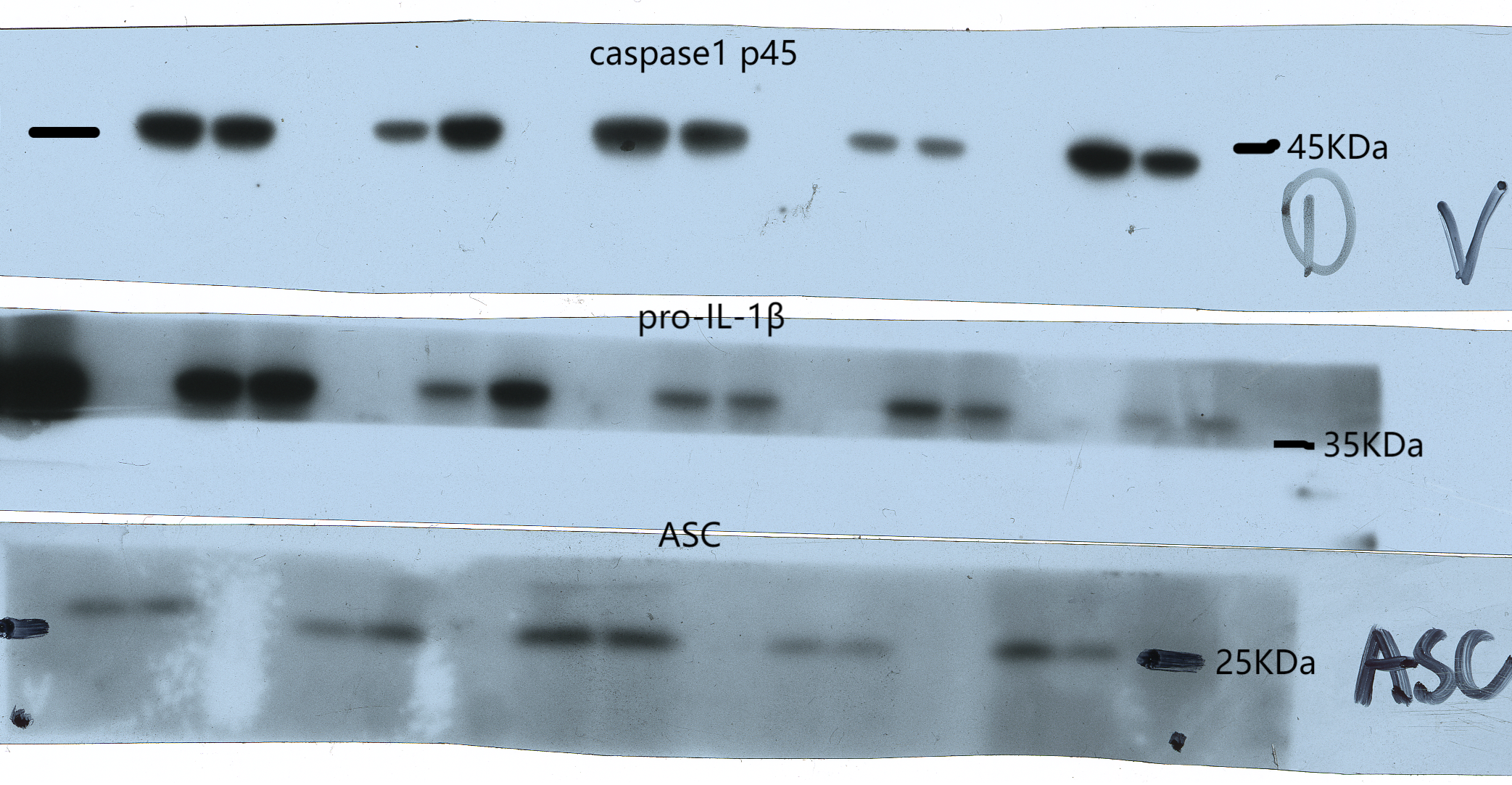

Supplement: Supplementary file 1 — Supplementary Material 1. Supplementary Figure 1. Licochalcone B derivatives inhibit NLRP3 inflammasome activation.BMDMs were primed with LPS for 4 hours, treated with Echinatin, Licorice Chalcone B, CTG4, CTG1, CTG10, CTG11, CTG12, CTG13, CTG14, CTG15, CTG16, CTG18, CTG19, CAPE, CTG23for 30 minutes, and then stimulated with nigericin for 25 minutes. Supernatants were collected for the measurement of caspase-1. Data represent as mean ± SEM. Compared to con, **** p < 0.0001; compared to a concentration of 0 μM, ###p < 0.001, #### p < 0.0001 and ns:not significant. Supplementary Figure 2. CTG11 and CTG13 inhibit NLRP3 inflammasome activation in mouse BMDMs.The structure of CTG11.Western blot analysis of IL-1β, caspase-1in culture supernatantsand pro-IL-1β, caspase-1, NLRP3, ASC in whole cell lysatesof LPS-primed BMDMs treated with CTG11 and then stimulated with Nigericin, supernatants were collected for the measurement of caspase-1, IL-1β, LDHand TNF-α.The structure of CTG13.Western blot analysis of IL-1β, caspase-1in culture supernatantsand pro-IL-1β, caspase-1, NLRP3, ASC in whole cell lysatesof LPS-primed BMDMs treated with CTG13 and then stimulated with Nigericin, supernatants were collected for the measurement of caspase-1, IL-1β, LDHand TNF-α. Coomassie blue–stained gels used as loading control and Lamin B used as a control for equal loading of the samples. Data represent as mean ± SEM. Compared to con, ** p < 0.01, ***p < 0.001, **** p < 0.0001; compared to a concentration of 0 μM, ###p < 0.001, ####p < 0.0001 and ns: not significant. Supplementary Figure 3. CTG12 impedes the priming process of NLRP3 inflammasome activation and specifically inhibits canonical and noncanonical NLRP3 inflammasome activation.BMDMs were primed with LPS treated with CTG12, then stimulated with Nigericin ATP, poly, or SiO₂. Supernatants were collected for the measurement of TNF-α, BMDMs primed with Pam3CSK4 treated with CTG12, followed by cytosolic LPS. Supernatants were collected fo [file 12964_2026_2741_MOESM1_ESM.zip › supplementary file/Figure3-Supplementary Figure3原膜/Figure3-A-caspase1 p45-pro-IL-1β-ASC.png]

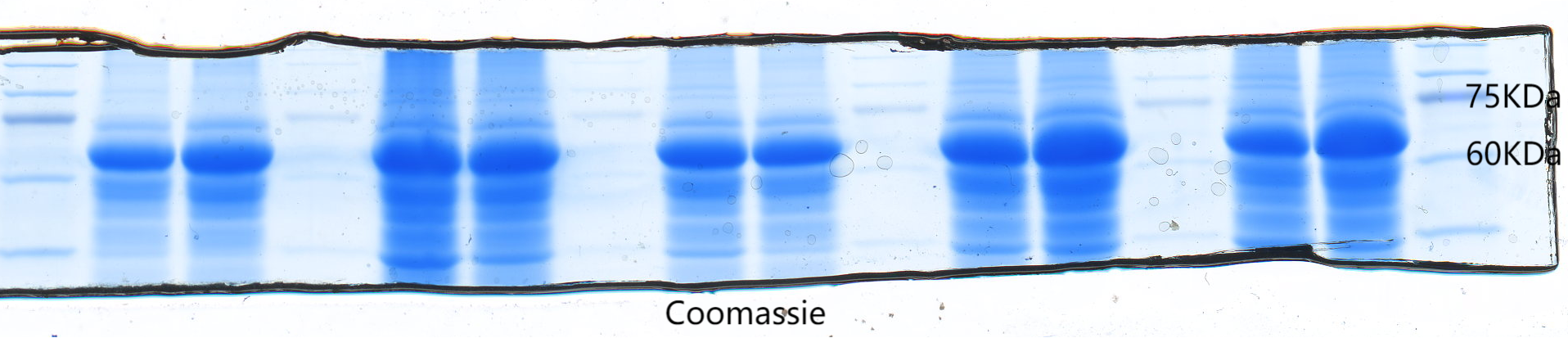

Supplement: Supplementary file 1 — Supplementary Material 1. Supplementary Figure 1. Licochalcone B derivatives inhibit NLRP3 inflammasome activation.BMDMs were primed with LPS for 4 hours, treated with Echinatin, Licorice Chalcone B, CTG4, CTG1, CTG10, CTG11, CTG12, CTG13, CTG14, CTG15, CTG16, CTG18, CTG19, CAPE, CTG23for 30 minutes, and then stimulated with nigericin for 25 minutes. Supernatants were collected for the measurement of caspase-1. Data represent as mean ± SEM. Compared to con, **** p < 0.0001; compared to a concentration of 0 μM, ###p < 0.001, #### p < 0.0001 and ns:not significant. Supplementary Figure 2. CTG11 and CTG13 inhibit NLRP3 inflammasome activation in mouse BMDMs.The structure of CTG11.Western blot analysis of IL-1β, caspase-1in culture supernatantsand pro-IL-1β, caspase-1, NLRP3, ASC in whole cell lysatesof LPS-primed BMDMs treated with CTG11 and then stimulated with Nigericin, supernatants were collected for the measurement of caspase-1, IL-1β, LDHand TNF-α.The structure of CTG13.Western blot analysis of IL-1β, caspase-1in culture supernatantsand pro-IL-1β, caspase-1, NLRP3, ASC in whole cell lysatesof LPS-primed BMDMs treated with CTG13 and then stimulated with Nigericin, supernatants were collected for the measurement of caspase-1, IL-1β, LDHand TNF-α. Coomassie blue–stained gels used as loading control and Lamin B used as a control for equal loading of the samples. Data represent as mean ± SEM. Compared to con, ** p < 0.01, ***p < 0.001, **** p < 0.0001; compared to a concentration of 0 μM, ###p < 0.001, ####p < 0.0001 and ns: not significant. Supplementary Figure 3. CTG12 impedes the priming process of NLRP3 inflammasome activation and specifically inhibits canonical and noncanonical NLRP3 inflammasome activation.BMDMs were primed with LPS treated with CTG12, then stimulated with Nigericin ATP, poly, or SiO₂. Supernatants were collected for the measurement of TNF-α, BMDMs primed with Pam3CSK4 treated with CTG12, followed by cytosolic LPS. Supernatants were collected fo [file 12964_2026_2741_MOESM1_ESM.zip › supplementary file/Figure3-Supplementary Figure3原膜/Figure3-A-Coomassie条带.png]

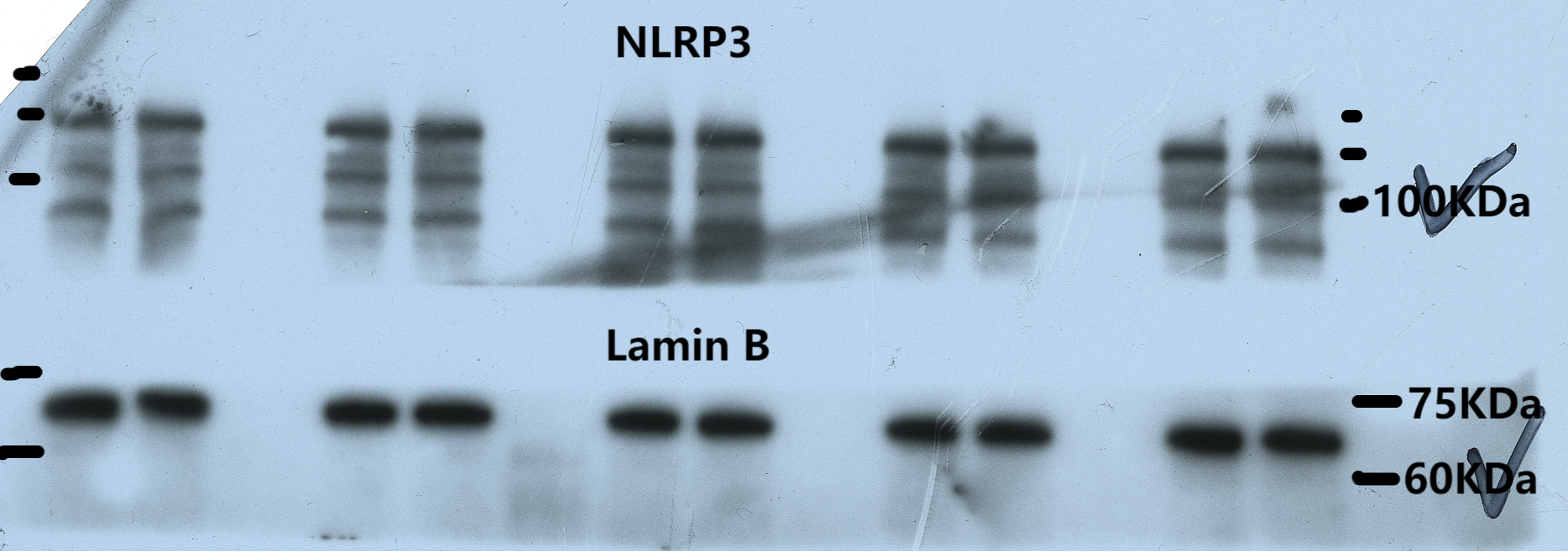

Supplement: Supplementary file 1 — Supplementary Material 1. Supplementary Figure 1. Licochalcone B derivatives inhibit NLRP3 inflammasome activation.BMDMs were primed with LPS for 4 hours, treated with Echinatin, Licorice Chalcone B, CTG4, CTG1, CTG10, CTG11, CTG12, CTG13, CTG14, CTG15, CTG16, CTG18, CTG19, CAPE, CTG23for 30 minutes, and then stimulated with nigericin for 25 minutes. Supernatants were collected for the measurement of caspase-1. Data represent as mean ± SEM. Compared to con, **** p < 0.0001; compared to a concentration of 0 μM, ###p < 0.001, #### p < 0.0001 and ns:not significant. Supplementary Figure 2. CTG11 and CTG13 inhibit NLRP3 inflammasome activation in mouse BMDMs.The structure of CTG11.Western blot analysis of IL-1β, caspase-1in culture supernatantsand pro-IL-1β, caspase-1, NLRP3, ASC in whole cell lysatesof LPS-primed BMDMs treated with CTG11 and then stimulated with Nigericin, supernatants were collected for the measurement of caspase-1, IL-1β, LDHand TNF-α.The structure of CTG13.Western blot analysis of IL-1β, caspase-1in culture supernatantsand pro-IL-1β, caspase-1, NLRP3, ASC in whole cell lysatesof LPS-primed BMDMs treated with CTG13 and then stimulated with Nigericin, supernatants were collected for the measurement of caspase-1, IL-1β, LDHand TNF-α. Coomassie blue–stained gels used as loading control and Lamin B used as a control for equal loading of the samples. Data represent as mean ± SEM. Compared to con, ** p < 0.01, ***p < 0.001, **** p < 0.0001; compared to a concentration of 0 μM, ###p < 0.001, ####p < 0.0001 and ns: not significant. Supplementary Figure 3. CTG12 impedes the priming process of NLRP3 inflammasome activation and specifically inhibits canonical and noncanonical NLRP3 inflammasome activation.BMDMs were primed with LPS treated with CTG12, then stimulated with Nigericin ATP, poly, or SiO₂. Supernatants were collected for the measurement of TNF-α, BMDMs primed with Pam3CSK4 treated with CTG12, followed by cytosolic LPS. Supernatants were collected fo [file 12964_2026_2741_MOESM1_ESM.zip › supplementary file/Figure3-Supplementary Figure3原膜/Figure3-A-NLRP3-Lamin B.png]

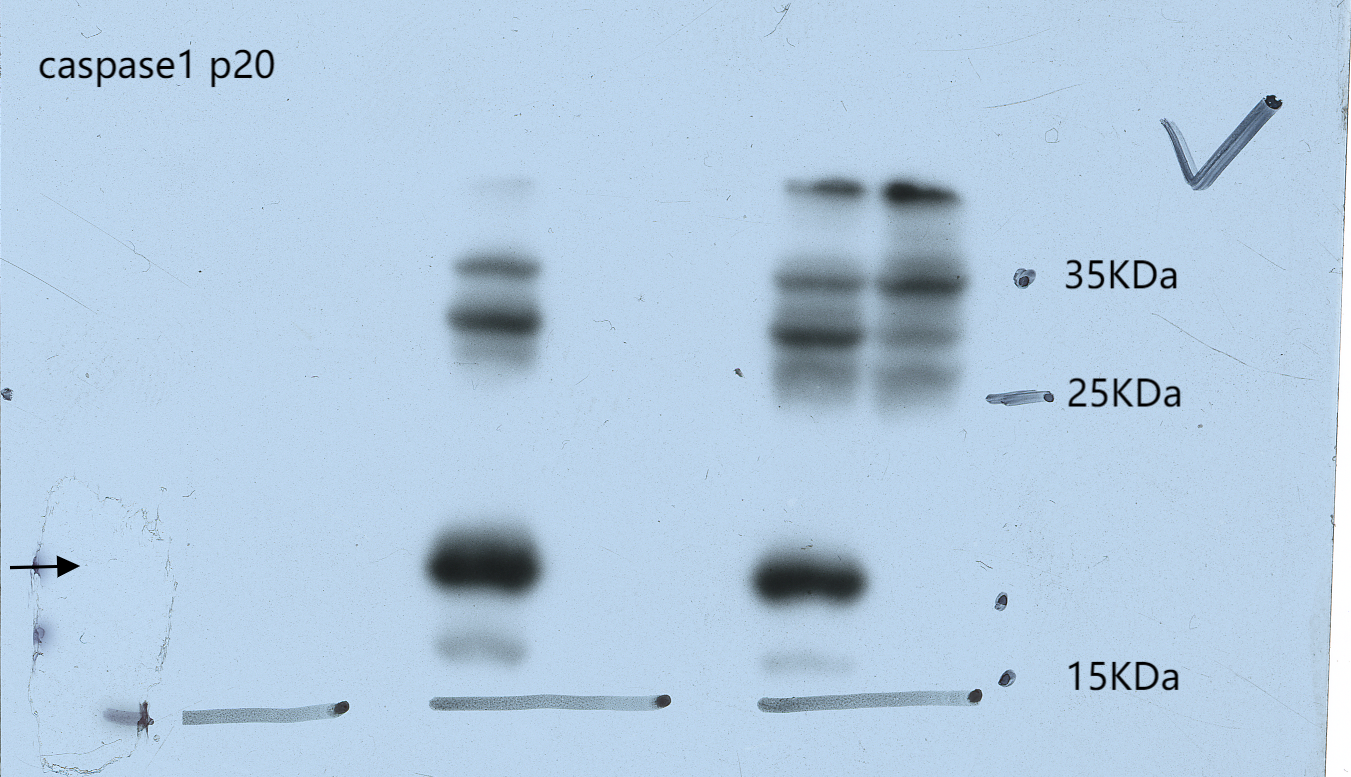

Supplement: Supplementary file 1 — Supplementary Material 1. Supplementary Figure 1. Licochalcone B derivatives inhibit NLRP3 inflammasome activation.BMDMs were primed with LPS for 4 hours, treated with Echinatin, Licorice Chalcone B, CTG4, CTG1, CTG10, CTG11, CTG12, CTG13, CTG14, CTG15, CTG16, CTG18, CTG19, CAPE, CTG23for 30 minutes, and then stimulated with nigericin for 25 minutes. Supernatants were collected for the measurement of caspase-1. Data represent as mean ± SEM. Compared to con, **** p < 0.0001; compared to a concentration of 0 μM, ###p < 0.001, #### p < 0.0001 and ns:not significant. Supplementary Figure 2. CTG11 and CTG13 inhibit NLRP3 inflammasome activation in mouse BMDMs.The structure of CTG11.Western blot analysis of IL-1β, caspase-1in culture supernatantsand pro-IL-1β, caspase-1, NLRP3, ASC in whole cell lysatesof LPS-primed BMDMs treated with CTG11 and then stimulated with Nigericin, supernatants were collected for the measurement of caspase-1, IL-1β, LDHand TNF-α.The structure of CTG13.Western blot analysis of IL-1β, caspase-1in culture supernatantsand pro-IL-1β, caspase-1, NLRP3, ASC in whole cell lysatesof LPS-primed BMDMs treated with CTG13 and then stimulated with Nigericin, supernatants were collected for the measurement of caspase-1, IL-1β, LDHand TNF-α. Coomassie blue–stained gels used as loading control and Lamin B used as a control for equal loading of the samples. Data represent as mean ± SEM. Compared to con, ** p < 0.01, ***p < 0.001, **** p < 0.0001; compared to a concentration of 0 μM, ###p < 0.001, ####p < 0.0001 and ns: not significant. Supplementary Figure 3. CTG12 impedes the priming process of NLRP3 inflammasome activation and specifically inhibits canonical and noncanonical NLRP3 inflammasome activation.BMDMs were primed with LPS treated with CTG12, then stimulated with Nigericin ATP, poly, or SiO₂. Supernatants were collected for the measurement of TNF-α, BMDMs primed with Pam3CSK4 treated with CTG12, followed by cytosolic LPS. Supernatants were collected fo [file 12964_2026_2741_MOESM1_ESM.zip › supplementary file/Figure3-Supplementary Figure3原膜/Figure3-D-caspase1 p20.png]

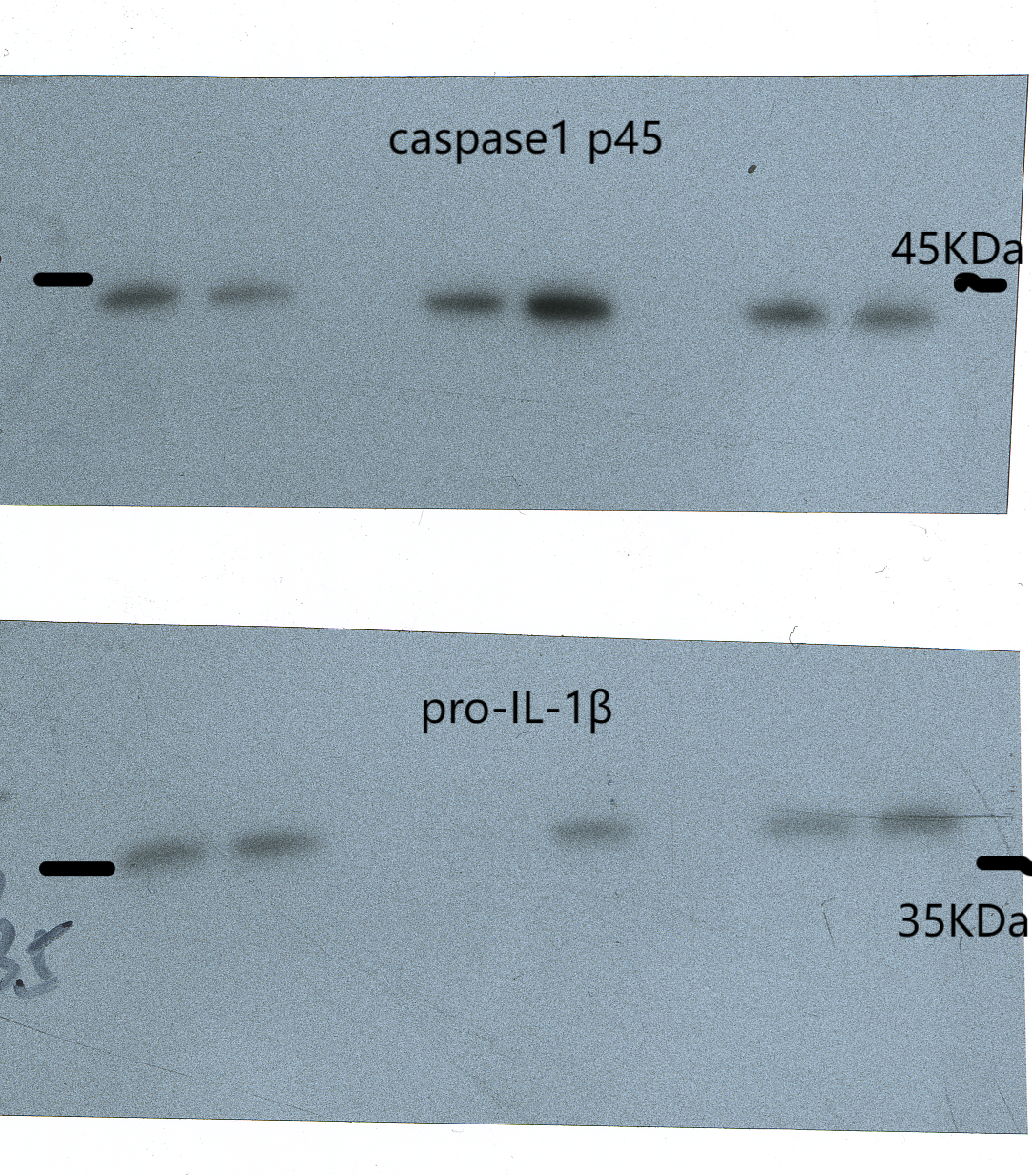

Supplement: Supplementary file 1 — Supplementary Material 1. Supplementary Figure 1. Licochalcone B derivatives inhibit NLRP3 inflammasome activation.BMDMs were primed with LPS for 4 hours, treated with Echinatin, Licorice Chalcone B, CTG4, CTG1, CTG10, CTG11, CTG12, CTG13, CTG14, CTG15, CTG16, CTG18, CTG19, CAPE, CTG23for 30 minutes, and then stimulated with nigericin for 25 minutes. Supernatants were collected for the measurement of caspase-1. Data represent as mean ± SEM. Compared to con, **** p < 0.0001; compared to a concentration of 0 μM, ###p < 0.001, #### p < 0.0001 and ns:not significant. Supplementary Figure 2. CTG11 and CTG13 inhibit NLRP3 inflammasome activation in mouse BMDMs.The structure of CTG11.Western blot analysis of IL-1β, caspase-1in culture supernatantsand pro-IL-1β, caspase-1, NLRP3, ASC in whole cell lysatesof LPS-primed BMDMs treated with CTG11 and then stimulated with Nigericin, supernatants were collected for the measurement of caspase-1, IL-1β, LDHand TNF-α.The structure of CTG13.Western blot analysis of IL-1β, caspase-1in culture supernatantsand pro-IL-1β, caspase-1, NLRP3, ASC in whole cell lysatesof LPS-primed BMDMs treated with CTG13 and then stimulated with Nigericin, supernatants were collected for the measurement of caspase-1, IL-1β, LDHand TNF-α. Coomassie blue–stained gels used as loading control and Lamin B used as a control for equal loading of the samples. Data represent as mean ± SEM. Compared to con, ** p < 0.01, ***p < 0.001, **** p < 0.0001; compared to a concentration of 0 μM, ###p < 0.001, ####p < 0.0001 and ns: not significant. Supplementary Figure 3. CTG12 impedes the priming process of NLRP3 inflammasome activation and specifically inhibits canonical and noncanonical NLRP3 inflammasome activation.BMDMs were primed with LPS treated with CTG12, then stimulated with Nigericin ATP, poly, or SiO₂. Supernatants were collected for the measurement of TNF-α, BMDMs primed with Pam3CSK4 treated with CTG12, followed by cytosolic LPS. Supernatants were collected fo [file 12964_2026_2741_MOESM1_ESM.zip › supplementary file/Figure3-Supplementary Figure3原膜/Figure3-D-caspase1 p45-pro-IL-1β.png]

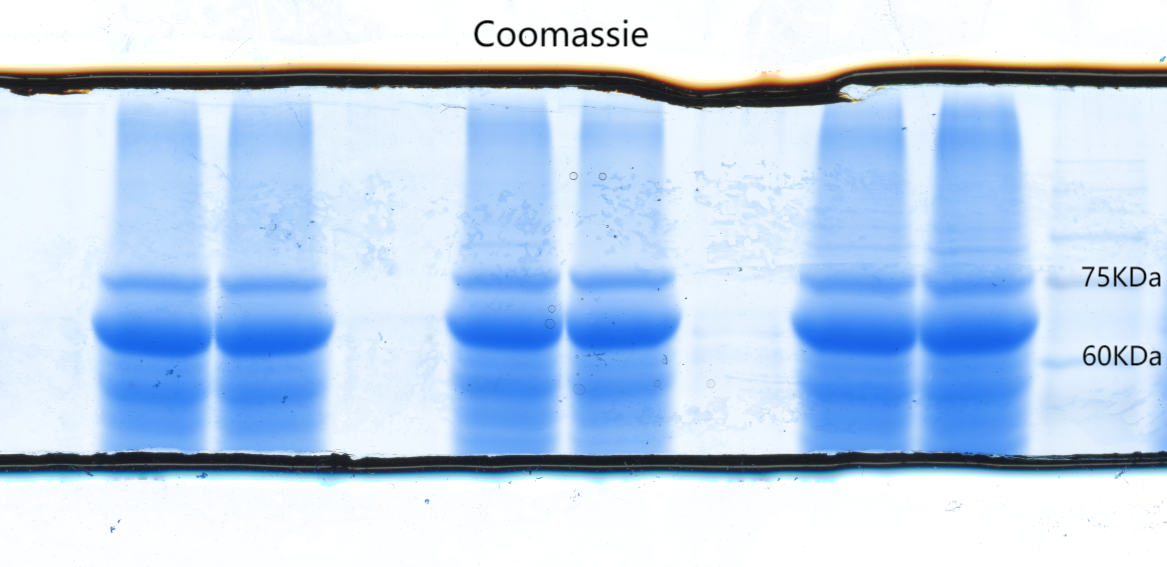

Supplement: Supplementary file 1 — Supplementary Material 1. Supplementary Figure 1. Licochalcone B derivatives inhibit NLRP3 inflammasome activation.BMDMs were primed with LPS for 4 hours, treated with Echinatin, Licorice Chalcone B, CTG4, CTG1, CTG10, CTG11, CTG12, CTG13, CTG14, CTG15, CTG16, CTG18, CTG19, CAPE, CTG23for 30 minutes, and then stimulated with nigericin for 25 minutes. Supernatants were collected for the measurement of caspase-1. Data represent as mean ± SEM. Compared to con, **** p < 0.0001; compared to a concentration of 0 μM, ###p < 0.001, #### p < 0.0001 and ns:not significant. Supplementary Figure 2. CTG11 and CTG13 inhibit NLRP3 inflammasome activation in mouse BMDMs.The structure of CTG11.Western blot analysis of IL-1β, caspase-1in culture supernatantsand pro-IL-1β, caspase-1, NLRP3, ASC in whole cell lysatesof LPS-primed BMDMs treated with CTG11 and then stimulated with Nigericin, supernatants were collected for the measurement of caspase-1, IL-1β, LDHand TNF-α.The structure of CTG13.Western blot analysis of IL-1β, caspase-1in culture supernatantsand pro-IL-1β, caspase-1, NLRP3, ASC in whole cell lysatesof LPS-primed BMDMs treated with CTG13 and then stimulated with Nigericin, supernatants were collected for the measurement of caspase-1, IL-1β, LDHand TNF-α. Coomassie blue–stained gels used as loading control and Lamin B used as a control for equal loading of the samples. Data represent as mean ± SEM. Compared to con, ** p < 0.01, ***p < 0.001, **** p < 0.0001; compared to a concentration of 0 μM, ###p < 0.001, ####p < 0.0001 and ns: not significant. Supplementary Figure 3. CTG12 impedes the priming process of NLRP3 inflammasome activation and specifically inhibits canonical and noncanonical NLRP3 inflammasome activation.BMDMs were primed with LPS treated with CTG12, then stimulated with Nigericin ATP, poly, or SiO₂. Supernatants were collected for the measurement of TNF-α, BMDMs primed with Pam3CSK4 treated with CTG12, followed by cytosolic LPS. Supernatants were collected fo [file 12964_2026_2741_MOESM1_ESM.zip › supplementary file/Figure3-Supplementary Figure3原膜/Figure3-D-Coomassie条带.png]

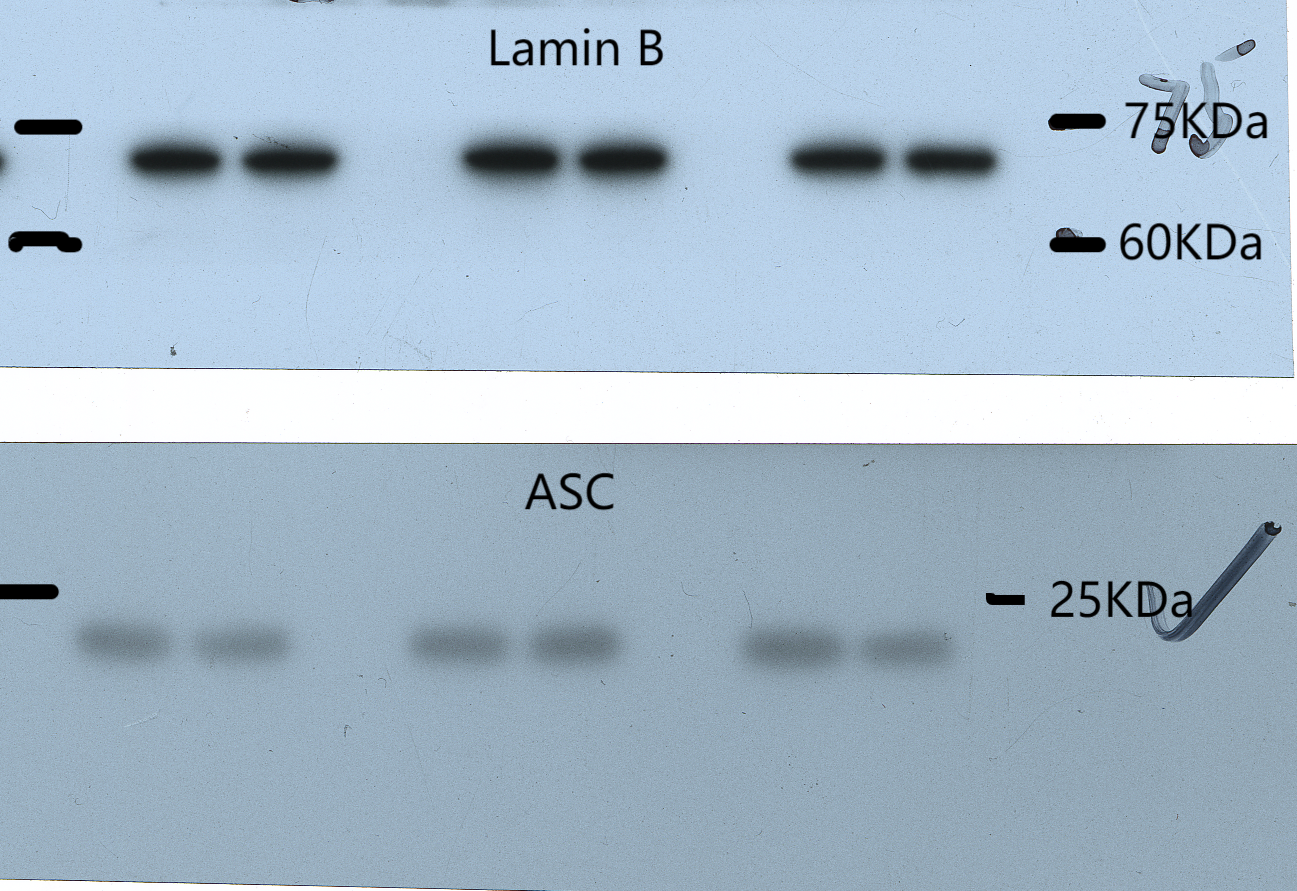

Supplement: Supplementary file 1 — Supplementary Material 1. Supplementary Figure 1. Licochalcone B derivatives inhibit NLRP3 inflammasome activation.BMDMs were primed with LPS for 4 hours, treated with Echinatin, Licorice Chalcone B, CTG4, CTG1, CTG10, CTG11, CTG12, CTG13, CTG14, CTG15, CTG16, CTG18, CTG19, CAPE, CTG23for 30 minutes, and then stimulated with nigericin for 25 minutes. Supernatants were collected for the measurement of caspase-1. Data represent as mean ± SEM. Compared to con, **** p < 0.0001; compared to a concentration of 0 μM, ###p < 0.001, #### p < 0.0001 and ns:not significant. Supplementary Figure 2. CTG11 and CTG13 inhibit NLRP3 inflammasome activation in mouse BMDMs.The structure of CTG11.Western blot analysis of IL-1β, caspase-1in culture supernatantsand pro-IL-1β, caspase-1, NLRP3, ASC in whole cell lysatesof LPS-primed BMDMs treated with CTG11 and then stimulated with Nigericin, supernatants were collected for the measurement of caspase-1, IL-1β, LDHand TNF-α.The structure of CTG13.Western blot analysis of IL-1β, caspase-1in culture supernatantsand pro-IL-1β, caspase-1, NLRP3, ASC in whole cell lysatesof LPS-primed BMDMs treated with CTG13 and then stimulated with Nigericin, supernatants were collected for the measurement of caspase-1, IL-1β, LDHand TNF-α. Coomassie blue–stained gels used as loading control and Lamin B used as a control for equal loading of the samples. Data represent as mean ± SEM. Compared to con, ** p < 0.01, ***p < 0.001, **** p < 0.0001; compared to a concentration of 0 μM, ###p < 0.001, ####p < 0.0001 and ns: not significant. Supplementary Figure 3. CTG12 impedes the priming process of NLRP3 inflammasome activation and specifically inhibits canonical and noncanonical NLRP3 inflammasome activation.BMDMs were primed with LPS treated with CTG12, then stimulated with Nigericin ATP, poly, or SiO₂. Supernatants were collected for the measurement of TNF-α, BMDMs primed with Pam3CSK4 treated with CTG12, followed by cytosolic LPS. Supernatants were collected fo [file 12964_2026_2741_MOESM1_ESM.zip › supplementary file/Figure3-Supplementary Figure3原膜/Figure3-D-Lamin B-ASC.png]

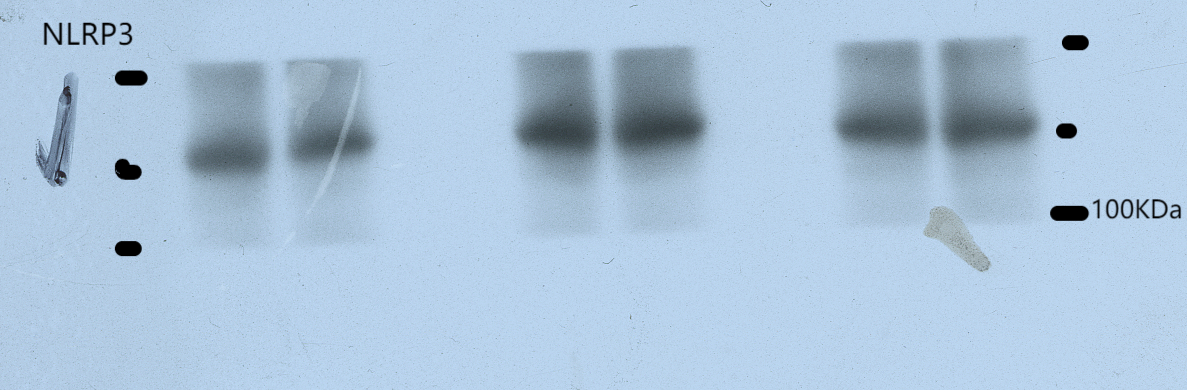

Supplement: Supplementary file 1 — Supplementary Material 1. Supplementary Figure 1. Licochalcone B derivatives inhibit NLRP3 inflammasome activation.BMDMs were primed with LPS for 4 hours, treated with Echinatin, Licorice Chalcone B, CTG4, CTG1, CTG10, CTG11, CTG12, CTG13, CTG14, CTG15, CTG16, CTG18, CTG19, CAPE, CTG23for 30 minutes, and then stimulated with nigericin for 25 minutes. Supernatants were collected for the measurement of caspase-1. Data represent as mean ± SEM. Compared to con, **** p < 0.0001; compared to a concentration of 0 μM, ###p < 0.001, #### p < 0.0001 and ns:not significant. Supplementary Figure 2. CTG11 and CTG13 inhibit NLRP3 inflammasome activation in mouse BMDMs.The structure of CTG11.Western blot analysis of IL-1β, caspase-1in culture supernatantsand pro-IL-1β, caspase-1, NLRP3, ASC in whole cell lysatesof LPS-primed BMDMs treated with CTG11 and then stimulated with Nigericin, supernatants were collected for the measurement of caspase-1, IL-1β, LDHand TNF-α.The structure of CTG13.Western blot analysis of IL-1β, caspase-1in culture supernatantsand pro-IL-1β, caspase-1, NLRP3, ASC in whole cell lysatesof LPS-primed BMDMs treated with CTG13 and then stimulated with Nigericin, supernatants were collected for the measurement of caspase-1, IL-1β, LDHand TNF-α. Coomassie blue–stained gels used as loading control and Lamin B used as a control for equal loading of the samples. Data represent as mean ± SEM. Compared to con, ** p < 0.01, ***p < 0.001, **** p < 0.0001; compared to a concentration of 0 μM, ###p < 0.001, ####p < 0.0001 and ns: not significant. Supplementary Figure 3. CTG12 impedes the priming process of NLRP3 inflammasome activation and specifically inhibits canonical and noncanonical NLRP3 inflammasome activation.BMDMs were primed with LPS treated with CTG12, then stimulated with Nigericin ATP, poly, or SiO₂. Supernatants were collected for the measurement of TNF-α, BMDMs primed with Pam3CSK4 treated with CTG12, followed by cytosolic LPS. Supernatants were collected fo [file 12964_2026_2741_MOESM1_ESM.zip › supplementary file/Figure3-Supplementary Figure3原膜/Figure3-D-NLRP3.png]

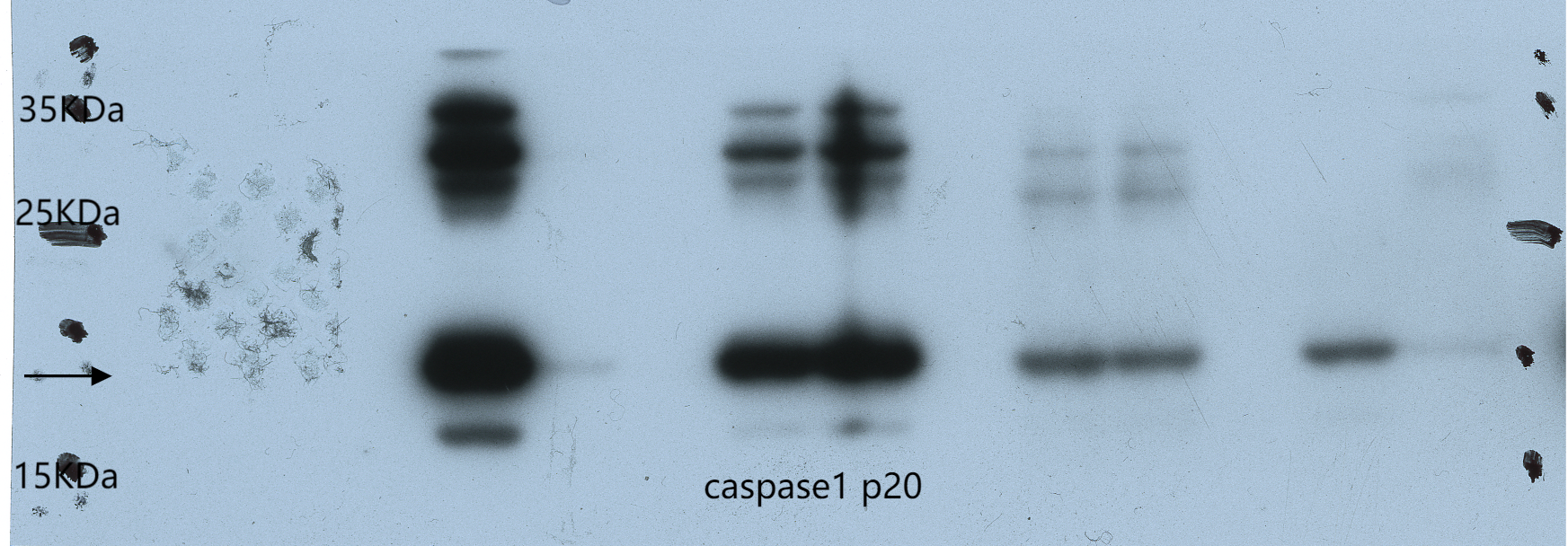

Supplement: Supplementary file 1 — Supplementary Material 1. Supplementary Figure 1. Licochalcone B derivatives inhibit NLRP3 inflammasome activation.BMDMs were primed with LPS for 4 hours, treated with Echinatin, Licorice Chalcone B, CTG4, CTG1, CTG10, CTG11, CTG12, CTG13, CTG14, CTG15, CTG16, CTG18, CTG19, CAPE, CTG23for 30 minutes, and then stimulated with nigericin for 25 minutes. Supernatants were collected for the measurement of caspase-1. Data represent as mean ± SEM. Compared to con, **** p < 0.0001; compared to a concentration of 0 μM, ###p < 0.001, #### p < 0.0001 and ns:not significant. Supplementary Figure 2. CTG11 and CTG13 inhibit NLRP3 inflammasome activation in mouse BMDMs.The structure of CTG11.Western blot analysis of IL-1β, caspase-1in culture supernatantsand pro-IL-1β, caspase-1, NLRP3, ASC in whole cell lysatesof LPS-primed BMDMs treated with CTG11 and then stimulated with Nigericin, supernatants were collected for the measurement of caspase-1, IL-1β, LDHand TNF-α.The structure of CTG13.Western blot analysis of IL-1β, caspase-1in culture supernatantsand pro-IL-1β, caspase-1, NLRP3, ASC in whole cell lysatesof LPS-primed BMDMs treated with CTG13 and then stimulated with Nigericin, supernatants were collected for the measurement of caspase-1, IL-1β, LDHand TNF-α. Coomassie blue–stained gels used as loading control and Lamin B used as a control for equal loading of the samples. Data represent as mean ± SEM. Compared to con, ** p < 0.01, ***p < 0.001, **** p < 0.0001; compared to a concentration of 0 μM, ###p < 0.001, ####p < 0.0001 and ns: not significant. Supplementary Figure 3. CTG12 impedes the priming process of NLRP3 inflammasome activation and specifically inhibits canonical and noncanonical NLRP3 inflammasome activation.BMDMs were primed with LPS treated with CTG12, then stimulated with Nigericin ATP, poly, or SiO₂. Supernatants were collected for the measurement of TNF-α, BMDMs primed with Pam3CSK4 treated with CTG12, followed by cytosolic LPS. Supernatants were collected fo [file 12964_2026_2741_MOESM1_ESM.zip › supplementary file/Figure3-Supplementary Figure3原膜/Figure3-G-caspase1 p20.png]

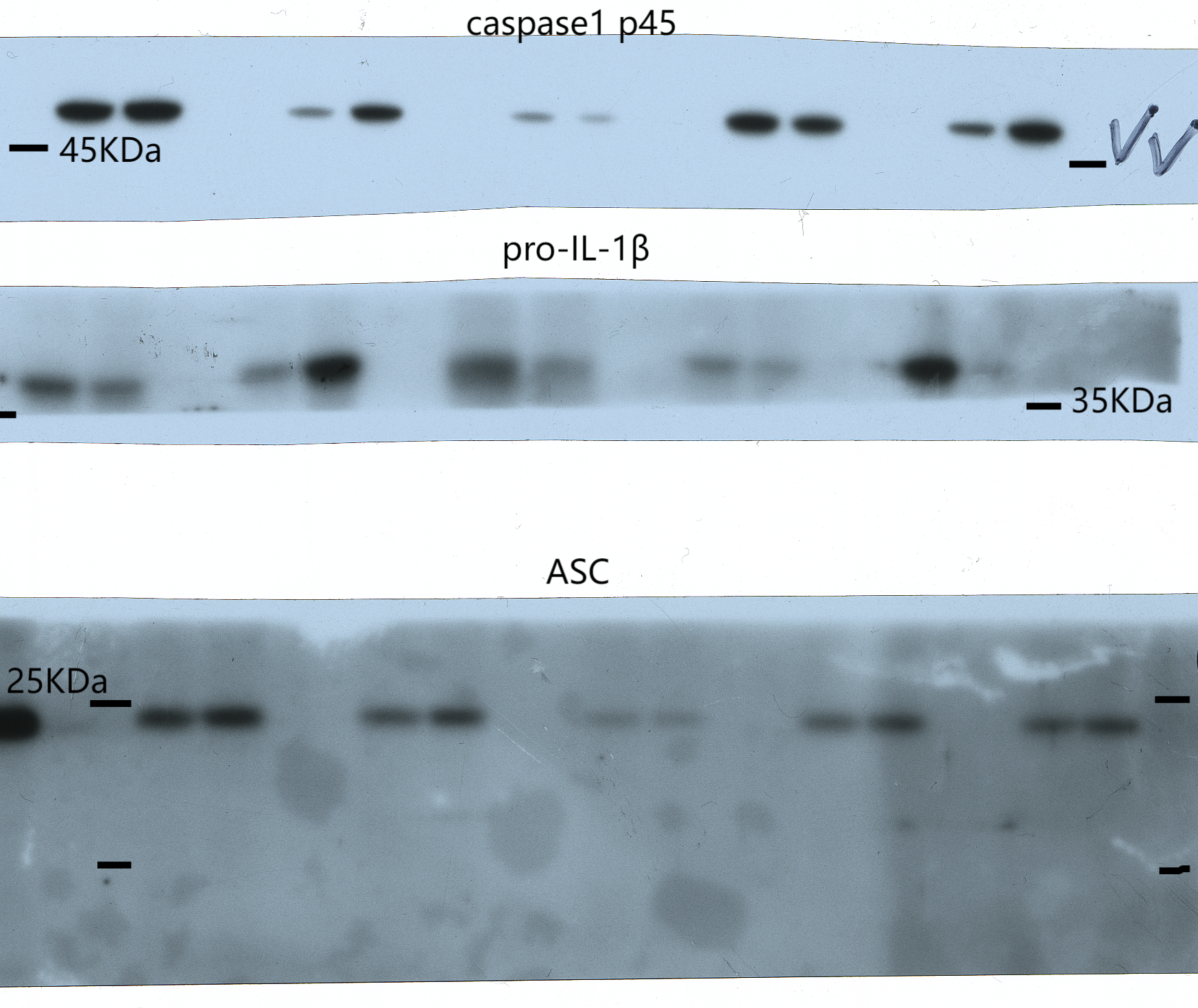

Supplement: Supplementary file 1 — Supplementary Material 1. Supplementary Figure 1. Licochalcone B derivatives inhibit NLRP3 inflammasome activation.BMDMs were primed with LPS for 4 hours, treated with Echinatin, Licorice Chalcone B, CTG4, CTG1, CTG10, CTG11, CTG12, CTG13, CTG14, CTG15, CTG16, CTG18, CTG19, CAPE, CTG23for 30 minutes, and then stimulated with nigericin for 25 minutes. Supernatants were collected for the measurement of caspase-1. Data represent as mean ± SEM. Compared to con, **** p < 0.0001; compared to a concentration of 0 μM, ###p < 0.001, #### p < 0.0001 and ns:not significant. Supplementary Figure 2. CTG11 and CTG13 inhibit NLRP3 inflammasome activation in mouse BMDMs.The structure of CTG11.Western blot analysis of IL-1β, caspase-1in culture supernatantsand pro-IL-1β, caspase-1, NLRP3, ASC in whole cell lysatesof LPS-primed BMDMs treated with CTG11 and then stimulated with Nigericin, supernatants were collected for the measurement of caspase-1, IL-1β, LDHand TNF-α.The structure of CTG13.Western blot analysis of IL-1β, caspase-1in culture supernatantsand pro-IL-1β, caspase-1, NLRP3, ASC in whole cell lysatesof LPS-primed BMDMs treated with CTG13 and then stimulated with Nigericin, supernatants were collected for the measurement of caspase-1, IL-1β, LDHand TNF-α. Coomassie blue–stained gels used as loading control and Lamin B used as a control for equal loading of the samples. Data represent as mean ± SEM. Compared to con, ** p < 0.01, ***p < 0.001, **** p < 0.0001; compared to a concentration of 0 μM, ###p < 0.001, ####p < 0.0001 and ns: not significant. Supplementary Figure 3. CTG12 impedes the priming process of NLRP3 inflammasome activation and specifically inhibits canonical and noncanonical NLRP3 inflammasome activation.BMDMs were primed with LPS treated with CTG12, then stimulated with Nigericin ATP, poly, or SiO₂. Supernatants were collected for the measurement of TNF-α, BMDMs primed with Pam3CSK4 treated with CTG12, followed by cytosolic LPS. Supernatants were collected fo [file 12964_2026_2741_MOESM1_ESM.zip › supplementary file/Figure3-Supplementary Figure3原膜/Figure3-G-caspase1 p45-pro-IL-1β-ASC.png]

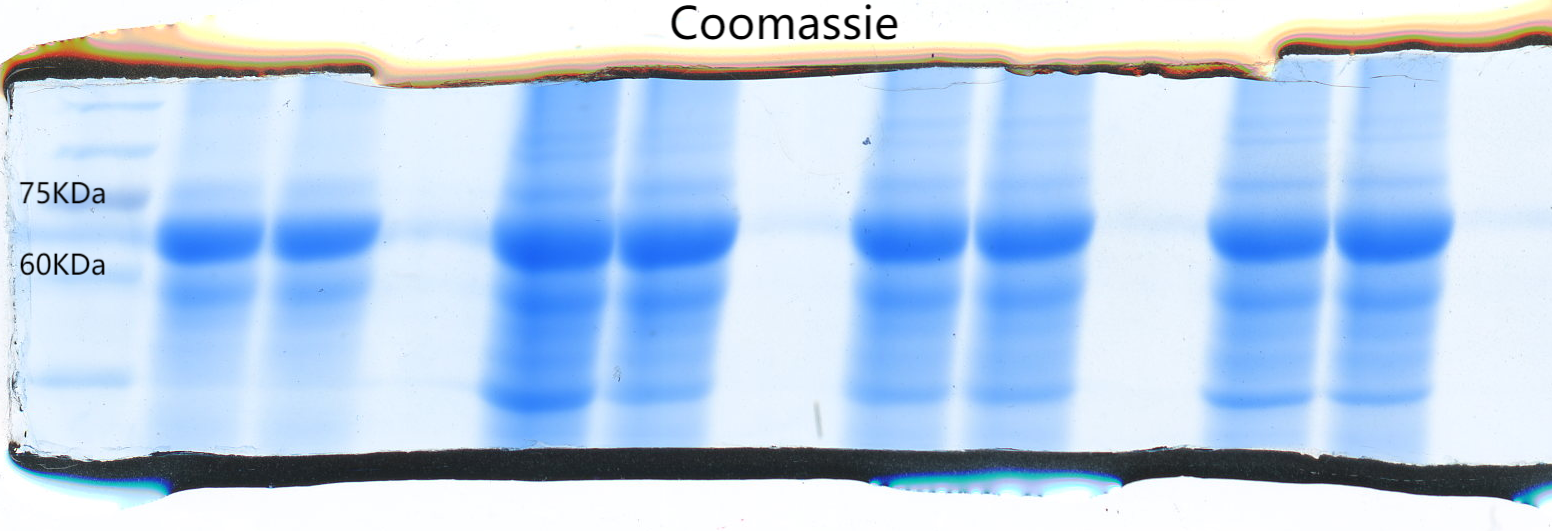

Supplement: Supplementary file 1 — Supplementary Material 1. Supplementary Figure 1. Licochalcone B derivatives inhibit NLRP3 inflammasome activation.BMDMs were primed with LPS for 4 hours, treated with Echinatin, Licorice Chalcone B, CTG4, CTG1, CTG10, CTG11, CTG12, CTG13, CTG14, CTG15, CTG16, CTG18, CTG19, CAPE, CTG23for 30 minutes, and then stimulated with nigericin for 25 minutes. Supernatants were collected for the measurement of caspase-1. Data represent as mean ± SEM. Compared to con, **** p < 0.0001; compared to a concentration of 0 μM, ###p < 0.001, #### p < 0.0001 and ns:not significant. Supplementary Figure 2. CTG11 and CTG13 inhibit NLRP3 inflammasome activation in mouse BMDMs.The structure of CTG11.Western blot analysis of IL-1β, caspase-1in culture supernatantsand pro-IL-1β, caspase-1, NLRP3, ASC in whole cell lysatesof LPS-primed BMDMs treated with CTG11 and then stimulated with Nigericin, supernatants were collected for the measurement of caspase-1, IL-1β, LDHand TNF-α.The structure of CTG13.Western blot analysis of IL-1β, caspase-1in culture supernatantsand pro-IL-1β, caspase-1, NLRP3, ASC in whole cell lysatesof LPS-primed BMDMs treated with CTG13 and then stimulated with Nigericin, supernatants were collected for the measurement of caspase-1, IL-1β, LDHand TNF-α. Coomassie blue–stained gels used as loading control and Lamin B used as a control for equal loading of the samples. Data represent as mean ± SEM. Compared to con, ** p < 0.01, ***p < 0.001, **** p < 0.0001; compared to a concentration of 0 μM, ###p < 0.001, ####p < 0.0001 and ns: not significant. Supplementary Figure 3. CTG12 impedes the priming process of NLRP3 inflammasome activation and specifically inhibits canonical and noncanonical NLRP3 inflammasome activation.BMDMs were primed with LPS treated with CTG12, then stimulated with Nigericin ATP, poly, or SiO₂. Supernatants were collected for the measurement of TNF-α, BMDMs primed with Pam3CSK4 treated with CTG12, followed by cytosolic LPS. Supernatants were collected fo [file 12964_2026_2741_MOESM1_ESM.zip › supplementary file/Figure3-Supplementary Figure3原膜/Figure3-G-Coomassie条带.png]

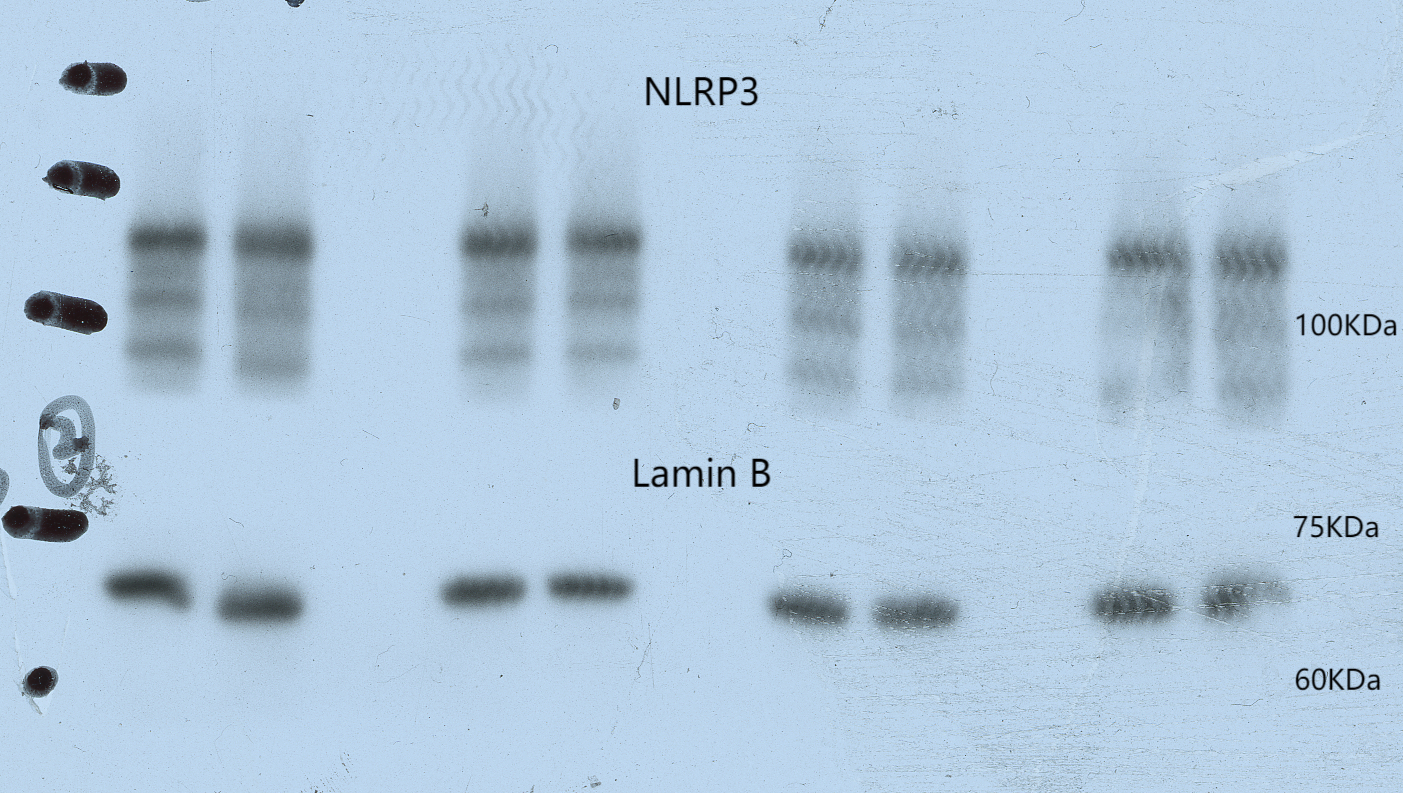

Supplement: Supplementary file 1 — Supplementary Material 1. Supplementary Figure 1. Licochalcone B derivatives inhibit NLRP3 inflammasome activation.BMDMs were primed with LPS for 4 hours, treated with Echinatin, Licorice Chalcone B, CTG4, CTG1, CTG10, CTG11, CTG12, CTG13, CTG14, CTG15, CTG16, CTG18, CTG19, CAPE, CTG23for 30 minutes, and then stimulated with nigericin for 25 minutes. Supernatants were collected for the measurement of caspase-1. Data represent as mean ± SEM. Compared to con, **** p < 0.0001; compared to a concentration of 0 μM, ###p < 0.001, #### p < 0.0001 and ns:not significant. Supplementary Figure 2. CTG11 and CTG13 inhibit NLRP3 inflammasome activation in mouse BMDMs.The structure of CTG11.Western blot analysis of IL-1β, caspase-1in culture supernatantsand pro-IL-1β, caspase-1, NLRP3, ASC in whole cell lysatesof LPS-primed BMDMs treated with CTG11 and then stimulated with Nigericin, supernatants were collected for the measurement of caspase-1, IL-1β, LDHand TNF-α.The structure of CTG13.Western blot analysis of IL-1β, caspase-1in culture supernatantsand pro-IL-1β, caspase-1, NLRP3, ASC in whole cell lysatesof LPS-primed BMDMs treated with CTG13 and then stimulated with Nigericin, supernatants were collected for the measurement of caspase-1, IL-1β, LDHand TNF-α. Coomassie blue–stained gels used as loading control and Lamin B used as a control for equal loading of the samples. Data represent as mean ± SEM. Compared to con, ** p < 0.01, ***p < 0.001, **** p < 0.0001; compared to a concentration of 0 μM, ###p < 0.001, ####p < 0.0001 and ns: not significant. Supplementary Figure 3. CTG12 impedes the priming process of NLRP3 inflammasome activation and specifically inhibits canonical and noncanonical NLRP3 inflammasome activation.BMDMs were primed with LPS treated with CTG12, then stimulated with Nigericin ATP, poly, or SiO₂. Supernatants were collected for the measurement of TNF-α, BMDMs primed with Pam3CSK4 treated with CTG12, followed by cytosolic LPS. Supernatants were collected fo [file 12964_2026_2741_MOESM1_ESM.zip › supplementary file/Figure3-Supplementary Figure3原膜/Figure3-G-NLRP3-Lamin B.png]

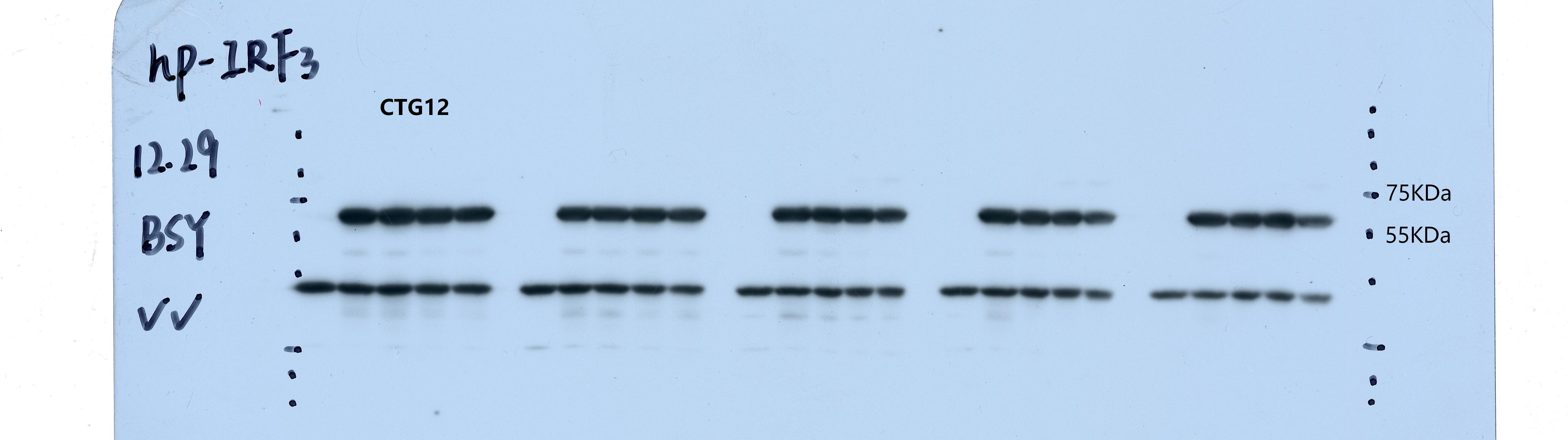

Supplement: Supplementary file 1 — Supplementary Material 1. Supplementary Figure 1. Licochalcone B derivatives inhibit NLRP3 inflammasome activation.BMDMs were primed with LPS for 4 hours, treated with Echinatin, Licorice Chalcone B, CTG4, CTG1, CTG10, CTG11, CTG12, CTG13, CTG14, CTG15, CTG16, CTG18, CTG19, CAPE, CTG23for 30 minutes, and then stimulated with nigericin for 25 minutes. Supernatants were collected for the measurement of caspase-1. Data represent as mean ± SEM. Compared to con, **** p < 0.0001; compared to a concentration of 0 μM, ###p < 0.001, #### p < 0.0001 and ns:not significant. Supplementary Figure 2. CTG11 and CTG13 inhibit NLRP3 inflammasome activation in mouse BMDMs.The structure of CTG11.Western blot analysis of IL-1β, caspase-1in culture supernatantsand pro-IL-1β, caspase-1, NLRP3, ASC in whole cell lysatesof LPS-primed BMDMs treated with CTG11 and then stimulated with Nigericin, supernatants were collected for the measurement of caspase-1, IL-1β, LDHand TNF-α.The structure of CTG13.Western blot analysis of IL-1β, caspase-1in culture supernatantsand pro-IL-1β, caspase-1, NLRP3, ASC in whole cell lysatesof LPS-primed BMDMs treated with CTG13 and then stimulated with Nigericin, supernatants were collected for the measurement of caspase-1, IL-1β, LDHand TNF-α. Coomassie blue–stained gels used as loading control and Lamin B used as a control for equal loading of the samples. Data represent as mean ± SEM. Compared to con, ** p < 0.01, ***p < 0.001, **** p < 0.0001; compared to a concentration of 0 μM, ###p < 0.001, ####p < 0.0001 and ns: not significant. Supplementary Figure 3. CTG12 impedes the priming process of NLRP3 inflammasome activation and specifically inhibits canonical and noncanonical NLRP3 inflammasome activation.BMDMs were primed with LPS treated with CTG12, then stimulated with Nigericin ATP, poly, or SiO₂. Supernatants were collected for the measurement of TNF-α, BMDMs primed with Pam3CSK4 treated with CTG12, followed by cytosolic LPS. Supernatants were collected fo [file 12964_2026_2741_MOESM1_ESM.zip › supplementary file/Figure3-Supplementary Figure3原膜/Supplementary Figure3-D-hP-IRF3.jpg]

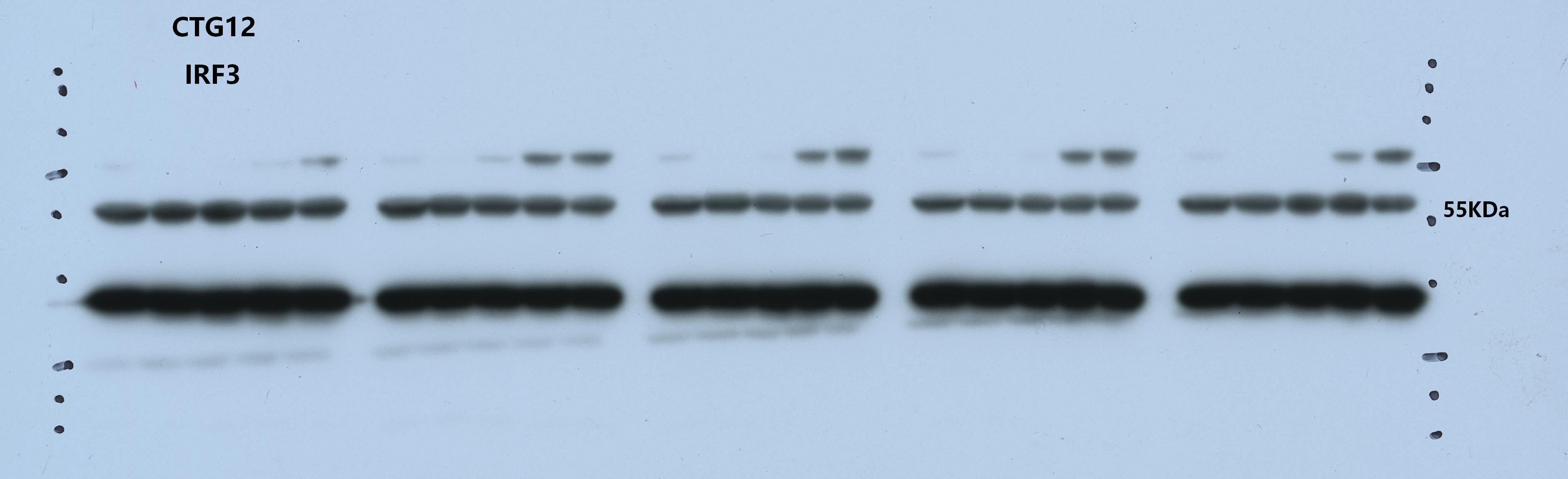

Supplement: Supplementary file 1 — Supplementary Material 1. Supplementary Figure 1. Licochalcone B derivatives inhibit NLRP3 inflammasome activation.BMDMs were primed with LPS for 4 hours, treated with Echinatin, Licorice Chalcone B, CTG4, CTG1, CTG10, CTG11, CTG12, CTG13, CTG14, CTG15, CTG16, CTG18, CTG19, CAPE, CTG23for 30 minutes, and then stimulated with nigericin for 25 minutes. Supernatants were collected for the measurement of caspase-1. Data represent as mean ± SEM. Compared to con, **** p < 0.0001; compared to a concentration of 0 μM, ###p < 0.001, #### p < 0.0001 and ns:not significant. Supplementary Figure 2. CTG11 and CTG13 inhibit NLRP3 inflammasome activation in mouse BMDMs.The structure of CTG11.Western blot analysis of IL-1β, caspase-1in culture supernatantsand pro-IL-1β, caspase-1, NLRP3, ASC in whole cell lysatesof LPS-primed BMDMs treated with CTG11 and then stimulated with Nigericin, supernatants were collected for the measurement of caspase-1, IL-1β, LDHand TNF-α.The structure of CTG13.Western blot analysis of IL-1β, caspase-1in culture supernatantsand pro-IL-1β, caspase-1, NLRP3, ASC in whole cell lysatesof LPS-primed BMDMs treated with CTG13 and then stimulated with Nigericin, supernatants were collected for the measurement of caspase-1, IL-1β, LDHand TNF-α. Coomassie blue–stained gels used as loading control and Lamin B used as a control for equal loading of the samples. Data represent as mean ± SEM. Compared to con, ** p < 0.01, ***p < 0.001, **** p < 0.0001; compared to a concentration of 0 μM, ###p < 0.001, ####p < 0.0001 and ns: not significant. Supplementary Figure 3. CTG12 impedes the priming process of NLRP3 inflammasome activation and specifically inhibits canonical and noncanonical NLRP3 inflammasome activation.BMDMs were primed with LPS treated with CTG12, then stimulated with Nigericin ATP, poly, or SiO₂. Supernatants were collected for the measurement of TNF-α, BMDMs primed with Pam3CSK4 treated with CTG12, followed by cytosolic LPS. Supernatants were collected fo [file 12964_2026_2741_MOESM1_ESM.zip › supplementary file/Figure3-Supplementary Figure3原膜/Supplementary Figure3-D-IRF3.jpg]

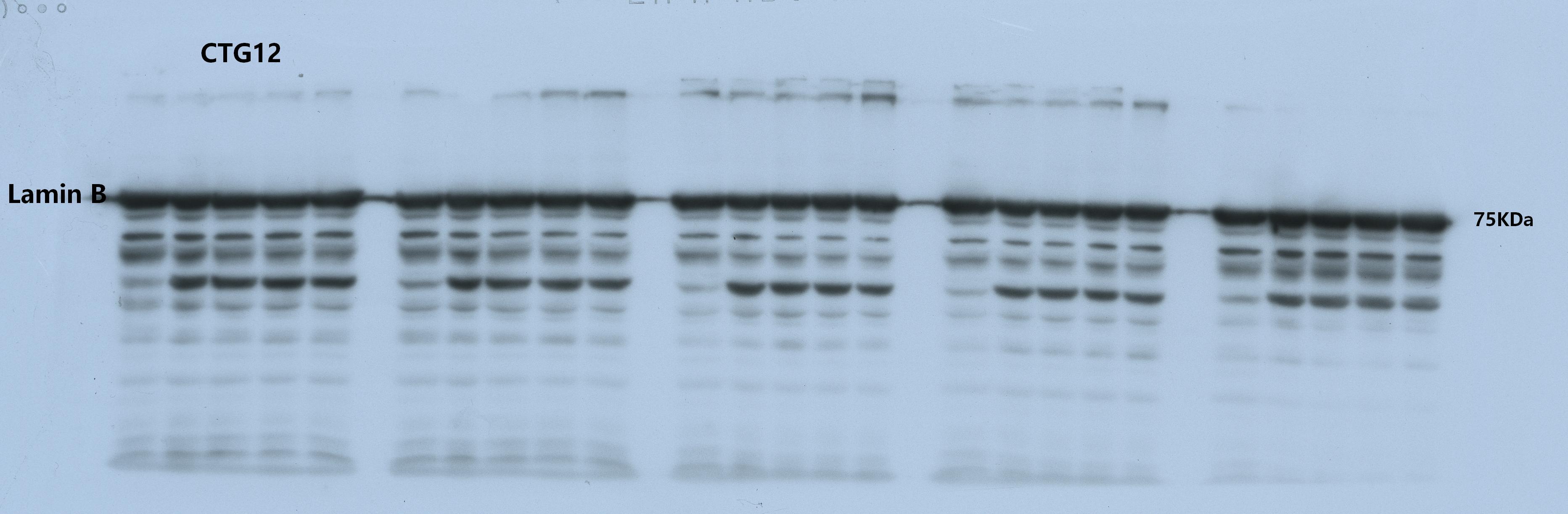

Supplement: Supplementary file 1 — Supplementary Material 1. Supplementary Figure 1. Licochalcone B derivatives inhibit NLRP3 inflammasome activation.BMDMs were primed with LPS for 4 hours, treated with Echinatin, Licorice Chalcone B, CTG4, CTG1, CTG10, CTG11, CTG12, CTG13, CTG14, CTG15, CTG16, CTG18, CTG19, CAPE, CTG23for 30 minutes, and then stimulated with nigericin for 25 minutes. Supernatants were collected for the measurement of caspase-1. Data represent as mean ± SEM. Compared to con, **** p < 0.0001; compared to a concentration of 0 μM, ###p < 0.001, #### p < 0.0001 and ns:not significant. Supplementary Figure 2. CTG11 and CTG13 inhibit NLRP3 inflammasome activation in mouse BMDMs.The structure of CTG11.Western blot analysis of IL-1β, caspase-1in culture supernatantsand pro-IL-1β, caspase-1, NLRP3, ASC in whole cell lysatesof LPS-primed BMDMs treated with CTG11 and then stimulated with Nigericin, supernatants were collected for the measurement of caspase-1, IL-1β, LDHand TNF-α.The structure of CTG13.Western blot analysis of IL-1β, caspase-1in culture supernatantsand pro-IL-1β, caspase-1, NLRP3, ASC in whole cell lysatesof LPS-primed BMDMs treated with CTG13 and then stimulated with Nigericin, supernatants were collected for the measurement of caspase-1, IL-1β, LDHand TNF-α. Coomassie blue–stained gels used as loading control and Lamin B used as a control for equal loading of the samples. Data represent as mean ± SEM. Compared to con, ** p < 0.01, ***p < 0.001, **** p < 0.0001; compared to a concentration of 0 μM, ###p < 0.001, ####p < 0.0001 and ns: not significant. Supplementary Figure 3. CTG12 impedes the priming process of NLRP3 inflammasome activation and specifically inhibits canonical and noncanonical NLRP3 inflammasome activation.BMDMs were primed with LPS treated with CTG12, then stimulated with Nigericin ATP, poly, or SiO₂. Supernatants were collected for the measurement of TNF-α, BMDMs primed with Pam3CSK4 treated with CTG12, followed by cytosolic LPS. Supernatants were collected fo [file 12964_2026_2741_MOESM1_ESM.zip › supplementary file/Figure3-Supplementary Figure3原膜/Supplementary Figure3-D-Lamin B.jpg]

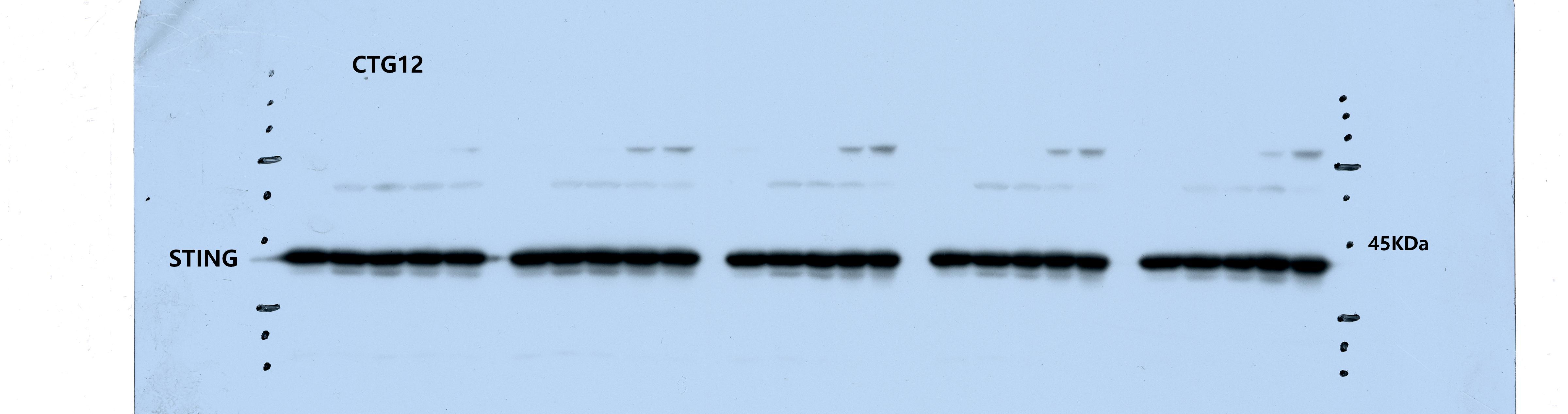

Supplement: Supplementary file 1 — Supplementary Material 1. Supplementary Figure 1. Licochalcone B derivatives inhibit NLRP3 inflammasome activation.BMDMs were primed with LPS for 4 hours, treated with Echinatin, Licorice Chalcone B, CTG4, CTG1, CTG10, CTG11, CTG12, CTG13, CTG14, CTG15, CTG16, CTG18, CTG19, CAPE, CTG23for 30 minutes, and then stimulated with nigericin for 25 minutes. Supernatants were collected for the measurement of caspase-1. Data represent as mean ± SEM. Compared to con, **** p < 0.0001; compared to a concentration of 0 μM, ###p < 0.001, #### p < 0.0001 and ns:not significant. Supplementary Figure 2. CTG11 and CTG13 inhibit NLRP3 inflammasome activation in mouse BMDMs.The structure of CTG11.Western blot analysis of IL-1β, caspase-1in culture supernatantsand pro-IL-1β, caspase-1, NLRP3, ASC in whole cell lysatesof LPS-primed BMDMs treated with CTG11 and then stimulated with Nigericin, supernatants were collected for the measurement of caspase-1, IL-1β, LDHand TNF-α.The structure of CTG13.Western blot analysis of IL-1β, caspase-1in culture supernatantsand pro-IL-1β, caspase-1, NLRP3, ASC in whole cell lysatesof LPS-primed BMDMs treated with CTG13 and then stimulated with Nigericin, supernatants were collected for the measurement of caspase-1, IL-1β, LDHand TNF-α. Coomassie blue–stained gels used as loading control and Lamin B used as a control for equal loading of the samples. Data represent as mean ± SEM. Compared to con, ** p < 0.01, ***p < 0.001, **** p < 0.0001; compared to a concentration of 0 μM, ###p < 0.001, ####p < 0.0001 and ns: not significant. Supplementary Figure 3. CTG12 impedes the priming process of NLRP3 inflammasome activation and specifically inhibits canonical and noncanonical NLRP3 inflammasome activation.BMDMs were primed with LPS treated with CTG12, then stimulated with Nigericin ATP, poly, or SiO₂. Supernatants were collected for the measurement of TNF-α, BMDMs primed with Pam3CSK4 treated with CTG12, followed by cytosolic LPS. Supernatants were collected fo [file 12964_2026_2741_MOESM1_ESM.zip › supplementary file/Figure3-Supplementary Figure3原膜/Supplementary Figure3-D-STING.jpg]

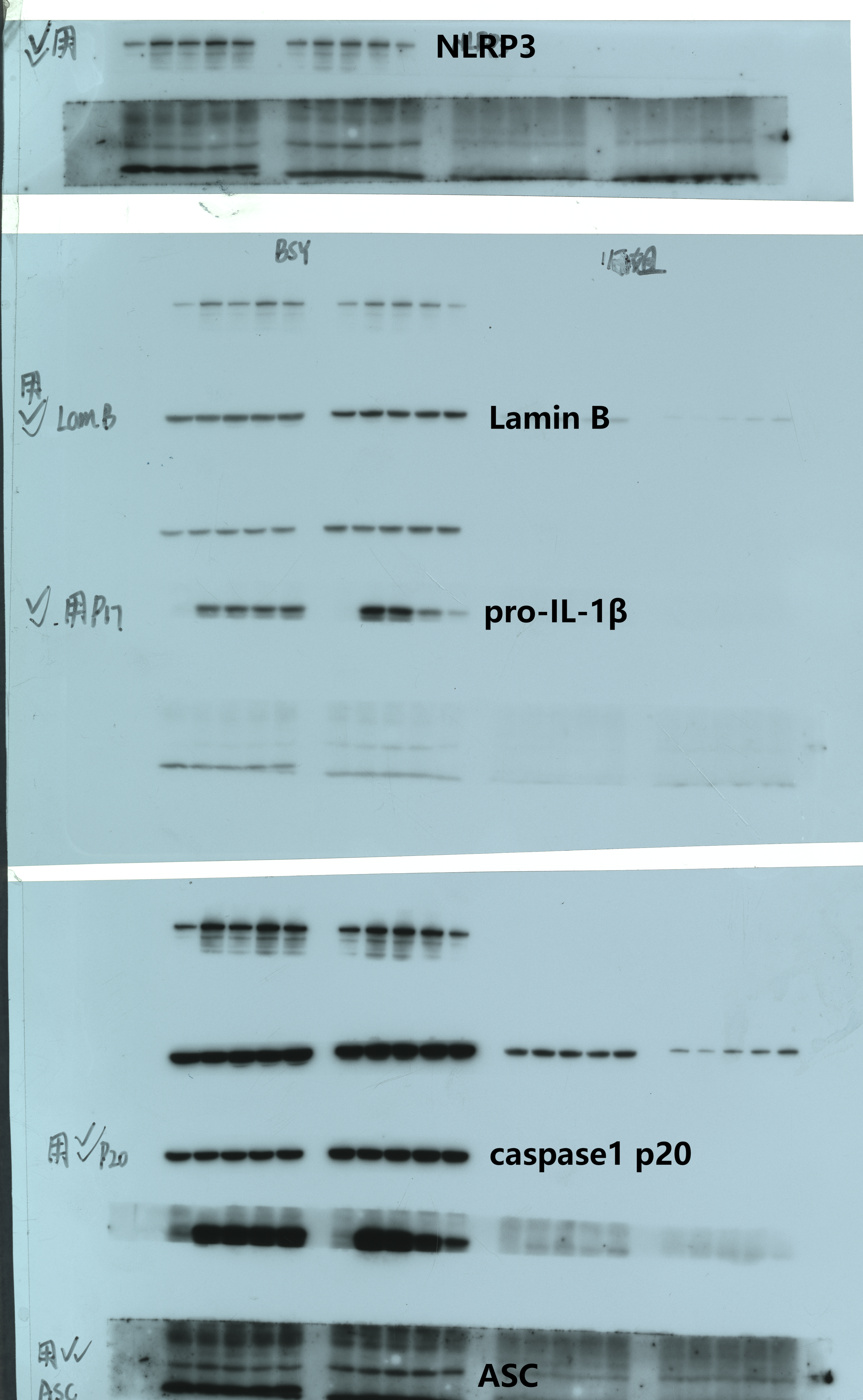

Supplement: Supplementary file 1 — Supplementary Material 1. Supplementary Figure 1. Licochalcone B derivatives inhibit NLRP3 inflammasome activation.BMDMs were primed with LPS for 4 hours, treated with Echinatin, Licorice Chalcone B, CTG4, CTG1, CTG10, CTG11, CTG12, CTG13, CTG14, CTG15, CTG16, CTG18, CTG19, CAPE, CTG23for 30 minutes, and then stimulated with nigericin for 25 minutes. Supernatants were collected for the measurement of caspase-1. Data represent as mean ± SEM. Compared to con, **** p < 0.0001; compared to a concentration of 0 μM, ###p < 0.001, #### p < 0.0001 and ns:not significant. Supplementary Figure 2. CTG11 and CTG13 inhibit NLRP3 inflammasome activation in mouse BMDMs.The structure of CTG11.Western blot analysis of IL-1β, caspase-1in culture supernatantsand pro-IL-1β, caspase-1, NLRP3, ASC in whole cell lysatesof LPS-primed BMDMs treated with CTG11 and then stimulated with Nigericin, supernatants were collected for the measurement of caspase-1, IL-1β, LDHand TNF-α.The structure of CTG13.Western blot analysis of IL-1β, caspase-1in culture supernatantsand pro-IL-1β, caspase-1, NLRP3, ASC in whole cell lysatesof LPS-primed BMDMs treated with CTG13 and then stimulated with Nigericin, supernatants were collected for the measurement of caspase-1, IL-1β, LDHand TNF-α. Coomassie blue–stained gels used as loading control and Lamin B used as a control for equal loading of the samples. Data represent as mean ± SEM. Compared to con, ** p < 0.01, ***p < 0.001, **** p < 0.0001; compared to a concentration of 0 μM, ###p < 0.001, ####p < 0.0001 and ns: not significant. Supplementary Figure 3. CTG12 impedes the priming process of NLRP3 inflammasome activation and specifically inhibits canonical and noncanonical NLRP3 inflammasome activation.BMDMs were primed with LPS treated with CTG12, then stimulated with Nigericin ATP, poly, or SiO₂. Supernatants were collected for the measurement of TNF-α, BMDMs primed with Pam3CSK4 treated with CTG12, followed by cytosolic LPS. Supernatants were collected fo [file 12964_2026_2741_MOESM1_ESM.zip › supplementary file/Figure4原膜/Figure4-A-NF-KB-NLRP3-pro-IL-1β-caspase-1 P45-ASC-Lamin B -.png]

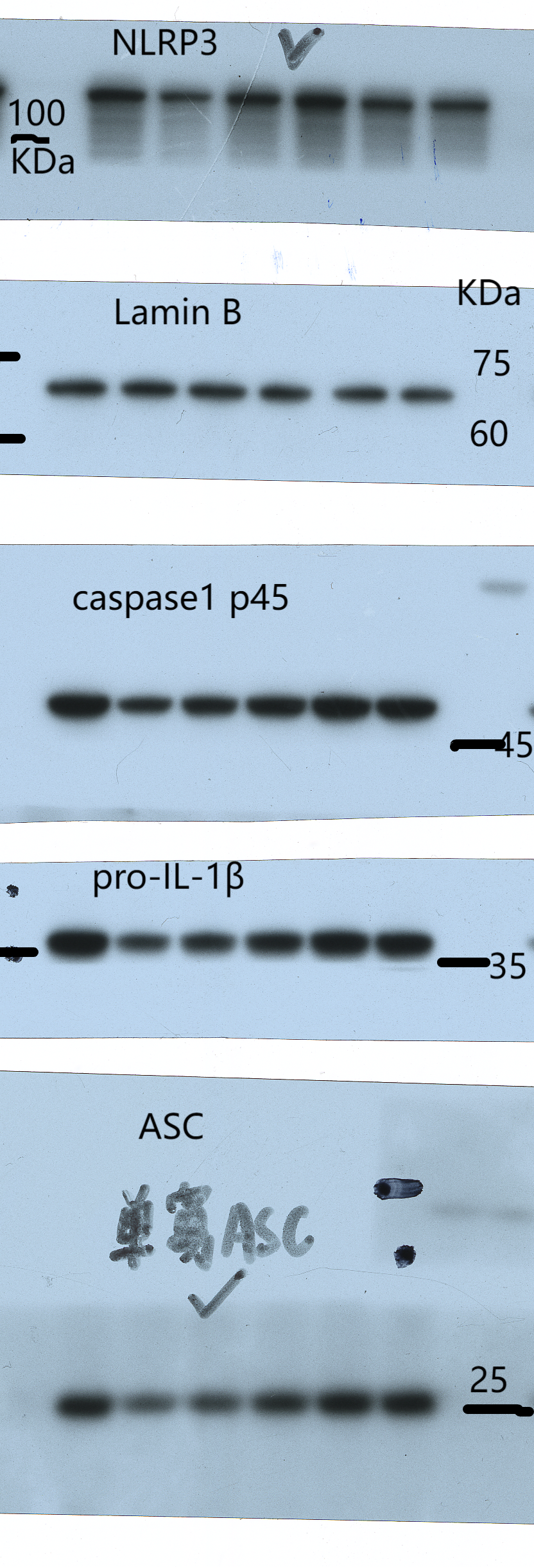

Supplement: Supplementary file 1 — Supplementary Material 1. Supplementary Figure 1. Licochalcone B derivatives inhibit NLRP3 inflammasome activation.BMDMs were primed with LPS for 4 hours, treated with Echinatin, Licorice Chalcone B, CTG4, CTG1, CTG10, CTG11, CTG12, CTG13, CTG14, CTG15, CTG16, CTG18, CTG19, CAPE, CTG23for 30 minutes, and then stimulated with nigericin for 25 minutes. Supernatants were collected for the measurement of caspase-1. Data represent as mean ± SEM. Compared to con, **** p < 0.0001; compared to a concentration of 0 μM, ###p < 0.001, #### p < 0.0001 and ns:not significant. Supplementary Figure 2. CTG11 and CTG13 inhibit NLRP3 inflammasome activation in mouse BMDMs.The structure of CTG11.Western blot analysis of IL-1β, caspase-1in culture supernatantsand pro-IL-1β, caspase-1, NLRP3, ASC in whole cell lysatesof LPS-primed BMDMs treated with CTG11 and then stimulated with Nigericin, supernatants were collected for the measurement of caspase-1, IL-1β, LDHand TNF-α.The structure of CTG13.Western blot analysis of IL-1β, caspase-1in culture supernatantsand pro-IL-1β, caspase-1, NLRP3, ASC in whole cell lysatesof LPS-primed BMDMs treated with CTG13 and then stimulated with Nigericin, supernatants were collected for the measurement of caspase-1, IL-1β, LDHand TNF-α. Coomassie blue–stained gels used as loading control and Lamin B used as a control for equal loading of the samples. Data represent as mean ± SEM. Compared to con, ** p < 0.01, ***p < 0.001, **** p < 0.0001; compared to a concentration of 0 μM, ###p < 0.001, ####p < 0.0001 and ns: not significant. Supplementary Figure 3. CTG12 impedes the priming process of NLRP3 inflammasome activation and specifically inhibits canonical and noncanonical NLRP3 inflammasome activation.BMDMs were primed with LPS treated with CTG12, then stimulated with Nigericin ATP, poly, or SiO₂. Supernatants were collected for the measurement of TNF-α, BMDMs primed with Pam3CSK4 treated with CTG12, followed by cytosolic LPS. Supernatants were collected fo [file 12964_2026_2741_MOESM1_ESM.zip › supplementary file/Figure4原膜/Figure4-B--NLRP3-ASC-caspase1 P45-pro-IL-1β-Lamin B.png]

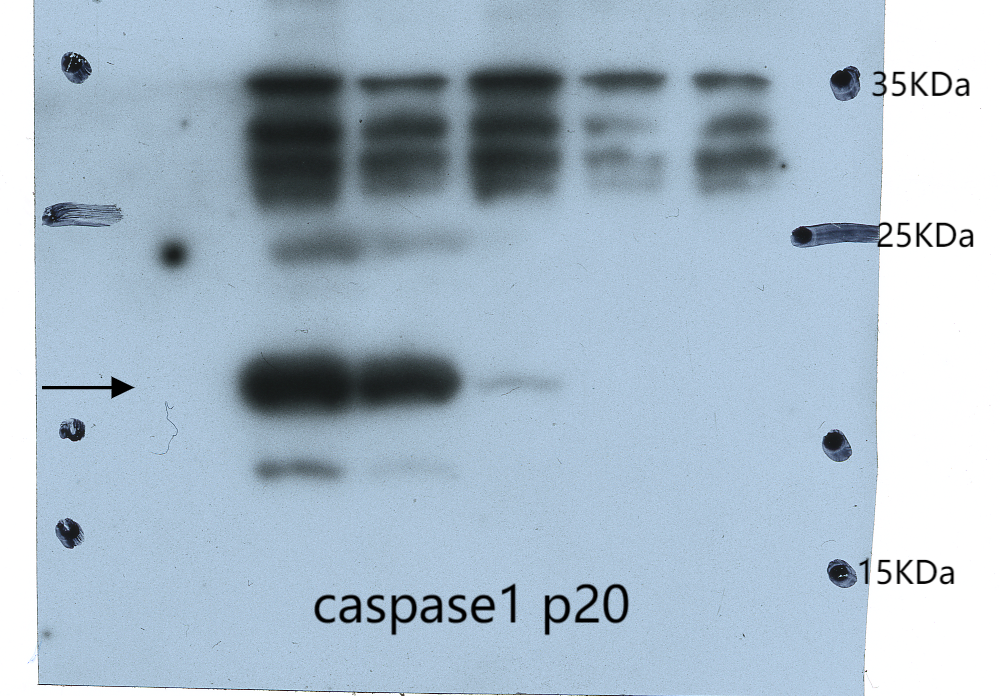

Supplement: Supplementary file 1 — Supplementary Material 1. Supplementary Figure 1. Licochalcone B derivatives inhibit NLRP3 inflammasome activation.BMDMs were primed with LPS for 4 hours, treated with Echinatin, Licorice Chalcone B, CTG4, CTG1, CTG10, CTG11, CTG12, CTG13, CTG14, CTG15, CTG16, CTG18, CTG19, CAPE, CTG23for 30 minutes, and then stimulated with nigericin for 25 minutes. Supernatants were collected for the measurement of caspase-1. Data represent as mean ± SEM. Compared to con, **** p < 0.0001; compared to a concentration of 0 μM, ###p < 0.001, #### p < 0.0001 and ns:not significant. Supplementary Figure 2. CTG11 and CTG13 inhibit NLRP3 inflammasome activation in mouse BMDMs.The structure of CTG11.Western blot analysis of IL-1β, caspase-1in culture supernatantsand pro-IL-1β, caspase-1, NLRP3, ASC in whole cell lysatesof LPS-primed BMDMs treated with CTG11 and then stimulated with Nigericin, supernatants were collected for the measurement of caspase-1, IL-1β, LDHand TNF-α.The structure of CTG13.Western blot analysis of IL-1β, caspase-1in culture supernatantsand pro-IL-1β, caspase-1, NLRP3, ASC in whole cell lysatesof LPS-primed BMDMs treated with CTG13 and then stimulated with Nigericin, supernatants were collected for the measurement of caspase-1, IL-1β, LDHand TNF-α. Coomassie blue–stained gels used as loading control and Lamin B used as a control for equal loading of the samples. Data represent as mean ± SEM. Compared to con, ** p < 0.01, ***p < 0.001, **** p < 0.0001; compared to a concentration of 0 μM, ###p < 0.001, ####p < 0.0001 and ns: not significant. Supplementary Figure 3. CTG12 impedes the priming process of NLRP3 inflammasome activation and specifically inhibits canonical and noncanonical NLRP3 inflammasome activation.BMDMs were primed with LPS treated with CTG12, then stimulated with Nigericin ATP, poly, or SiO₂. Supernatants were collected for the measurement of TNF-α, BMDMs primed with Pam3CSK4 treated with CTG12, followed by cytosolic LPS. Supernatants were collected fo [file 12964_2026_2741_MOESM1_ESM.zip › supplementary file/Figure4原膜/Figure4-B-caspase1p20.png]

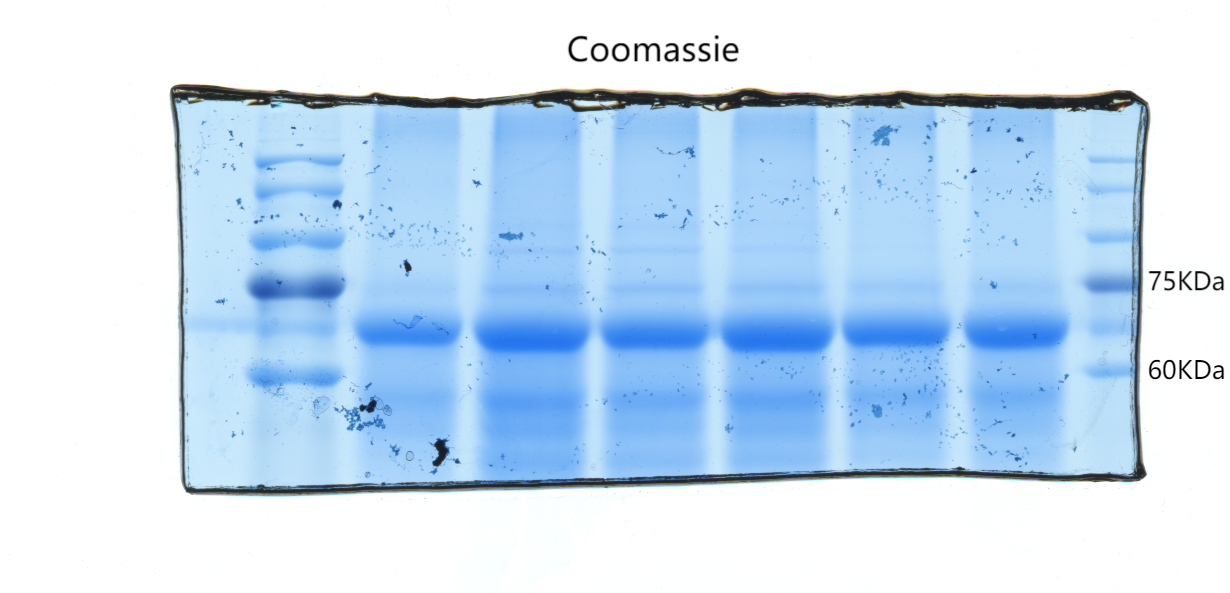

Supplement: Supplementary file 1 — Supplementary Material 1. Supplementary Figure 1. Licochalcone B derivatives inhibit NLRP3 inflammasome activation.BMDMs were primed with LPS for 4 hours, treated with Echinatin, Licorice Chalcone B, CTG4, CTG1, CTG10, CTG11, CTG12, CTG13, CTG14, CTG15, CTG16, CTG18, CTG19, CAPE, CTG23for 30 minutes, and then stimulated with nigericin for 25 minutes. Supernatants were collected for the measurement of caspase-1. Data represent as mean ± SEM. Compared to con, **** p < 0.0001; compared to a concentration of 0 μM, ###p < 0.001, #### p < 0.0001 and ns:not significant. Supplementary Figure 2. CTG11 and CTG13 inhibit NLRP3 inflammasome activation in mouse BMDMs.The structure of CTG11.Western blot analysis of IL-1β, caspase-1in culture supernatantsand pro-IL-1β, caspase-1, NLRP3, ASC in whole cell lysatesof LPS-primed BMDMs treated with CTG11 and then stimulated with Nigericin, supernatants were collected for the measurement of caspase-1, IL-1β, LDHand TNF-α.The structure of CTG13.Western blot analysis of IL-1β, caspase-1in culture supernatantsand pro-IL-1β, caspase-1, NLRP3, ASC in whole cell lysatesof LPS-primed BMDMs treated with CTG13 and then stimulated with Nigericin, supernatants were collected for the measurement of caspase-1, IL-1β, LDHand TNF-α. Coomassie blue–stained gels used as loading control and Lamin B used as a control for equal loading of the samples. Data represent as mean ± SEM. Compared to con, ** p < 0.01, ***p < 0.001, **** p < 0.0001; compared to a concentration of 0 μM, ###p < 0.001, ####p < 0.0001 and ns: not significant. Supplementary Figure 3. CTG12 impedes the priming process of NLRP3 inflammasome activation and specifically inhibits canonical and noncanonical NLRP3 inflammasome activation.BMDMs were primed with LPS treated with CTG12, then stimulated with Nigericin ATP, poly, or SiO₂. Supernatants were collected for the measurement of TNF-α, BMDMs primed with Pam3CSK4 treated with CTG12, followed by cytosolic LPS. Supernatants were collected fo [file 12964_2026_2741_MOESM1_ESM.zip › supplementary file/Figure4原膜/Figure4-B-Coomassie.png]

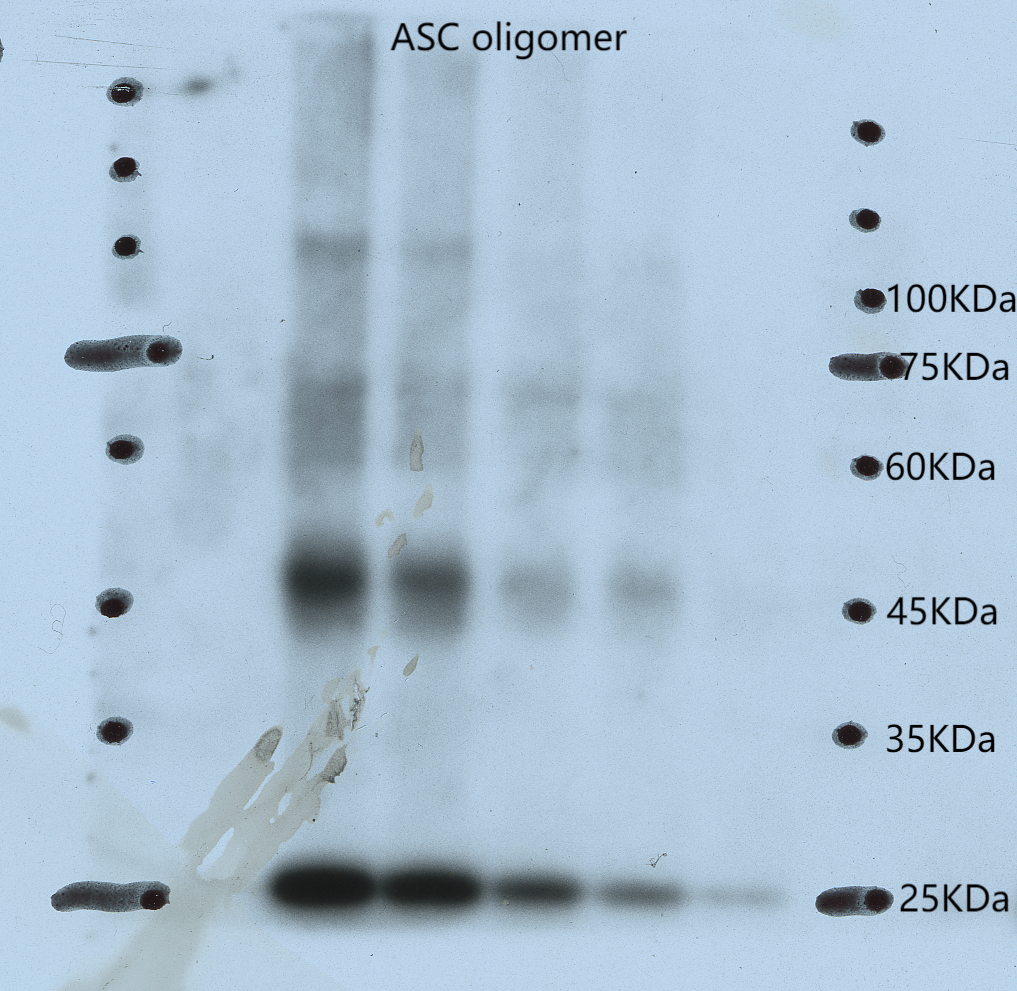

Supplement: Supplementary file 1 — Supplementary Material 1. Supplementary Figure 1. Licochalcone B derivatives inhibit NLRP3 inflammasome activation.BMDMs were primed with LPS for 4 hours, treated with Echinatin, Licorice Chalcone B, CTG4, CTG1, CTG10, CTG11, CTG12, CTG13, CTG14, CTG15, CTG16, CTG18, CTG19, CAPE, CTG23for 30 minutes, and then stimulated with nigericin for 25 minutes. Supernatants were collected for the measurement of caspase-1. Data represent as mean ± SEM. Compared to con, **** p < 0.0001; compared to a concentration of 0 μM, ###p < 0.001, #### p < 0.0001 and ns:not significant. Supplementary Figure 2. CTG11 and CTG13 inhibit NLRP3 inflammasome activation in mouse BMDMs.The structure of CTG11.Western blot analysis of IL-1β, caspase-1in culture supernatantsand pro-IL-1β, caspase-1, NLRP3, ASC in whole cell lysatesof LPS-primed BMDMs treated with CTG11 and then stimulated with Nigericin, supernatants were collected for the measurement of caspase-1, IL-1β, LDHand TNF-α.The structure of CTG13.Western blot analysis of IL-1β, caspase-1in culture supernatantsand pro-IL-1β, caspase-1, NLRP3, ASC in whole cell lysatesof LPS-primed BMDMs treated with CTG13 and then stimulated with Nigericin, supernatants were collected for the measurement of caspase-1, IL-1β, LDHand TNF-α. Coomassie blue–stained gels used as loading control and Lamin B used as a control for equal loading of the samples. Data represent as mean ± SEM. Compared to con, ** p < 0.01, ***p < 0.001, **** p < 0.0001; compared to a concentration of 0 μM, ###p < 0.001, ####p < 0.0001 and ns: not significant. Supplementary Figure 3. CTG12 impedes the priming process of NLRP3 inflammasome activation and specifically inhibits canonical and noncanonical NLRP3 inflammasome activation.BMDMs were primed with LPS treated with CTG12, then stimulated with Nigericin ATP, poly, or SiO₂. Supernatants were collected for the measurement of TNF-α, BMDMs primed with Pam3CSK4 treated with CTG12, followed by cytosolic LPS. Supernatants were collected fo [file 12964_2026_2741_MOESM1_ESM.zip › supplementary file/Figure4原膜/Figure4-B-Triton X-insoluble.png]

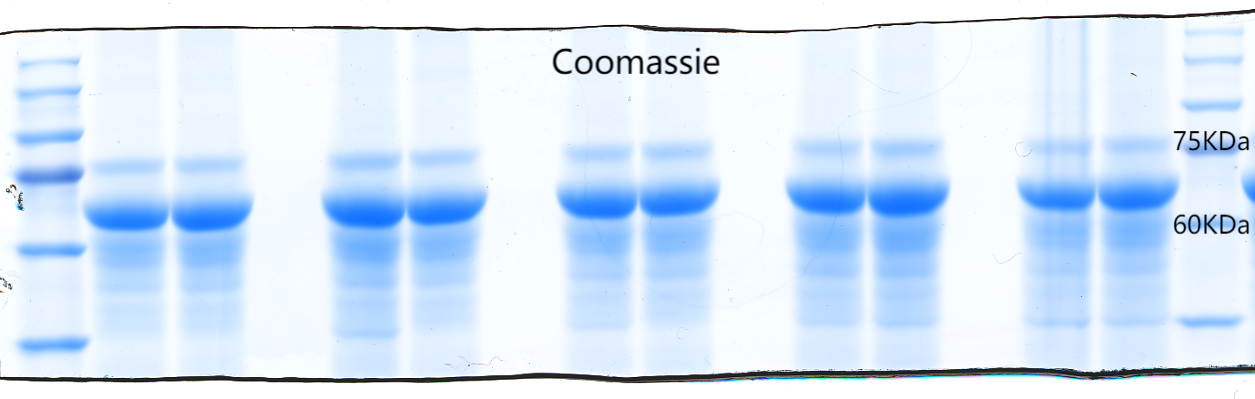

Supplement: Supplementary file 1 — Supplementary Material 1. Supplementary Figure 1. Licochalcone B derivatives inhibit NLRP3 inflammasome activation.BMDMs were primed with LPS for 4 hours, treated with Echinatin, Licorice Chalcone B, CTG4, CTG1, CTG10, CTG11, CTG12, CTG13, CTG14, CTG15, CTG16, CTG18, CTG19, CAPE, CTG23for 30 minutes, and then stimulated with nigericin for 25 minutes. Supernatants were collected for the measurement of caspase-1. Data represent as mean ± SEM. Compared to con, **** p < 0.0001; compared to a concentration of 0 μM, ###p < 0.001, #### p < 0.0001 and ns:not significant. Supplementary Figure 2. CTG11 and CTG13 inhibit NLRP3 inflammasome activation in mouse BMDMs.The structure of CTG11.Western blot analysis of IL-1β, caspase-1in culture supernatantsand pro-IL-1β, caspase-1, NLRP3, ASC in whole cell lysatesof LPS-primed BMDMs treated with CTG11 and then stimulated with Nigericin, supernatants were collected for the measurement of caspase-1, IL-1β, LDHand TNF-α.The structure of CTG13.Western blot analysis of IL-1β, caspase-1in culture supernatantsand pro-IL-1β, caspase-1, NLRP3, ASC in whole cell lysatesof LPS-primed BMDMs treated with CTG13 and then stimulated with Nigericin, supernatants were collected for the measurement of caspase-1, IL-1β, LDHand TNF-α. Coomassie blue–stained gels used as loading control and Lamin B used as a control for equal loading of the samples. Data represent as mean ± SEM. Compared to con, ** p < 0.01, ***p < 0.001, **** p < 0.0001; compared to a concentration of 0 μM, ###p < 0.001, ####p < 0.0001 and ns: not significant. Supplementary Figure 3. CTG12 impedes the priming process of NLRP3 inflammasome activation and specifically inhibits canonical and noncanonical NLRP3 inflammasome activation.BMDMs were primed with LPS treated with CTG12, then stimulated with Nigericin ATP, poly, or SiO₂. Supernatants were collected for the measurement of TNF-α, BMDMs primed with Pam3CSK4 treated with CTG12, followed by cytosolic LPS. Supernatants were collected fo [file 12964_2026_2741_MOESM1_ESM.zip › supplementary file/Figure4原膜/Figure4-C-Coomassie.png]

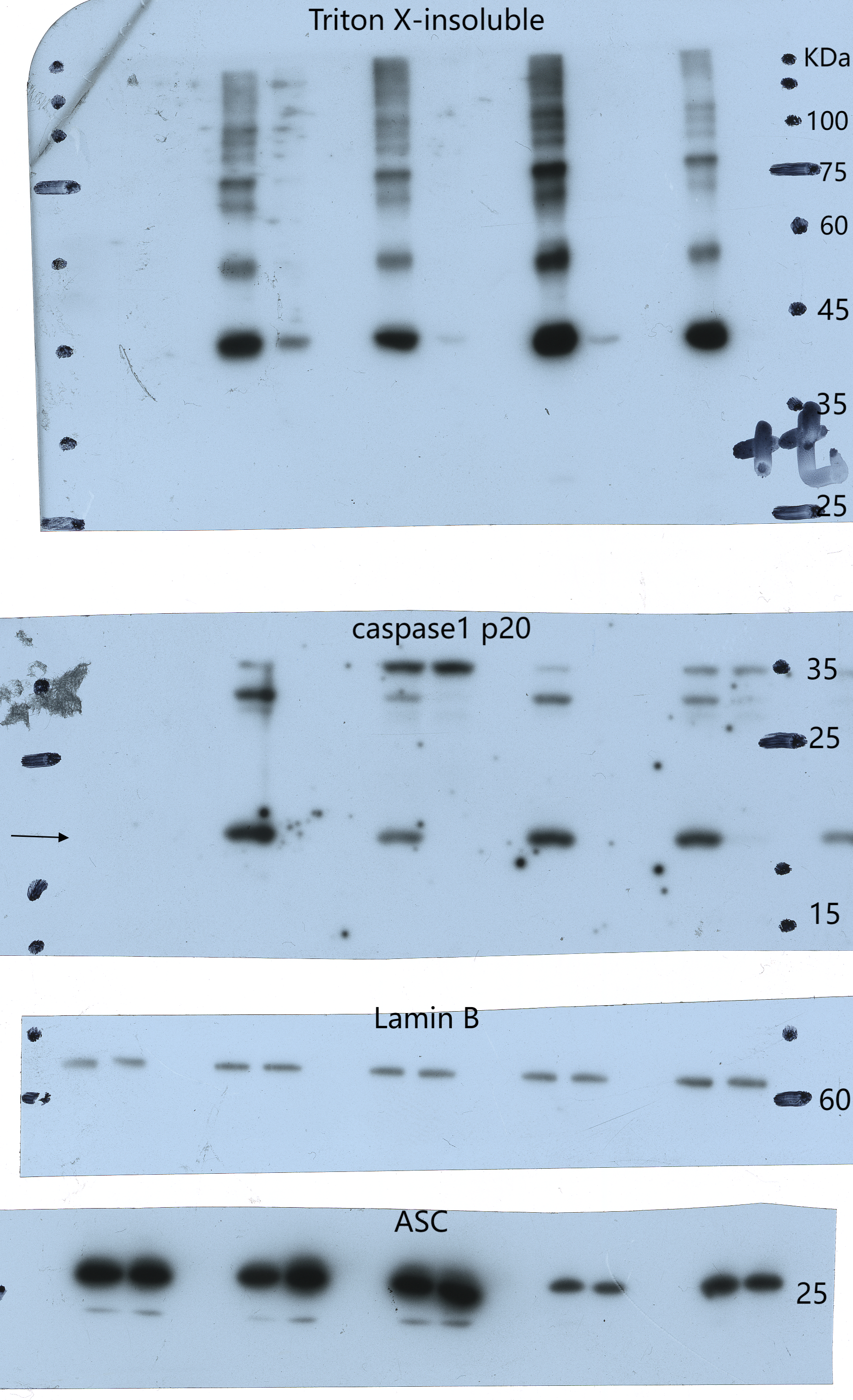

Supplement: Supplementary file 1 — Supplementary Material 1. Supplementary Figure 1. Licochalcone B derivatives inhibit NLRP3 inflammasome activation.BMDMs were primed with LPS for 4 hours, treated with Echinatin, Licorice Chalcone B, CTG4, CTG1, CTG10, CTG11, CTG12, CTG13, CTG14, CTG15, CTG16, CTG18, CTG19, CAPE, CTG23for 30 minutes, and then stimulated with nigericin for 25 minutes. Supernatants were collected for the measurement of caspase-1. Data represent as mean ± SEM. Compared to con, **** p < 0.0001; compared to a concentration of 0 μM, ###p < 0.001, #### p < 0.0001 and ns:not significant. Supplementary Figure 2. CTG11 and CTG13 inhibit NLRP3 inflammasome activation in mouse BMDMs.The structure of CTG11.Western blot analysis of IL-1β, caspase-1in culture supernatantsand pro-IL-1β, caspase-1, NLRP3, ASC in whole cell lysatesof LPS-primed BMDMs treated with CTG11 and then stimulated with Nigericin, supernatants were collected for the measurement of caspase-1, IL-1β, LDHand TNF-α.The structure of CTG13.Western blot analysis of IL-1β, caspase-1in culture supernatantsand pro-IL-1β, caspase-1, NLRP3, ASC in whole cell lysatesof LPS-primed BMDMs treated with CTG13 and then stimulated with Nigericin, supernatants were collected for the measurement of caspase-1, IL-1β, LDHand TNF-α. Coomassie blue–stained gels used as loading control and Lamin B used as a control for equal loading of the samples. Data represent as mean ± SEM. Compared to con, ** p < 0.01, ***p < 0.001, **** p < 0.0001; compared to a concentration of 0 μM, ###p < 0.001, ####p < 0.0001 and ns: not significant. Supplementary Figure 3. CTG12 impedes the priming process of NLRP3 inflammasome activation and specifically inhibits canonical and noncanonical NLRP3 inflammasome activation.BMDMs were primed with LPS treated with CTG12, then stimulated with Nigericin ATP, poly, or SiO₂. Supernatants were collected for the measurement of TNF-α, BMDMs primed with Pam3CSK4 treated with CTG12, followed by cytosolic LPS. Supernatants were collected fo [file 12964_2026_2741_MOESM1_ESM.zip › supplementary file/Figure4原膜/Figure4-C-Triton X-insoluble-caspase1p20-ASC-Lamin B.png]

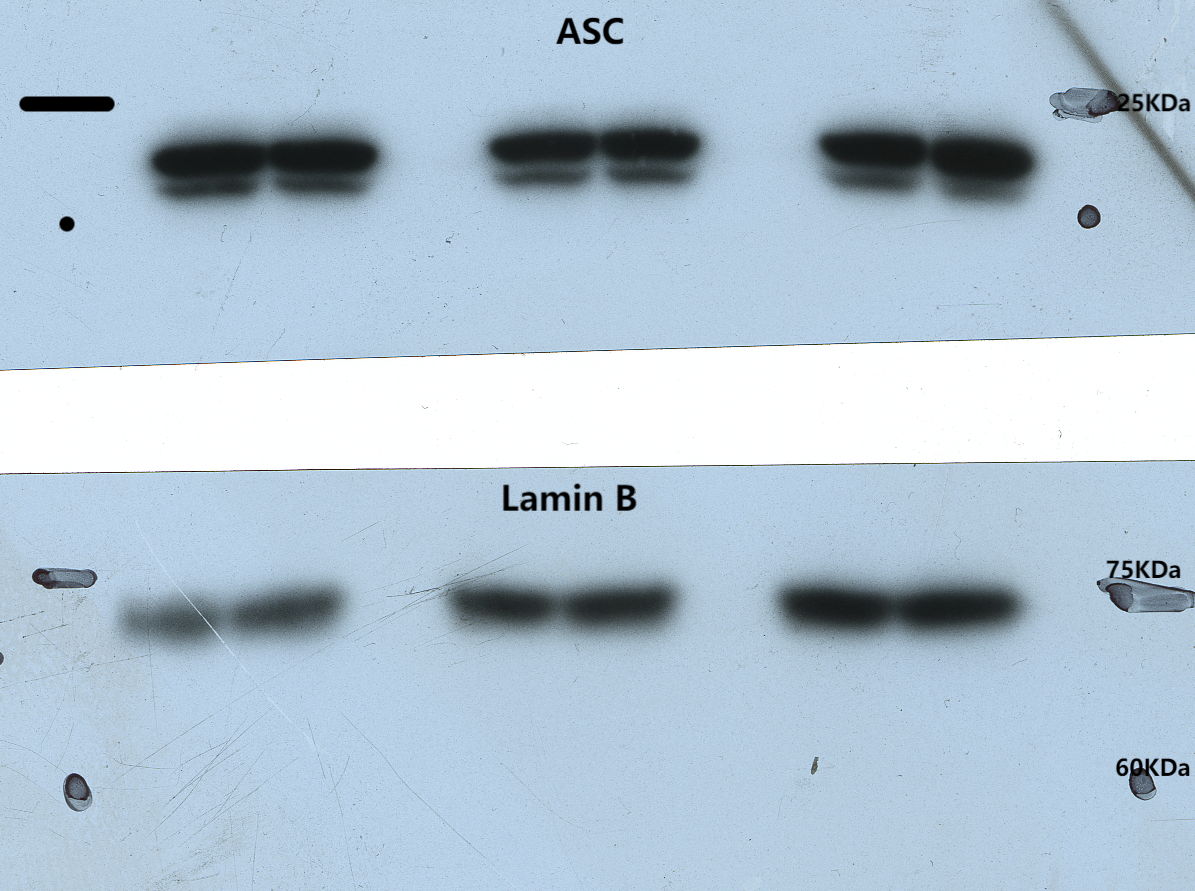

Supplement: Supplementary file 1 — Supplementary Material 1. Supplementary Figure 1. Licochalcone B derivatives inhibit NLRP3 inflammasome activation.BMDMs were primed with LPS for 4 hours, treated with Echinatin, Licorice Chalcone B, CTG4, CTG1, CTG10, CTG11, CTG12, CTG13, CTG14, CTG15, CTG16, CTG18, CTG19, CAPE, CTG23for 30 minutes, and then stimulated with nigericin for 25 minutes. Supernatants were collected for the measurement of caspase-1. Data represent as mean ± SEM. Compared to con, **** p < 0.0001; compared to a concentration of 0 μM, ###p < 0.001, #### p < 0.0001 and ns:not significant. Supplementary Figure 2. CTG11 and CTG13 inhibit NLRP3 inflammasome activation in mouse BMDMs.The structure of CTG11.Western blot analysis of IL-1β, caspase-1in culture supernatantsand pro-IL-1β, caspase-1, NLRP3, ASC in whole cell lysatesof LPS-primed BMDMs treated with CTG11 and then stimulated with Nigericin, supernatants were collected for the measurement of caspase-1, IL-1β, LDHand TNF-α.The structure of CTG13.Western blot analysis of IL-1β, caspase-1in culture supernatantsand pro-IL-1β, caspase-1, NLRP3, ASC in whole cell lysatesof LPS-primed BMDMs treated with CTG13 and then stimulated with Nigericin, supernatants were collected for the measurement of caspase-1, IL-1β, LDHand TNF-α. Coomassie blue–stained gels used as loading control and Lamin B used as a control for equal loading of the samples. Data represent as mean ± SEM. Compared to con, ** p < 0.01, ***p < 0.001, **** p < 0.0001; compared to a concentration of 0 μM, ###p < 0.001, ####p < 0.0001 and ns: not significant. Supplementary Figure 3. CTG12 impedes the priming process of NLRP3 inflammasome activation and specifically inhibits canonical and noncanonical NLRP3 inflammasome activation.BMDMs were primed with LPS treated with CTG12, then stimulated with Nigericin ATP, poly, or SiO₂. Supernatants were collected for the measurement of TNF-α, BMDMs primed with Pam3CSK4 treated with CTG12, followed by cytosolic LPS. Supernatants were collected fo [file 12964_2026_2741_MOESM1_ESM.zip › supplementary file/Figure4原膜/Figure4-D-ASC-Lamin B.png]

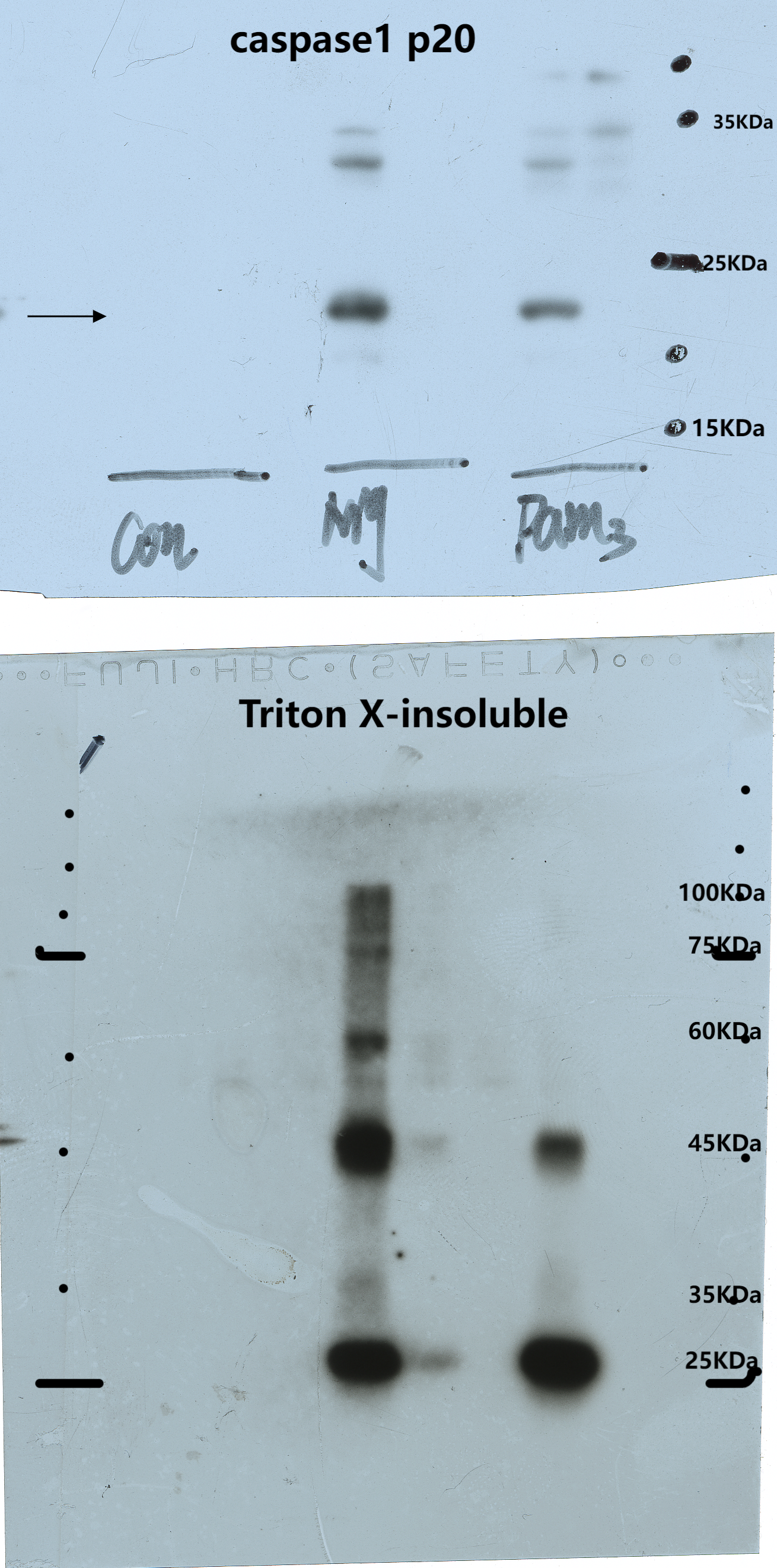

Supplement: Supplementary file 1 — Supplementary Material 1. Supplementary Figure 1. Licochalcone B derivatives inhibit NLRP3 inflammasome activation.BMDMs were primed with LPS for 4 hours, treated with Echinatin, Licorice Chalcone B, CTG4, CTG1, CTG10, CTG11, CTG12, CTG13, CTG14, CTG15, CTG16, CTG18, CTG19, CAPE, CTG23for 30 minutes, and then stimulated with nigericin for 25 minutes. Supernatants were collected for the measurement of caspase-1. Data represent as mean ± SEM. Compared to con, **** p < 0.0001; compared to a concentration of 0 μM, ###p < 0.001, #### p < 0.0001 and ns:not significant. Supplementary Figure 2. CTG11 and CTG13 inhibit NLRP3 inflammasome activation in mouse BMDMs.The structure of CTG11.Western blot analysis of IL-1β, caspase-1in culture supernatantsand pro-IL-1β, caspase-1, NLRP3, ASC in whole cell lysatesof LPS-primed BMDMs treated with CTG11 and then stimulated with Nigericin, supernatants were collected for the measurement of caspase-1, IL-1β, LDHand TNF-α.The structure of CTG13.Western blot analysis of IL-1β, caspase-1in culture supernatantsand pro-IL-1β, caspase-1, NLRP3, ASC in whole cell lysatesof LPS-primed BMDMs treated with CTG13 and then stimulated with Nigericin, supernatants were collected for the measurement of caspase-1, IL-1β, LDHand TNF-α. Coomassie blue–stained gels used as loading control and Lamin B used as a control for equal loading of the samples. Data represent as mean ± SEM. Compared to con, ** p < 0.01, ***p < 0.001, **** p < 0.0001; compared to a concentration of 0 μM, ###p < 0.001, ####p < 0.0001 and ns: not significant. Supplementary Figure 3. CTG12 impedes the priming process of NLRP3 inflammasome activation and specifically inhibits canonical and noncanonical NLRP3 inflammasome activation.BMDMs were primed with LPS treated with CTG12, then stimulated with Nigericin ATP, poly, or SiO₂. Supernatants were collected for the measurement of TNF-α, BMDMs primed with Pam3CSK4 treated with CTG12, followed by cytosolic LPS. Supernatants were collected fo [file 12964_2026_2741_MOESM1_ESM.zip › supplementary file/Figure4原膜/Figure4-D-caspase1p20-Triton X-insoluble.png]

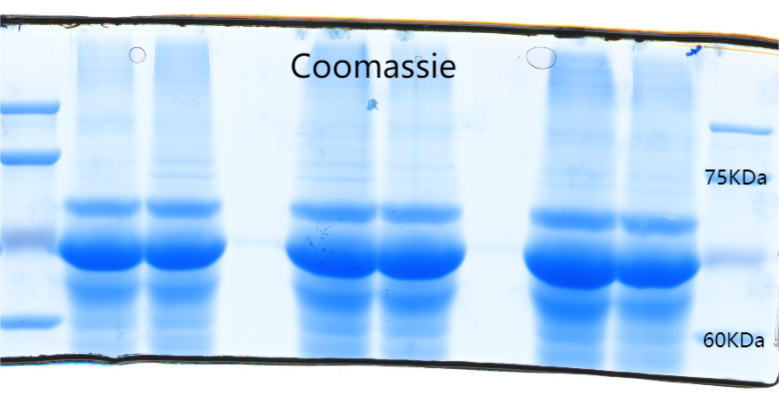

Supplement: Supplementary file 1 — Supplementary Material 1. Supplementary Figure 1. Licochalcone B derivatives inhibit NLRP3 inflammasome activation.BMDMs were primed with LPS for 4 hours, treated with Echinatin, Licorice Chalcone B, CTG4, CTG1, CTG10, CTG11, CTG12, CTG13, CTG14, CTG15, CTG16, CTG18, CTG19, CAPE, CTG23for 30 minutes, and then stimulated with nigericin for 25 minutes. Supernatants were collected for the measurement of caspase-1. Data represent as mean ± SEM. Compared to con, **** p < 0.0001; compared to a concentration of 0 μM, ###p < 0.001, #### p < 0.0001 and ns:not significant. Supplementary Figure 2. CTG11 and CTG13 inhibit NLRP3 inflammasome activation in mouse BMDMs.The structure of CTG11.Western blot analysis of IL-1β, caspase-1in culture supernatantsand pro-IL-1β, caspase-1, NLRP3, ASC in whole cell lysatesof LPS-primed BMDMs treated with CTG11 and then stimulated with Nigericin, supernatants were collected for the measurement of caspase-1, IL-1β, LDHand TNF-α.The structure of CTG13.Western blot analysis of IL-1β, caspase-1in culture supernatantsand pro-IL-1β, caspase-1, NLRP3, ASC in whole cell lysatesof LPS-primed BMDMs treated with CTG13 and then stimulated with Nigericin, supernatants were collected for the measurement of caspase-1, IL-1β, LDHand TNF-α. Coomassie blue–stained gels used as loading control and Lamin B used as a control for equal loading of the samples. Data represent as mean ± SEM. Compared to con, ** p < 0.01, ***p < 0.001, **** p < 0.0001; compared to a concentration of 0 μM, ###p < 0.001, ####p < 0.0001 and ns: not significant. Supplementary Figure 3. CTG12 impedes the priming process of NLRP3 inflammasome activation and specifically inhibits canonical and noncanonical NLRP3 inflammasome activation.BMDMs were primed with LPS treated with CTG12, then stimulated with Nigericin ATP, poly, or SiO₂. Supernatants were collected for the measurement of TNF-α, BMDMs primed with Pam3CSK4 treated with CTG12, followed by cytosolic LPS. Supernatants were collected fo [file 12964_2026_2741_MOESM1_ESM.zip › supplementary file/Figure4原膜/Figure4-D-Coomassie.png]

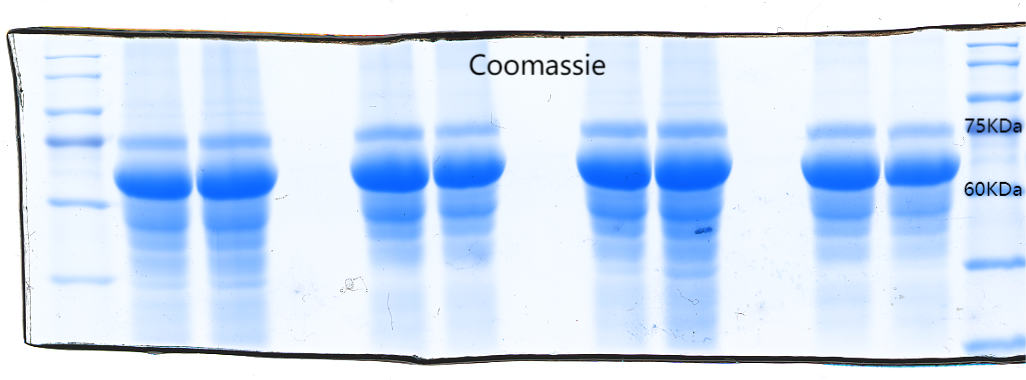

Supplement: Supplementary file 1 — Supplementary Material 1. Supplementary Figure 1. Licochalcone B derivatives inhibit NLRP3 inflammasome activation.BMDMs were primed with LPS for 4 hours, treated with Echinatin, Licorice Chalcone B, CTG4, CTG1, CTG10, CTG11, CTG12, CTG13, CTG14, CTG15, CTG16, CTG18, CTG19, CAPE, CTG23for 30 minutes, and then stimulated with nigericin for 25 minutes. Supernatants were collected for the measurement of caspase-1. Data represent as mean ± SEM. Compared to con, **** p < 0.0001; compared to a concentration of 0 μM, ###p < 0.001, #### p < 0.0001 and ns:not significant. Supplementary Figure 2. CTG11 and CTG13 inhibit NLRP3 inflammasome activation in mouse BMDMs.The structure of CTG11.Western blot analysis of IL-1β, caspase-1in culture supernatantsand pro-IL-1β, caspase-1, NLRP3, ASC in whole cell lysatesof LPS-primed BMDMs treated with CTG11 and then stimulated with Nigericin, supernatants were collected for the measurement of caspase-1, IL-1β, LDHand TNF-α.The structure of CTG13.Western blot analysis of IL-1β, caspase-1in culture supernatantsand pro-IL-1β, caspase-1, NLRP3, ASC in whole cell lysatesof LPS-primed BMDMs treated with CTG13 and then stimulated with Nigericin, supernatants were collected for the measurement of caspase-1, IL-1β, LDHand TNF-α. Coomassie blue–stained gels used as loading control and Lamin B used as a control for equal loading of the samples. Data represent as mean ± SEM. Compared to con, ** p < 0.01, ***p < 0.001, **** p < 0.0001; compared to a concentration of 0 μM, ###p < 0.001, ####p < 0.0001 and ns: not significant. Supplementary Figure 3. CTG12 impedes the priming process of NLRP3 inflammasome activation and specifically inhibits canonical and noncanonical NLRP3 inflammasome activation.BMDMs were primed with LPS treated with CTG12, then stimulated with Nigericin ATP, poly, or SiO₂. Supernatants were collected for the measurement of TNF-α, BMDMs primed with Pam3CSK4 treated with CTG12, followed by cytosolic LPS. Supernatants were collected fo [file 12964_2026_2741_MOESM1_ESM.zip › supplementary file/Figure4原膜/Figure4-E-Coomassie.png]

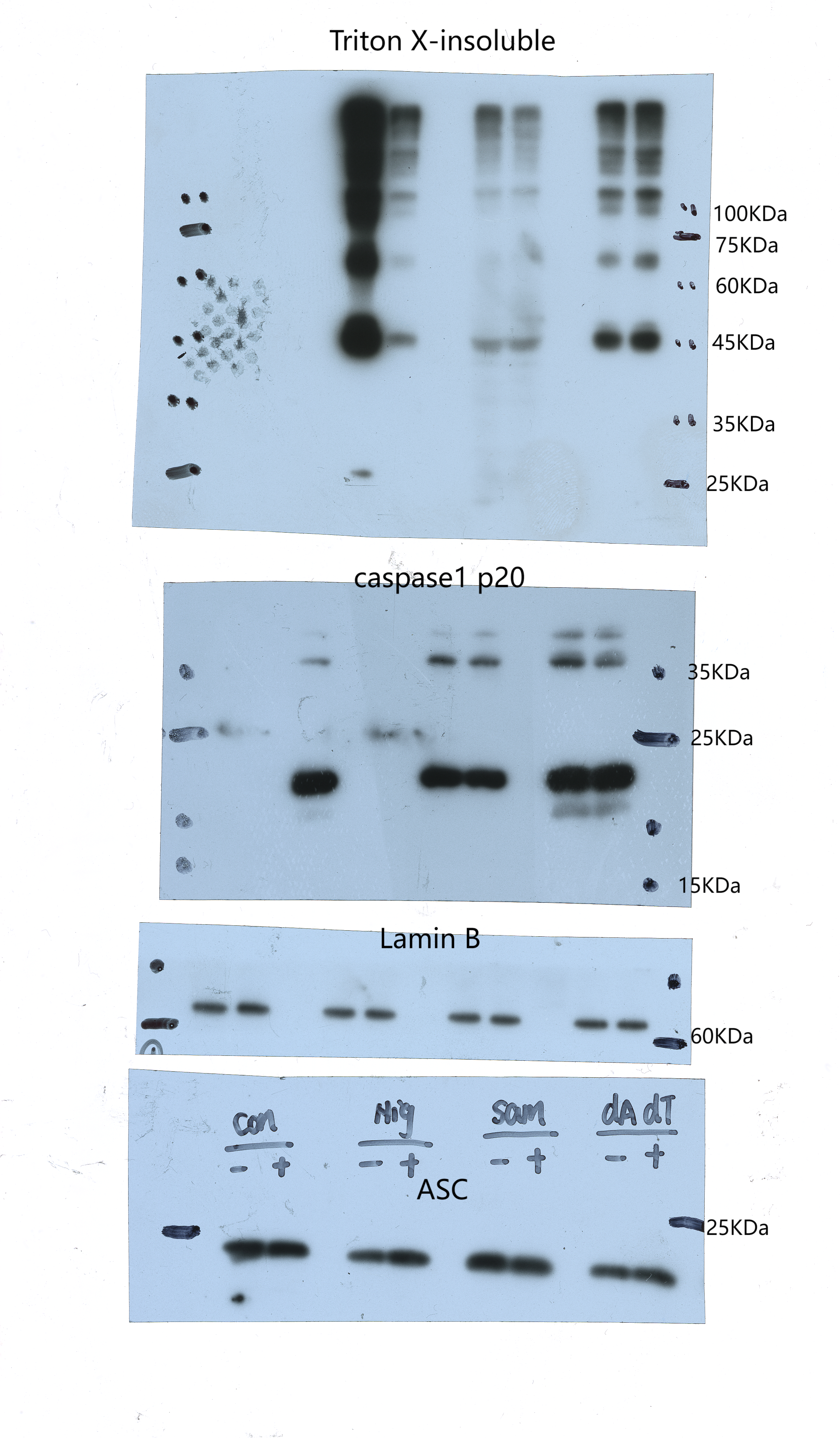

Supplement: Supplementary file 1 — Supplementary Material 1. Supplementary Figure 1. Licochalcone B derivatives inhibit NLRP3 inflammasome activation.BMDMs were primed with LPS for 4 hours, treated with Echinatin, Licorice Chalcone B, CTG4, CTG1, CTG10, CTG11, CTG12, CTG13, CTG14, CTG15, CTG16, CTG18, CTG19, CAPE, CTG23for 30 minutes, and then stimulated with nigericin for 25 minutes. Supernatants were collected for the measurement of caspase-1. Data represent as mean ± SEM. Compared to con, **** p < 0.0001; compared to a concentration of 0 μM, ###p < 0.001, #### p < 0.0001 and ns:not significant. Supplementary Figure 2. CTG11 and CTG13 inhibit NLRP3 inflammasome activation in mouse BMDMs.The structure of CTG11.Western blot analysis of IL-1β, caspase-1in culture supernatantsand pro-IL-1β, caspase-1, NLRP3, ASC in whole cell lysatesof LPS-primed BMDMs treated with CTG11 and then stimulated with Nigericin, supernatants were collected for the measurement of caspase-1, IL-1β, LDHand TNF-α.The structure of CTG13.Western blot analysis of IL-1β, caspase-1in culture supernatantsand pro-IL-1β, caspase-1, NLRP3, ASC in whole cell lysatesof LPS-primed BMDMs treated with CTG13 and then stimulated with Nigericin, supernatants were collected for the measurement of caspase-1, IL-1β, LDHand TNF-α. Coomassie blue–stained gels used as loading control and Lamin B used as a control for equal loading of the samples. Data represent as mean ± SEM. Compared to con, ** p < 0.01, ***p < 0.001, **** p < 0.0001; compared to a concentration of 0 μM, ###p < 0.001, ####p < 0.0001 and ns: not significant. Supplementary Figure 3. CTG12 impedes the priming process of NLRP3 inflammasome activation and specifically inhibits canonical and noncanonical NLRP3 inflammasome activation.BMDMs were primed with LPS treated with CTG12, then stimulated with Nigericin ATP, poly, or SiO₂. Supernatants were collected for the measurement of TNF-α, BMDMs primed with Pam3CSK4 treated with CTG12, followed by cytosolic LPS. Supernatants were collected fo [file 12964_2026_2741_MOESM1_ESM.zip › supplementary file/Figure4原膜/Figure4-E-Triton X-insoluble-caspase1p20-ASC-Lamin B.png]

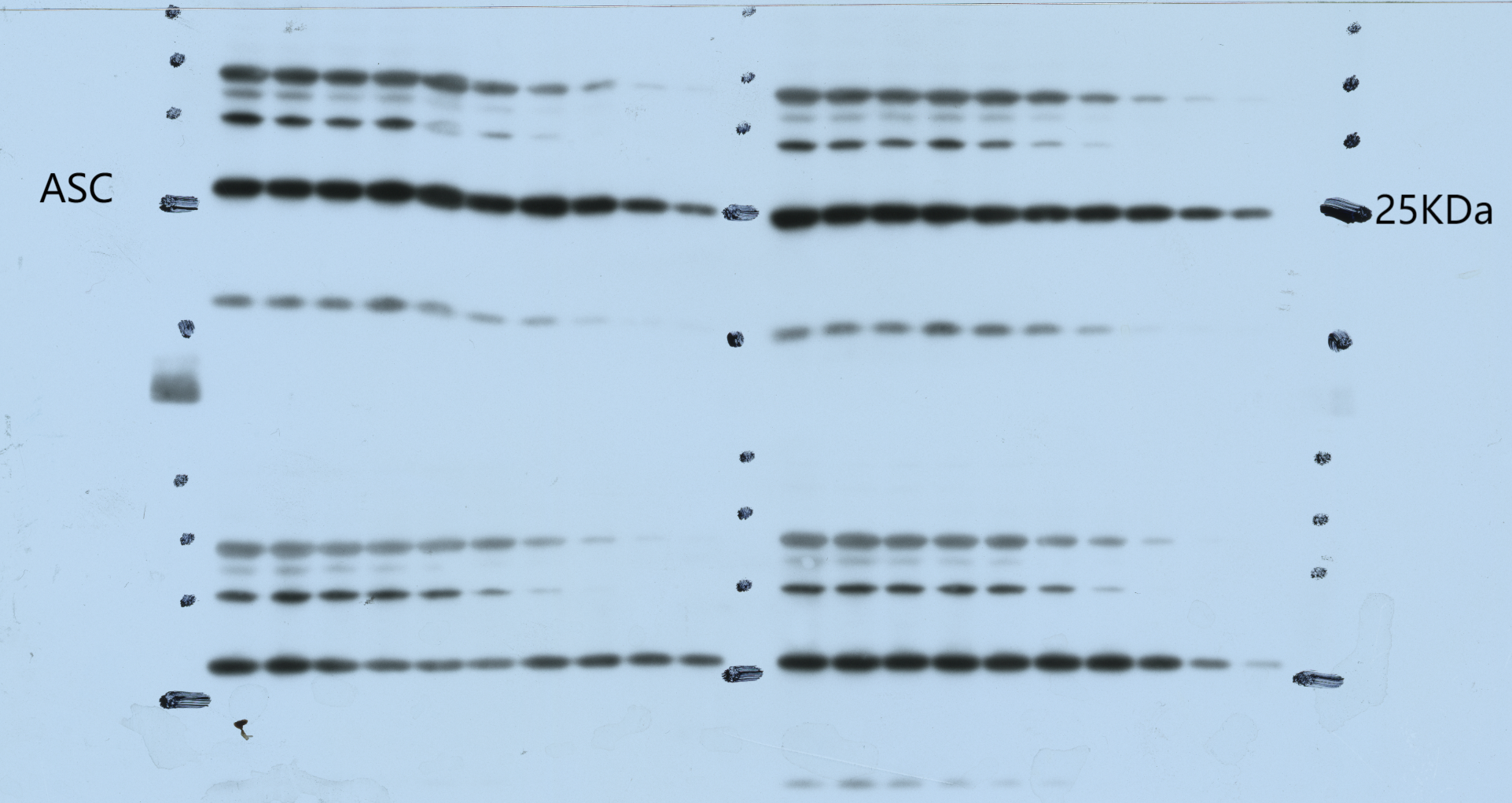

Supplement: Supplementary file 1 — Supplementary Material 1. Supplementary Figure 1. Licochalcone B derivatives inhibit NLRP3 inflammasome activation.BMDMs were primed with LPS for 4 hours, treated with Echinatin, Licorice Chalcone B, CTG4, CTG1, CTG10, CTG11, CTG12, CTG13, CTG14, CTG15, CTG16, CTG18, CTG19, CAPE, CTG23for 30 minutes, and then stimulated with nigericin for 25 minutes. Supernatants were collected for the measurement of caspase-1. Data represent as mean ± SEM. Compared to con, **** p < 0.0001; compared to a concentration of 0 μM, ###p < 0.001, #### p < 0.0001 and ns:not significant. Supplementary Figure 2. CTG11 and CTG13 inhibit NLRP3 inflammasome activation in mouse BMDMs.The structure of CTG11.Western blot analysis of IL-1β, caspase-1in culture supernatantsand pro-IL-1β, caspase-1, NLRP3, ASC in whole cell lysatesof LPS-primed BMDMs treated with CTG11 and then stimulated with Nigericin, supernatants were collected for the measurement of caspase-1, IL-1β, LDHand TNF-α.The structure of CTG13.Western blot analysis of IL-1β, caspase-1in culture supernatantsand pro-IL-1β, caspase-1, NLRP3, ASC in whole cell lysatesof LPS-primed BMDMs treated with CTG13 and then stimulated with Nigericin, supernatants were collected for the measurement of caspase-1, IL-1β, LDHand TNF-α. Coomassie blue–stained gels used as loading control and Lamin B used as a control for equal loading of the samples. Data represent as mean ± SEM. Compared to con, ** p < 0.01, ***p < 0.001, **** p < 0.0001; compared to a concentration of 0 μM, ###p < 0.001, ####p < 0.0001 and ns: not significant. Supplementary Figure 3. CTG12 impedes the priming process of NLRP3 inflammasome activation and specifically inhibits canonical and noncanonical NLRP3 inflammasome activation.BMDMs were primed with LPS treated with CTG12, then stimulated with Nigericin ATP, poly, or SiO₂. Supernatants were collected for the measurement of TNF-α, BMDMs primed with Pam3CSK4 treated with CTG12, followed by cytosolic LPS. Supernatants were collected fo [file 12964_2026_2741_MOESM1_ESM.zip › supplementary file/Figure5原膜/Figure5-D-ASC.png]

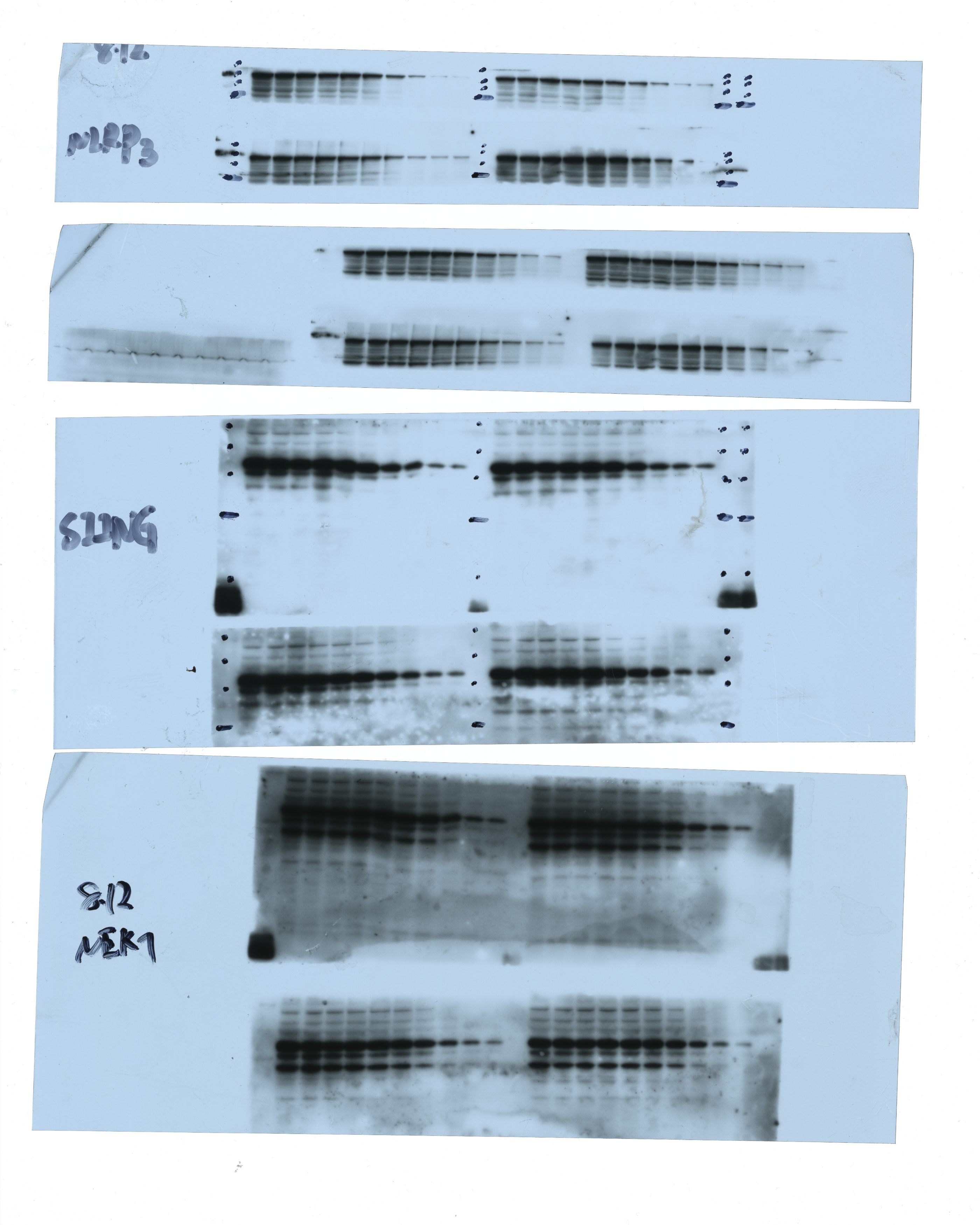

Supplement: Supplementary file 1 — Supplementary Material 1. Supplementary Figure 1. Licochalcone B derivatives inhibit NLRP3 inflammasome activation.BMDMs were primed with LPS for 4 hours, treated with Echinatin, Licorice Chalcone B, CTG4, CTG1, CTG10, CTG11, CTG12, CTG13, CTG14, CTG15, CTG16, CTG18, CTG19, CAPE, CTG23for 30 minutes, and then stimulated with nigericin for 25 minutes. Supernatants were collected for the measurement of caspase-1. Data represent as mean ± SEM. Compared to con, **** p < 0.0001; compared to a concentration of 0 μM, ###p < 0.001, #### p < 0.0001 and ns:not significant. Supplementary Figure 2. CTG11 and CTG13 inhibit NLRP3 inflammasome activation in mouse BMDMs.The structure of CTG11.Western blot analysis of IL-1β, caspase-1in culture supernatantsand pro-IL-1β, caspase-1, NLRP3, ASC in whole cell lysatesof LPS-primed BMDMs treated with CTG11 and then stimulated with Nigericin, supernatants were collected for the measurement of caspase-1, IL-1β, LDHand TNF-α.The structure of CTG13.Western blot analysis of IL-1β, caspase-1in culture supernatantsand pro-IL-1β, caspase-1, NLRP3, ASC in whole cell lysatesof LPS-primed BMDMs treated with CTG13 and then stimulated with Nigericin, supernatants were collected for the measurement of caspase-1, IL-1β, LDHand TNF-α. Coomassie blue–stained gels used as loading control and Lamin B used as a control for equal loading of the samples. Data represent as mean ± SEM. Compared to con, ** p < 0.01, ***p < 0.001, **** p < 0.0001; compared to a concentration of 0 μM, ###p < 0.001, ####p < 0.0001 and ns: not significant. Supplementary Figure 3. CTG12 impedes the priming process of NLRP3 inflammasome activation and specifically inhibits canonical and noncanonical NLRP3 inflammasome activation.BMDMs were primed with LPS treated with CTG12, then stimulated with Nigericin ATP, poly, or SiO₂. Supernatants were collected for the measurement of TNF-α, BMDMs primed with Pam3CSK4 treated with CTG12, followed by cytosolic LPS. Supernatants were collected fo [file 12964_2026_2741_MOESM1_ESM.zip › supplementary file/Figure5原膜/Figure5-D-NLRP3-STING-NEK7.tif]

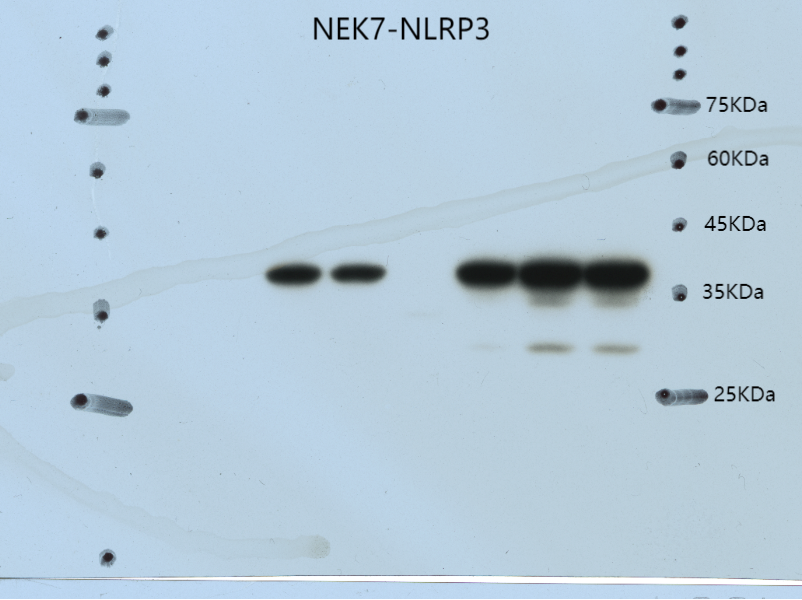

Supplement: Supplementary file 1 — Supplementary Material 1. Supplementary Figure 1. Licochalcone B derivatives inhibit NLRP3 inflammasome activation.BMDMs were primed with LPS for 4 hours, treated with Echinatin, Licorice Chalcone B, CTG4, CTG1, CTG10, CTG11, CTG12, CTG13, CTG14, CTG15, CTG16, CTG18, CTG19, CAPE, CTG23for 30 minutes, and then stimulated with nigericin for 25 minutes. Supernatants were collected for the measurement of caspase-1. Data represent as mean ± SEM. Compared to con, **** p < 0.0001; compared to a concentration of 0 μM, ###p < 0.001, #### p < 0.0001 and ns:not significant. Supplementary Figure 2. CTG11 and CTG13 inhibit NLRP3 inflammasome activation in mouse BMDMs.The structure of CTG11.Western blot analysis of IL-1β, caspase-1in culture supernatantsand pro-IL-1β, caspase-1, NLRP3, ASC in whole cell lysatesof LPS-primed BMDMs treated with CTG11 and then stimulated with Nigericin, supernatants were collected for the measurement of caspase-1, IL-1β, LDHand TNF-α.The structure of CTG13.Western blot analysis of IL-1β, caspase-1in culture supernatantsand pro-IL-1β, caspase-1, NLRP3, ASC in whole cell lysatesof LPS-primed BMDMs treated with CTG13 and then stimulated with Nigericin, supernatants were collected for the measurement of caspase-1, IL-1β, LDHand TNF-α. Coomassie blue–stained gels used as loading control and Lamin B used as a control for equal loading of the samples. Data represent as mean ± SEM. Compared to con, ** p < 0.01, ***p < 0.001, **** p < 0.0001; compared to a concentration of 0 μM, ###p < 0.001, ####p < 0.0001 and ns: not significant. Supplementary Figure 3. CTG12 impedes the priming process of NLRP3 inflammasome activation and specifically inhibits canonical and noncanonical NLRP3 inflammasome activation.BMDMs were primed with LPS treated with CTG12, then stimulated with Nigericin ATP, poly, or SiO₂. Supernatants were collected for the measurement of TNF-α, BMDMs primed with Pam3CSK4 treated with CTG12, followed by cytosolic LPS. Supernatants were collected fo [file 12964_2026_2741_MOESM1_ESM.zip › supplementary file/Figure5原膜/Figure5-E-NEK7.png]

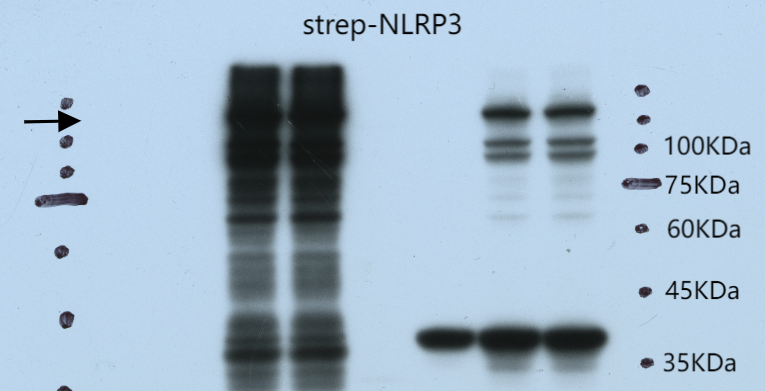

Supplement: Supplementary file 1 — Supplementary Material 1. Supplementary Figure 1. Licochalcone B derivatives inhibit NLRP3 inflammasome activation.BMDMs were primed with LPS for 4 hours, treated with Echinatin, Licorice Chalcone B, CTG4, CTG1, CTG10, CTG11, CTG12, CTG13, CTG14, CTG15, CTG16, CTG18, CTG19, CAPE, CTG23for 30 minutes, and then stimulated with nigericin for 25 minutes. Supernatants were collected for the measurement of caspase-1. Data represent as mean ± SEM. Compared to con, **** p < 0.0001; compared to a concentration of 0 μM, ###p < 0.001, #### p < 0.0001 and ns:not significant. Supplementary Figure 2. CTG11 and CTG13 inhibit NLRP3 inflammasome activation in mouse BMDMs.The structure of CTG11.Western blot analysis of IL-1β, caspase-1in culture supernatantsand pro-IL-1β, caspase-1, NLRP3, ASC in whole cell lysatesof LPS-primed BMDMs treated with CTG11 and then stimulated with Nigericin, supernatants were collected for the measurement of caspase-1, IL-1β, LDHand TNF-α.The structure of CTG13.Western blot analysis of IL-1β, caspase-1in culture supernatantsand pro-IL-1β, caspase-1, NLRP3, ASC in whole cell lysatesof LPS-primed BMDMs treated with CTG13 and then stimulated with Nigericin, supernatants were collected for the measurement of caspase-1, IL-1β, LDHand TNF-α. Coomassie blue–stained gels used as loading control and Lamin B used as a control for equal loading of the samples. Data represent as mean ± SEM. Compared to con, ** p < 0.01, ***p < 0.001, **** p < 0.0001; compared to a concentration of 0 μM, ###p < 0.001, ####p < 0.0001 and ns: not significant. Supplementary Figure 3. CTG12 impedes the priming process of NLRP3 inflammasome activation and specifically inhibits canonical and noncanonical NLRP3 inflammasome activation.BMDMs were primed with LPS treated with CTG12, then stimulated with Nigericin ATP, poly, or SiO₂. Supernatants were collected for the measurement of TNF-α, BMDMs primed with Pam3CSK4 treated with CTG12, followed by cytosolic LPS. Supernatants were collected fo [file 12964_2026_2741_MOESM1_ESM.zip › supplementary file/Figure5原膜/Figure5-E-NLRP3.png]

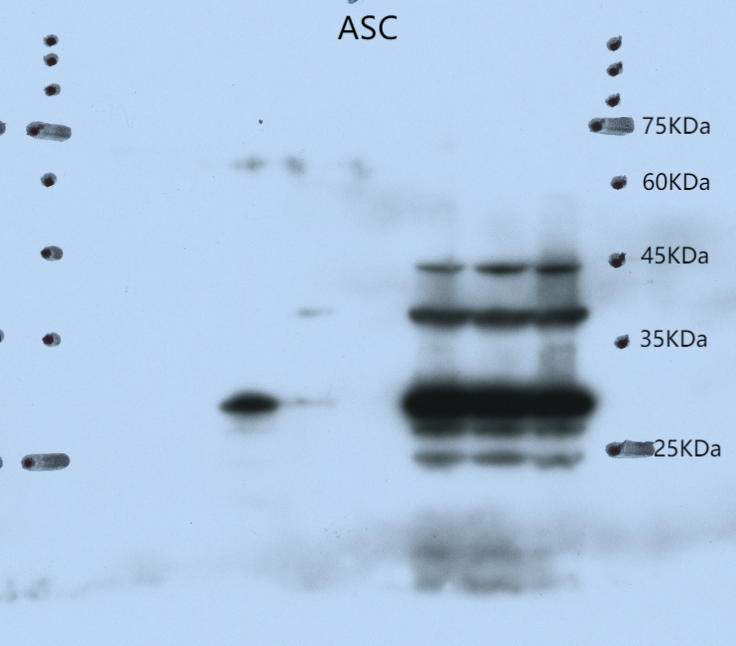

Supplement: Supplementary file 1 — Supplementary Material 1. Supplementary Figure 1. Licochalcone B derivatives inhibit NLRP3 inflammasome activation.BMDMs were primed with LPS for 4 hours, treated with Echinatin, Licorice Chalcone B, CTG4, CTG1, CTG10, CTG11, CTG12, CTG13, CTG14, CTG15, CTG16, CTG18, CTG19, CAPE, CTG23for 30 minutes, and then stimulated with nigericin for 25 minutes. Supernatants were collected for the measurement of caspase-1. Data represent as mean ± SEM. Compared to con, **** p < 0.0001; compared to a concentration of 0 μM, ###p < 0.001, #### p < 0.0001 and ns:not significant. Supplementary Figure 2. CTG11 and CTG13 inhibit NLRP3 inflammasome activation in mouse BMDMs.The structure of CTG11.Western blot analysis of IL-1β, caspase-1in culture supernatantsand pro-IL-1β, caspase-1, NLRP3, ASC in whole cell lysatesof LPS-primed BMDMs treated with CTG11 and then stimulated with Nigericin, supernatants were collected for the measurement of caspase-1, IL-1β, LDHand TNF-α.The structure of CTG13.Western blot analysis of IL-1β, caspase-1in culture supernatantsand pro-IL-1β, caspase-1, NLRP3, ASC in whole cell lysatesof LPS-primed BMDMs treated with CTG13 and then stimulated with Nigericin, supernatants were collected for the measurement of caspase-1, IL-1β, LDHand TNF-α. Coomassie blue–stained gels used as loading control and Lamin B used as a control for equal loading of the samples. Data represent as mean ± SEM. Compared to con, ** p < 0.01, ***p < 0.001, **** p < 0.0001; compared to a concentration of 0 μM, ###p < 0.001, ####p < 0.0001 and ns: not significant. Supplementary Figure 3. CTG12 impedes the priming process of NLRP3 inflammasome activation and specifically inhibits canonical and noncanonical NLRP3 inflammasome activation.BMDMs were primed with LPS treated with CTG12, then stimulated with Nigericin ATP, poly, or SiO₂. Supernatants were collected for the measurement of TNF-α, BMDMs primed with Pam3CSK4 treated with CTG12, followed by cytosolic LPS. Supernatants were collected fo [file 12964_2026_2741_MOESM1_ESM.zip › supplementary file/Figure5原膜/Figure5-F-ASC.png]

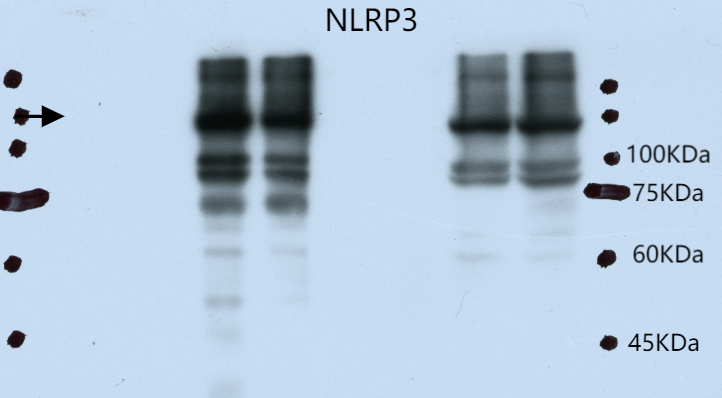

Supplement: Supplementary file 1 — Supplementary Material 1. Supplementary Figure 1. Licochalcone B derivatives inhibit NLRP3 inflammasome activation.BMDMs were primed with LPS for 4 hours, treated with Echinatin, Licorice Chalcone B, CTG4, CTG1, CTG10, CTG11, CTG12, CTG13, CTG14, CTG15, CTG16, CTG18, CTG19, CAPE, CTG23for 30 minutes, and then stimulated with nigericin for 25 minutes. Supernatants were collected for the measurement of caspase-1. Data represent as mean ± SEM. Compared to con, **** p < 0.0001; compared to a concentration of 0 μM, ###p < 0.001, #### p < 0.0001 and ns:not significant. Supplementary Figure 2. CTG11 and CTG13 inhibit NLRP3 inflammasome activation in mouse BMDMs.The structure of CTG11.Western blot analysis of IL-1β, caspase-1in culture supernatantsand pro-IL-1β, caspase-1, NLRP3, ASC in whole cell lysatesof LPS-primed BMDMs treated with CTG11 and then stimulated with Nigericin, supernatants were collected for the measurement of caspase-1, IL-1β, LDHand TNF-α.The structure of CTG13.Western blot analysis of IL-1β, caspase-1in culture supernatantsand pro-IL-1β, caspase-1, NLRP3, ASC in whole cell lysatesof LPS-primed BMDMs treated with CTG13 and then stimulated with Nigericin, supernatants were collected for the measurement of caspase-1, IL-1β, LDHand TNF-α. Coomassie blue–stained gels used as loading control and Lamin B used as a control for equal loading of the samples. Data represent as mean ± SEM. Compared to con, ** p < 0.01, ***p < 0.001, **** p < 0.0001; compared to a concentration of 0 μM, ###p < 0.001, ####p < 0.0001 and ns: not significant. Supplementary Figure 3. CTG12 impedes the priming process of NLRP3 inflammasome activation and specifically inhibits canonical and noncanonical NLRP3 inflammasome activation.BMDMs were primed with LPS treated with CTG12, then stimulated with Nigericin ATP, poly, or SiO₂. Supernatants were collected for the measurement of TNF-α, BMDMs primed with Pam3CSK4 treated with CTG12, followed by cytosolic LPS. Supernatants were collected fo [file 12964_2026_2741_MOESM1_ESM.zip › supplementary file/Figure5原膜/Figure5-F-NLRP3.png]
